# Insulin regulates lymphatic endothelial integrity via palmitoylation

**DOI:** 10.1016/j.jlr.2025.100775

**Published:** 2025-03-11

**Authors:** Silvia Gonzalez-Nieves, Xiaochao Wei, Simon Guignard, Thi Nguyen, Jay McQuillan, Qiang Zhang, Jinsong Zhang, Reagan M. McGuffee, David A. Ford, Clay F. Semenkovich, Vincenza Cifarelli

**Affiliations:** 1 Department of Pharmacology and Physiology, Saint Louis University School of Medicine, St. Louis, MO, USA; 2 Division of Endocrinology Metabolism and Lipid Research, Department of Medicine, Washington University, St. Louis, MO, USA; 3 Department of Biochemistry and Molecular Biology, Saint Louis University School of Medicine, St. Louis, MO, USA; 4 Department of Cell Biology and Physiology, Washington University, St. Louis, MO, USA

**Keywords:** insulin, LECs, palmitoylation, CD36, claudin 5, CD63

## Abstract

Lipid metabolism plays a critical role in lymphatic endothelial cell (LEC) development and vessel maintenance. Altered lipid metabolism is associated with loss of lymphatic vessel integrity, which compromises organ function, protective immunity, and metabolic health. Thus, understanding how lipid metabolism affects LECs is critical for uncovering the mechanisms underlying lymphatic dysfunction. Protein palmitoylation, a lipid-based post-translational modification, has emerged as a critical regulator of protein function, stability, and interaction networks. Insulin, a master regulator of systemic lipid metabolism, also regulates protein palmitoylation. However, the role of insulin-driven palmitoylation in LEC biology remains unexplored. To examine the role of palmitoylation in LEC function, we generated the first palmitoylation proteomics profile in human LECs, validated insulin-regulated targets, and determined the role of palmitoylation in LEC barrier function. In unstimulated conditions, palmitoylation occurred primarily on proteins involved in vesicular and membrane trafficking, and in translation initiation. Insulin treatment, instead, enriched palmitoylation of proteins involved in LEC integrity, namely junctional proteins such as claudin 5, along with small GTPases and ubiquitination enzymes. We also investigated the role of the long-chain fatty acid transporter CD36, a major mediator of palmitate uptake into cells, in regulating optimal lymphatic protein palmitoylation. CD36 silencing in LECs increased by 2-fold palmitoylation of proteins involved in inflammation and immune cell activation. Overall, our findings provide novel insights into the intricate relationship between lipid modification and LEC function, suggesting that insulin and palmitoylation play a critical role in lymphatic endothelial function.

The lymphatic system and lipid metabolism share a reciprocal regulation essential for the homeostasis of vessel health and systemic metabolism (1). Gut lymphatics mediate the entry of dietary lipids absorbed in the intestine via chylomicrons into the bloodstream for systemic distribution (2). Lymphatic endothelial cells (LECs) rely on lipid metabolism, ie, fatty acid β-oxidation to grow and support their energy needs (3), signaling functions (4), and vessel identity (5). Accordingly, fatty acid β-oxidation in lymphatic vessels is sustained by a robust expression of genes involved in lipid transport and metabolism (5, 6). Altered lipid homeostasis, as seen in metabolic disorders, impairs lymphatic vessel integrity and function promoting extravasation of immune cells, improper lipid distribution, and accumulation in tissues, contributing to metabolic (6, 7, 8, 9) and cardiovascular diseases (10). Although the link between lipid metabolism and lymphatic function is well established, the molecular and cellular mechanisms regulating this relationship are not well understood.

Lipids can affect cellular function via posttranslational modifications of proteins, namely, myristoylation, prenylation, and palmitoylation (11). Protein lipidation increases the hydrophobicity of proteins, resulting in changes in protein conformation, stability, membrane association, trafficking, and binding to co-factors. Among the different types of lipidation, palmitoylation is the only reversible post-translational lipid modification linking the long-chain fatty acid (FA) palmitate to a cysteine amino acid. Reversible palmitoylation confers spatiotemporal control of protein function by modulating protein stability, trafficking, and activity, as well as protein–protein and membrane-protein interactions (12). Palmitoylation is catalyzed by approximately 20 aspartate-histidine-histidine-cysteine acyltransferases (known as DHHCs), many of which are expressed in endothelial cells (13). Depalmitoylation is performed by a family of enzymes including acyl-protein thioesterase 1 and 2 (APT-1, 2) (14) and α/β-Hydrolase Domain proteins (ABHD) 17-A, B and C (15). Altered palmitoylation has been reported in pathological vascular remodeling (13, 16, 17, 18); however, the role of palmitoylation in LEC function is not known.

Insulin is a strong regulator of lipid metabolism (19) and of protein palmitoylation (16). Human LECs are highly sensitive to insulin stimulation in vitro initiating the PI3K/AKT signaling cascade at lower insulin doses than in blood endothelial cells (20). Hyperglycemia and hyperinsulinemia can lead to insulin resistance in cultured LECs, blunting AKT signaling, which is associated with compromised integrity of the LEC monolayer and inflammation (21). These in vitro findings are in line with reports in rodents showing that the metabolic syndrome is associated with impaired lymphatic function and reduced vessel integrity (9, 22). However, the targets of insulin action in lymphatics are largely unknown, and protein palmitoylation in LECs has not been studied.

In the present study, we determined the role of palmitoylation in LEC biology and barrier function, a critical property for lymphatic vessels to maintain tissue fluid balance, immune cell trafficking, and lipid transport. We generated the first quantitative proteomic profile of palmitoylation in cultured human LECs at baseline and following insulin stimulation, validated insulin-regulated targets, and determined the role of palmitoylation on cell-cell junctional remodeling and LEC integrity. We also investigated the role of the long-chain fatty acid transporter CD36, a major mediator of palmitate uptake into cells, in regulating these processes. Overall, our results provide a comprehensive characterization of the palmitoylation profile in human LECs under different stimuli and shed light on novel mechanisms linking insulin-mediated palmitoylation to LEC function.

## Materials and Methods

### Cell culture and treatments

Primary human dermal lymphatic endothelial cells (hereafter referred to as LECs) were obtained from PromoCell and grown in EGM-2MV (microvascular endothelial cell growth medium-2) medium from Lonza Bioscience and used between passages 2 and 8 for experiments as previously described (6). Cells were serum starved (0.5% FBS) for 1 h and treated with insulin (100 nM; Sigma) or left untreated for 6 h at 37°C for proteomics experiments. All experiments were performed with 100 nM insulin, a concentration shown to regulate protein palmitoylation (16). For siRNA treatment, the LECs were grown to 60%–80% confluence and transfected for 48 h with either Silencer® Negative control (Invitrogen, cat. n 4,390,843) or CD36 Silencer® pre-designed siRNA (Invitrogen, car. N4392420, assay ID s2647) as per the manufacturer's instructions (ThermoFisher Scientific). Cells were then serum-starved for 1 h before treatment with media, insulin (100 nM), or cyano-myracrylamide (CMA, 20 μM for 6 h) (Cayman Chemical), a palmitoylation inhibitor (23), or CMA and insulin together. The concentration of CMA was chosen based on previous studies (23).

### Acyl-RAC assays for protein palmitoylation

S-palmitoylated proteins were purified using a modified acyl-RAC (resin-assisted capture) assay as previously described (17). Cells were homogenized in lysis buffer (150 mM NaCl, 50 mM Tris, 5 mM EDTA, 2% Triton-X-100, 0.2 mM HDSF, pH 7.4, and protease inhibitor cocktail from Roche). Following sonication on ice, protein concentrations were determined by BCA protein assay kits (ThermoFisher), and the same amounts of protein lysates were diluted to 2.5% SDS with 0.1% MMTS (S-Methyl methanethiosulfonate), and incubated at 37°C for 20 min. Proteins were then precipitated with ice-cold acetone (1:3 v/v protein solution: acetone) at −20°C for 30 min. Protein precipitates were obtained by centrifugation at 12,000 rpm for 10 min, washed 3 times with cold 70% acetone, and air dried. Pellets were resuspended with a binding buffer (100 mM HEPES, 1 mM EDTA, 1% SDS, pH 7.4, and protease inhibitor cocktail from Roche) by vortexing for 20 min at room temperature (RT). Solubilized fractions were obtained by centrifugation at 14,000 rpm for 5 min, and supernatants were then aliquoted into 2 tubes containing thiopropyl sepharose beads (GE Healthcare Life Sciences), with or without freshly prepared HA (hydroxylamine 0.8 M, pH 7.4), and rotated at room temperature for 2 h. The beads were washed 5 times with a binding buffer, and enriched proteins were eluted with a binding buffer containing 50 mM DTT (20 min at RT). Palmitoylated proteins were analyzed by Western blotting using Licor infrared fluorescence imaging.

### Proteomics

#### Peptide preparation

Peptides were prepared from eluates as previously described (24). Samples were reduced with 100 mM dithiothreitol at 95°C for 15 min, mixed with 200 μl of 100 mM Tris-HCL buffer, pH 8.5 containing 8 M urea (all from Sigma), transferred to the top of a 30,000 molecular weight cut-off filter (Millipore, part #MRCF0R030) and spun at 10,000 rcf for 10 min. An additional 300 μl of UA buffer was added and the filter was spun at 10,000 rcf for 10 min. The flow-through was discarded and the proteins were alkylated using 100 μl of 50 mM Iodoacetamide (IAM) in UA buffer. IAM in the UA buffer was added to the top chamber of the filtration unit. The samples were gyrated at 550 rpm using a Thermomixer (Eppendorf) at room temperature for 30 min in the dark. The filter was spun at 10,000 rcf for 10 min and the flow through discarded. Unreacted IAM was washed through the filter with two additions of UA buffer, and centrifugation at 10,000 rcf for 10 min after each buffer addition. The UA buffer was exchanged with the digestion buffer (DB, 50 mM ammonium bicarbonate). Two sequential additions of DB (200 μl) with centrifugation after each addition to the top chamber were performed. The filters were transferred to a new collection tube and samples digested with a combination of LysC (1 mAU per filter) and trypsin (1:50 wt/wt) in DB buffer on top of the filter for two hours and overnight at 37°C. The filters were spun at 14,000 rcf for 15 min to collect the peptides in the flow through. The filter was washed with 50 μl of 100 mM ammonium bicarbonate buffer and the wash was collected with the peptides. Residual detergent was removed by ethyl acetate extraction (25). In preparation for desalting, peptides were acidified to pH = 2 with 1% trifluoroacetic acid (TFA) at the final concentration. Peptides were desalted using stage tips (C18) (26), eluted with 60 μl of 60% (vol/vol) of MeCN in 0.1% (vol/vol) FA, and dried in a Speed-Vac (Thermo Scientific, Model No. Savant DNA 120 concentrator). Peptides were dissolved in 100 μl of 1% MeCN in water. An aliquot (10%) was removed for quantification using the Pierce Quantitative Fluorometric Peptide Assay kit. The remaining peptides were transferred to autosampler vials, dried, and stored at −80°C for LC-MS analysis.

#### Ultra-high performance liquid chromatography-mass spectrometry-timsTOF

The unlabeled peptides were analyzed using trapped ion mobility time-of-flight mass spectrometry (27). Peptides were separated using a *nano-ELUTE* ® chromatograph (Bruker Daltonics) interfaced to a timsTOF Pro mass spectrometer (Bruker Daltonics) with a modified nano-electrospray source (CaptiveSpray, Bruker Daltonics). The mass spectrometer was operated in PASEF mode (27). The samples in 2 μl of 1% (vol/vol) FA were injected onto a 75 μm i.d. × 25 cm Aurora Series column with a CSI emitter (Ionopticks). The column temperature was set to 50°C. The column was equilibrated using constant pressure (800 bar) with 8 column volumes of solvent A (0.1% (vol/vol) FA). Sample loading was performed at constant pressure (800 bar) at a volume of 1 sample pick-up volume plus 2 μl. The peptides were eluted using a column separation mode with a flow rate of 300 nl/min and using solvents A (0.1% (vol/vol) FA) and B (0.1% (vol/vol) FA/MeCN): solvent A containing 2% B increased to 17% B over 60 min, to 25% B over 30 min, to 37% B over 10 min, to 80% B over 10 min and constant 80% B for 10 min. The MS1 and MS2 spectra were recorded from *m/z* 100 to 1700. The collision energy was ramped stepwise as a function of increasing ion mobility: 52 eV for 0%–19% of the ramp time; 47 eV from 19%–38%; 42 eV from 38%–57%; 37 eV from 57%–76%; and 32 eV for the remainder. The TIMS elution voltage was calibrated linearly using the Agilent ESI-L Tuning Mix (*m/z* 622, 922, 1222).

#### MS data analysis

For timsTOF files, data from the mass spectrometer were converted to peak lists using DataAnalysis (version 5.2, Bruker Daltonics). MS2 spectra with parent ion charge states of +2, +3 and + 4 were analyzed using Mascot software (28) (Matrix Science; version 2.8.0.1) against a concatenated UniProt (Feburary 2022) database of human (20,512 entries) and common contaminant proteins (cRAP, version 1.0 Jan. 1st, 2012; 116 entries). Trypsin/P enzyme specificity with a maximum of 4 missed cleavages allowed was used. Label-free “single-shot” LC-MS data from the timsTOF mass spectrometer were searched with a fragment ion mass tolerance of 20 ppm and a parent ion tolerance of 20 ppm. Carbamidomethylation of cysteine was specified in Mascot as a fixed modification. Deamidation of asparagine, formation of pyro-glutamic acid from N-terminal glutamine, acetylation of protein N-terminus, and oxidation of methionine were specified as variable modifications. PSMs were filtered at a 1% false-discovery rate (FDR) by searching against a reversed database. A minimum of two peptides with unique sequences, not resulting from missed cleavages, was required for the identification of a protein. The processing, quality assurance, and analysis of LC-MS data were performed with proteoQ (version 1.7.5.1), part of the Mzion suite (29) software developed with the *tidyverse* approach with open-source software for statistical computing and graphics, R and R Studio. The precursor intensities were converted into logarithmic ratios (base 2), relative to the average precursor intensity across all samples. Within each sample, Dixon's outlier removals were carried out recursively for peptides with greater than two identifying PSMs. The median of the ratios of PSM that could be assigned to the same peptide was first taken to represent the ratios of the incumbent peptide. The median of the ratios of peptides was then taken to represent the ratios of the inferred protein. To align protein ratios across samples, likelihood functions were first estimated for the log ratios of proteins using finite mixture modeling, assuming two-component Gaussian mixtures (30). The ratio distributions were then aligned so that the maximum likelihood of log ratios was centered at zero for each sample. Scaling normalization was performed to standardize the log ratios of proteins across all samples. To reduce the influence of outliers from either log-ratios or reporter-ion intensities, the values between the 5^th^ and 95^th^ percentile of log-ratios and 5^th^ and 95^th^ percentile of intensity were used in the calculations of standard deviations.

#### Bioinformatic and statistical analysis

Metric multidimensional scaling (MDS) and Principal component analysis (PCA) of protein log2-ratios were performed with the base R function *stats:cmdscale* and stats:prcomp, respectively. Protein log2-ratios were visualized by volcano plot. Linear modeling was performed using the contrast fit approach in *limma* to assess the statistical significance of protein abundance differences between indicated groups of contrasts. Adjustments of *P*-values for multiple comparisons were used with Benjamini-Hochberg (BH) correction.

#### Pathway overrepresentation analysis

WebGestalt analysis tool kit was used to identify enriched molecular pathways based on protein palmitoylation enrichment in each condition. Gene sets from Gene Ontology biological process and cellular components were utilized to determine affected pathways.

### Cell immunofluorescence imaging

Following treatments as indicated, 100% confluent LECs were fixed (4% formaldehyde, 10 min), permeabilized (0.1% Triton X-100/PBS, 10 min), and blocked (1% BSA/PBS, 60 min). Primary (overnight at 4°C) and fluorescent-labeled secondary antibodies (1 h at room temperature) were added (supplemental Table S1), each followed by PBS washes at room temperature. Cells were then mounted on a coverslip using ProLong™ Gold Anti-fade mounting medium with DAPI (ThermoFisher Scientific) for microscope visualization. To determine rearrangement of the cytoskeleton, phalloidin staining (1:400, AlexaFluor647-Phalloidin, ThermoFisher Scientific) was performed concomitantly with the junction staining following the manufacturer's instructions. Quantification of junctional thickness was assessed in a blinded manner to prevent experimenter bias. ImageJ was used to manually draw lines perpendicularly to VE-cadherin between adjacent cells in each image, 10 fields per replicate. The width of each junction was recorded. The majority (>75%) of junctions in control monolayers were between 7-10 μm wide. Junctions thicker than 7 μm were classified as “thick” and that less than 7 μm in width were classified as “thin”, and percentages of thin and thick junctions were derived from these measurements. Stacks of the images were analyzed in Fiji by the JACoP plugin for colocalization analysis. Images were converted to 8-bit and background subtracted using a rolling-ball algorithm (r = 50). The Mander's coefficients of VE-cadherin and phalloidin channels were calculated based on the same threshold for the experiment, and data reported as the fraction of VE-cadherin-stained pixels overlapping with phalloidin stain. Two-color intensity profiles were generated in Fiji by drawing straight lines in the overlay images and then plotted in Prism.

### Lipid uptake assay

Serum-starved (12 h) LECs were treated as in the *“Cell treatment”* section, 2 μM BODIPY-C16 was added to the cell culture medium during the last 3 h of the cell stimulation. Cells were then washed three times with PBS, fixed with 4% paraformaldehyde for 10 min, and washed (3 × 5 min) with PBS before mounting in Prolong Antifade Mounting media (ThermoFisher). Slides were imaged using a Leica SP8 TCS STED 3X Point Scanning Confocal, equipped with a 63X oil objective. Five images of non-overlapping fields were taken randomly, and Image J software was used to determine the area in each view field.

### Transendothelial electrical resistance (TEER)

The integrity of the monolayer was assessed by measuring changes in TEER using an electrical cell-substrate impedance sensing ECIS (Applied Biophysics) which was housed in a standard tissue culture incubator maintained at 37°C and 5% CO_2_. Cells (5 × 10^4^) were seeded using complete EGM-2MV media into plates with gold-coated microelectrodes (Applied Biophysics, cat. #8W10E+) and inspected under the phase contrast microscope to verify that the monolayer was complete and overtly normal. On the day of the experiment, cells were starved (0.5% FBS) before placing the chamber in the TEER instrument. Impedance was monitored stabilized (plateau) before adding the same volume of either media or insulin (100 nM), or CMA (20 μM), or CMA (20 μM) and insulin (100 nM) together. Lipopolysaccharide (LPS, 50 ng/ml), which impairs the integrity of the monolayer (31), was used as a positive control. The electrical current passing through the cell monolayers was measured independently in each well and continuously in real time for 18 h. TEER measurements are presented as resistance (Ohm) measured in each well over time. All treatments were tested in duplicates, and experiments were repeated three times.

### Western blotting

Cells were lysed in a cold 1X Cell Lysis Buffer (Cell Signaling) containing cOmplete Mini proteinase inhibitors and PhosphoStop. Lysates were cleared (12,500 × g, 15 min) and assayed for protein concentration by Pierce™ BCA Protein Assay (ThermoFisher Scientific). Proteins (15 μg) were separated on 4%–12% gradient gels and transferred to nitrocellulose membranes (Bio-Rad), blocked (Li-COR Biosciences) 1 h at room temperature before incubation with primary antibodies (supplemental Table S1) overnight at 4 °C. Infrared dye-labeled secondary antibodies (supplemental Table S1) were added for 1 h at room temperature. Protein signals were detected using the Li-Cor Odyssey Infrared (Li-COR Biosciences) and quantified using Image Studio Lite v5.2 Software.

### Co-immunoprecipitation

Following insulin treatment, cells were washed with PBS and incubated for 30 min at 37°C with a cross-linking solution containing 0.5 mM of dithiobis(succinimidyl Propionate) (DSP, Thermo Fisher Scientific). The reaction was stopped by incubating with 20 mM Tris-HCl (pH 7.5) in PBS for 15 min at RT, followed by washes with PBS. Lysates were collected, assayed for protein concentration, and pre-cleared with 30 μl of protein G-linked agarose beads (Sigma) for 1 h at 4°C, under agitation. Pre-cleared lysates (500 μg) were incubated overnight at 4°C with either 2 μg of mouse anti-CD63 antibody or mouse control IgG (Santa Cruz Biotechnology), along with protein G-coupled agarose beads (50 μl). After incubation, the beads were pelleted (2000 rpm, 2 min, 4°C) and washed three times. Equal volumes of eluted proteins (30 μl) and 10 μg of pre-cleared lysate (input) were then separated by Western blot and probed for CD63 and integrin β1 primary antibodies overnight at 4°C, followed by horseradish peroxidase (HRP)-conjugated secondary antibodies for 1 h at room temperature (supplemental Table S1). Chemiluminescence detection was performed using a ChemiDoc MP Imaging System (Bio-Rad Laboratories).

### Proximity ligation assay (PLA)

PLA protocol was performed following the manufacturer's instructions (Sigma). Briefly, cells (∼13 × 10^5^) were seeded into poly-lysine-coated coverslips. After 48 h of CD36 silencing by siRNA, cells were starved for 1 h and then treated with media alone or supplemented with insulin (100 mM, 6 h). Cells were then fixed (4% formaldehyde, 10 min), permeabilized (0.5% Triton X-100/PBS, 10 min), blocked, and incubated with primary antibodies rabbit anti-human CD63 and mouse anti-human integrinβ1 (supplemental Table S1) overnight at 4°C. Anti-mouse and anti-rabbit probes were added the next day, followed by ligation and amplification steps. Coverslips were mounted on slides using ProLong™ Gold Anti-fade mounting medium with DAPI (ThermoFisher Scientific) and the PLA signal was detected as Texas Red® signal using a Leica CTR6 LED microscope. The signal was quantified as a mean intensity of fluorescence/cell/treatment, and the background was subtracted as the mean intensity of fluorescence of five different empty areas for each field using ImageJ software as previously described (32).

### Statistical analysis

GraphPad Prism software (version 8.4.3) was used for all statistical analyses and to plot quantitative data. All data shown are means ± standard error mean (SEM). Statistical significance was evaluated using a non-paired two-sided Student's *t* test with a *P* value of ≤0.05 indicating significant differences. One-way ANOVA with Sidak's *post hoc* test was used for multiple comparisons.

## Results

### Global identification of palmitoylated proteins in LECs and the effect of insulin

**Fig. 1:**
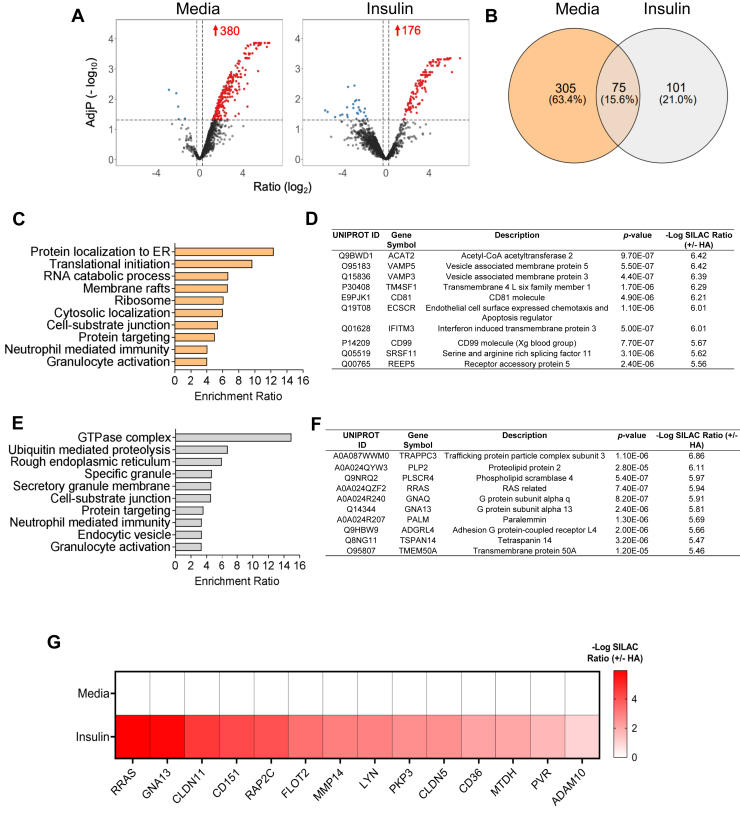
Palmitoylation proteomics in LECs. A: Volcano plots and (B) Venn diagram of palmitoylated targets in media (Media, left panel) and insulin (right panel) treated LECs. C, D: Pathway analysis and list of the of the 10 most upregulated target in LECs at baseline or (E, F) following 6 h insulin treatment. G: Heatmap of media and insulin-induced palmitoylated proteins with function in LEC integrity or junctional remodeling. Three matched groups of control and insulin treated cell preps were analyzed and normalized fold changes with *P* values were generated for individual proteins. Adjustments of *P*-values for multiple comparisons were used with Benjamini-Hochberg (BH) correction.

Since targets of lymphatic palmitoylation are unknown, we performed an unbiased quantitative proteomic profile of palmitoylated proteins in human dermal lymphatic endothelial cells (LECs) under unstimulated conditions (baseline) and in the presence of insulin to identify putative targets of insulin-regulated palmitoylation. The median half-life of mammalian proteins is estimated at ∼10 h (33), thus it is unlikely that steady-state protein levels would be affected within 6 h of insulin treatment. Following treatments, cells were first incubated with N-ethylmaleimide (NEM) to modify free thiols, to prevent their subsequent biotinylation, and then with hydroxylamine (HA^+^), which cleaves the thioester bond between palmitate and cysteines, leaving cysteines susceptible to biotin labeling and subsequent pulldown. As a control for the HA step, we generated a minus HA condition, so each sample was HA + or HA−. Unlabeled peptides were analyzed using trapped ion mobility time-of-flight mass spectrometry (supplemental Fig. S1) as described in Methods. Three matched groups of media or insulin-treated cell preps were analyzed, and normalized fold changes with *P*-values were generated for individual proteins. Of more than 3,300 identified proteins, 380 proteins were palmitoylated in unstimulated conditions (*P*-values ≤0.05 and ≥ 2 peptides) (Fig. 1A). These included proteins involved in vesicular or membrane trafficking, translation initiation, and membrane rafts, grouped based on functional characteristics (Fig. 1C, D; full list in supplemental Table S2). Insulin stimulation enriched the palmitoylation of 176 proteins (fold ≥1.2 and ≥ 2 identifying peptides), 75 of these were shared with unstimulated conditions and included proteins involved in GTPase signaling, ubiquitination, and junction anchoring (Fig. 1E, F and supplemental Table S3).

**Fig. 2:**
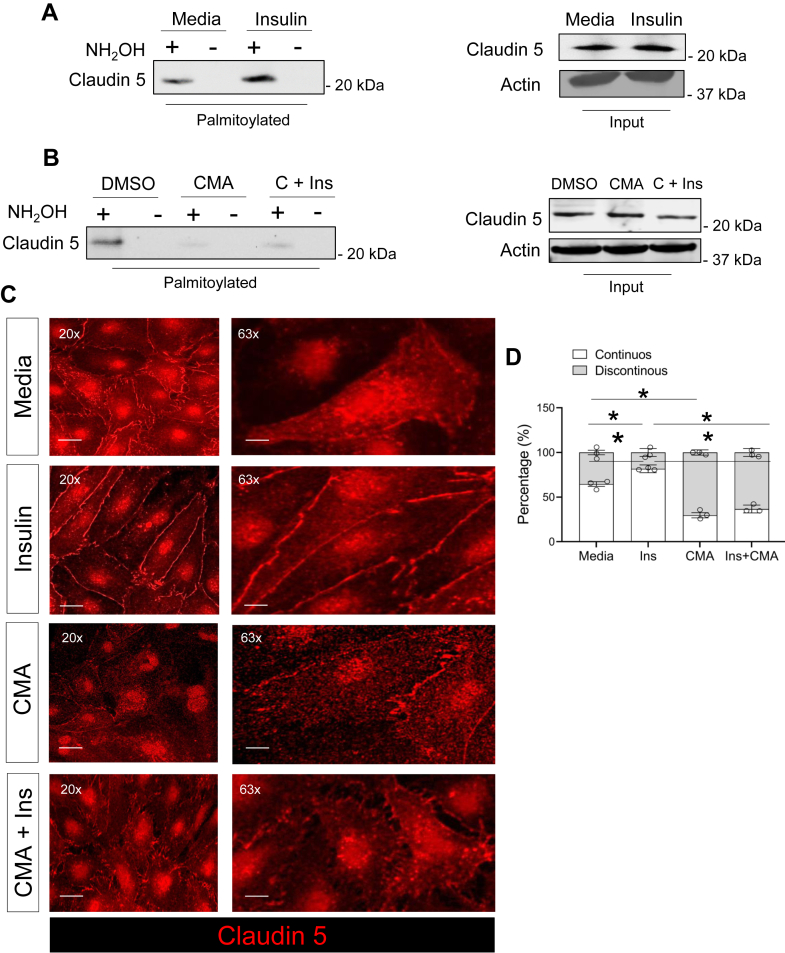
Insulin induces palmitoylation of tight junction claudin 5. A, B: Western blot analysis of palmitoylated claudin 5 by acyl-RAC assays with or without insulin treatment (left panel), and corresponding inputs (right panels). Cyano-myracrylamide at 20 μM (CMA) was used as a control for palmitoylation inhibition; C + Ins: CMA in combination with insulin. CMA is reconstituted in Dimethyl sulfoxide (DMSO), used to reconstitute CMA, is a control. C: Representative immunofluorescence staining and of claudin 5 remodeling in LECs treated with control media, insulin, CMA or CMA + insulin, respectively, depicting junctional remodeling of claudin 5. DAPI is a nuclear staining. Cells were treated with insulin at 100 nM for 6 h in the presence or absence of CMA at 20 μM throughout the course of treatment. D: Morphometric analysis of continuous versus discontinuous claudin 5 junctional staining at the plasma membrane obtained using ImageJ. Data (n = 3/group) are means ± SEM. ∗*P* < 0.05 by one-way ANOVA with Sidak's post hoc test for multiple comparisons.

Insulin signaling has been recently shown to regulate lymphatic endothelial integrity in vitro and in vivo (21, 34) but mechanisms remain largely unknown. Interestingly, in insulin condition, our proteomic screen showed enrichment in palmitoylation of proteins involved in endothelial barrier integrity (i.e., claudin 5, claudin 11, CD36, plakophilin 3, RAP2C, and flotillin 2) and in transendothelial migration (i.e., CD155, CD151, and MTDH: methadherin), a process that favors the movement of leukocytes across the endothelium and requires the remodeling of cell-cell junctions (35) (Fig. 1G). Therefore, our subsequent target validation focused on junctional anchoring proteins with established functions in LEC integrity. Among these proteins, insulin-induced palmitoylation of the tight junction claudin 5 which co-localizes at the plasma membrane with the cell-cell junction vascular endothelial (VE)-cadherin (supplemental Fig. S2A, B), a gatekeeper of lymphatic integrity (36). AcylRac assay followed by western blotting confirmed higher palmitoylation levels of claudin 5 in insulin-treated LECs as compared to unstimulated cells, while palmitoylation inhibitor CMA reversed this effect (Fig. 2A, B). We next employed confocal microscopy to determine whether insulin and/or inhibition of palmitoylation by CMA would impact claudin 5 morphological remodeling at the plasma membrane since the linear versus discontinuous junctional morphology regulates LEC integrity (36). Immunofluorescence analysis showed that insulin treatment induced a more continuous linear claudin 5 staining at the plasma membrane compared to the untreated condition which was reversed by treatment with CMA (Fig. 2C, D). Of note, insulin treatment induced LEC swelling, which may occur during the activation of the anabolic pathway (37).

**Fig. 3:**
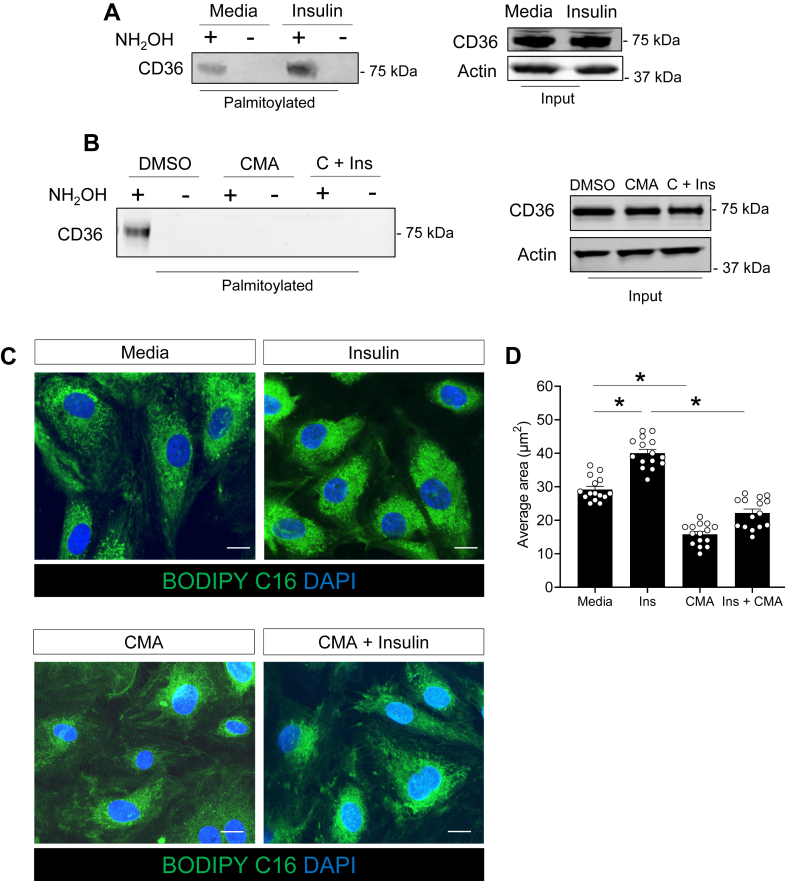
Insulin induces palmitoylation of CD36 in cultured LECs. A, B: Western blot analysis of palmitoylated CD36 by acyl-RAC assays with or without insulin treatment (left panel), and corresponding inputs (right panels). Cyano-myracrylamide at 20 μM (CMA) was used as a control for palmitoylation inhibition; C + Ins: CMA in combination with insulin. CMA is reconstituted in Dimethyl sulfoxide (DMSO), used to reconstitute CMA, is a control. C, D: Representative images and quantification of BODIPY-C16 fluorescence in LECs starved 1 h and treated as indicated. Five images of non-overlapping fields for each site in the same sample were taken randomly, and the fluorescence area was determined by Image J software. Data (n = 3/group) are means ± SEM. ∗*P* < 0.05 by one-way ANOVA with Sidak's post hoc test for multiple comparisons.

Our proteomic screening also revealed a significant enrichment in the palmitoylation of CD36, a long-chain fatty acid transporter, following insulin treatment as compared to media alone. This finding was confirmed by AcylRac and Western blot analysis (Fig. 3A). Notably, CMA treatment abolished insulin-induced palmitoylation (Fig. 3B). As CD36 palmitoylation is critical for its lipid uptake function (38), we next assessed lipid uptake in LECs stimulated with insulin and/or CMA. Insulin significantly enhanced the uptake of fluorescently labeled lipids (BODIPY-C16) compared to media alone (Fig. 3C). In contrast, CMA not only reduced basal lipid uptake in LECs, consistent with previous observations in CMA-treated pre-adipocytes (23) but also abolished the insulin-stimulated increase in lipid uptake (Fig. 3C).

### Role of protein palmitoylation in LECs

**Fig. 4:**
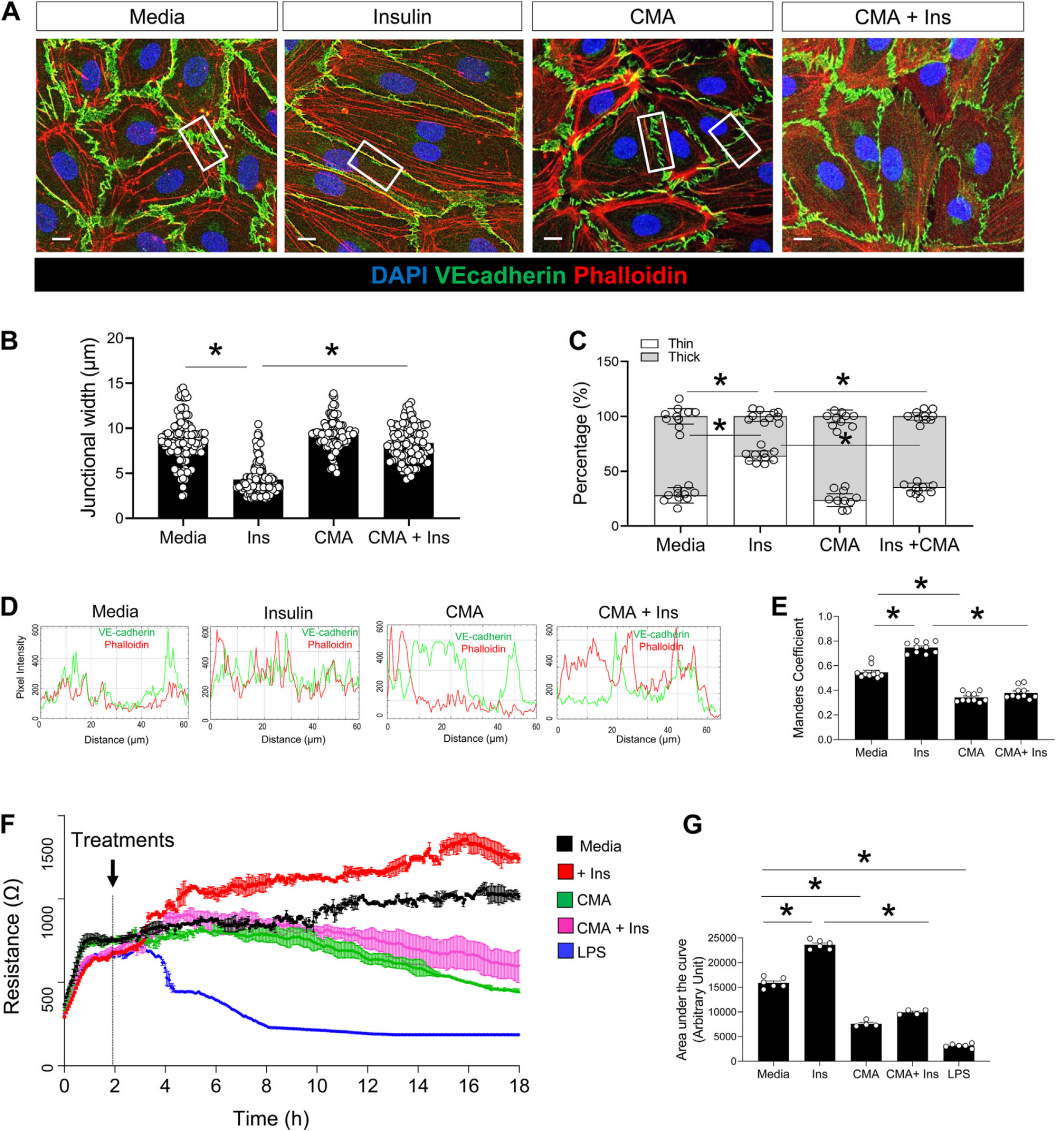
Insulin regulates LEC integrity by promoting straighter VE-cadherin junctions. A: Representative immunofluorescence images showing that insulin (100 nM) induces straighter VE-cadherin junctions (white squares) in LECs as compared to media alone. Cyano-myracrylamide (CMA, 20 μM), alone or in combination with insulin treatment, reverses insulin-mediated VE-cadherin linearization. Scale bar: 20 μm. B, C: Quantification of VE-cadherin junction width as an indicator of junction linearity. D: Stacks of images were analyzed in Fiji by the JACoP plugin for colocalization analysis (white box). Data (n = 10 cells in 10 images/group) are means ± SEM. ∗*P* < 0.05 by one-way ANOVA with Sidak's post hoc test for multiple comparisons. E: Quantification of VE-cadherin co-localization with phalloidin (Mander's coefficient). F: TEER measurements in a fully confluent LEC monolayer treated with media only, insulin (100 nM), CMA (20 μM), insulin and CMA, or LPS (50 ng/ml). G: Quantification of the area under the curve for each treatment. Data (n = 4–6/group) are means ± SEM. ∗*P* < 0.05 by One-way ANOVA with Sidak's post hoc test for multiple comparisons.

Since several of the above-palmitoylated proteins are involved in LEC integrity, we next addressed the potential role of global palmitoylation in lymphatic VE-cadherin junctional remodeling. Using confocal fluorescence microscopy, we observed that insulin treatment induced straighter VE-cadherin–lined junctions and increased actin filament (phalloidin) anchoring to VE-cadherin perpendicularly with colocalization evident by the yellow merge between green VE-cadherin and red phalloidin (Fig. 4A). Compared to untreated conditions, quantification showed that insulin treatment associates with a significant: *i)* reduction in junctional width, *ii)* increased percentage of thin VE-cadherin junctions (Fig. 4B), and *iii)* higher VE-cadherin-actin colocalization (Fig. 4C) with a 1.5 increase in Mander's overlap coefficient (Fig. 4D, E). Inhibition of palmitoylation by CMA, in media or together with insulin treatment, blunted insulin-induced linearization of VE-cadherin, increased junctional width and thickness (*P* < 0.05) (Fig. 4B, C), and reduced VE-cadherin-actin co-localization (Mander's coefficient, *P* < 0.05) (Fig. 4D, E). Similarly, TEER measurements conducted in fully confluent LEC monolayers showed a tighter monolayer in the insulin-treated condition as compared to media only (*P* < 0.05 from time point ∼5 h up to 18 h) (Fig. 4F, G). TEER measurements were significantly reduced in CMA (as compared to untreated condition *P* < 0.05 from time point ∼10 h up to 18 h) and CMA + insulin treatment (as compared to insulin condition *P* < 0.05 from time point ∼6 h up to 18 h). As expected, LPS caused a rapid decline in TEER (Fig. 4F, G).

### Role of CD36 in protein palmitoylation in LECs

**Fig. 5:**
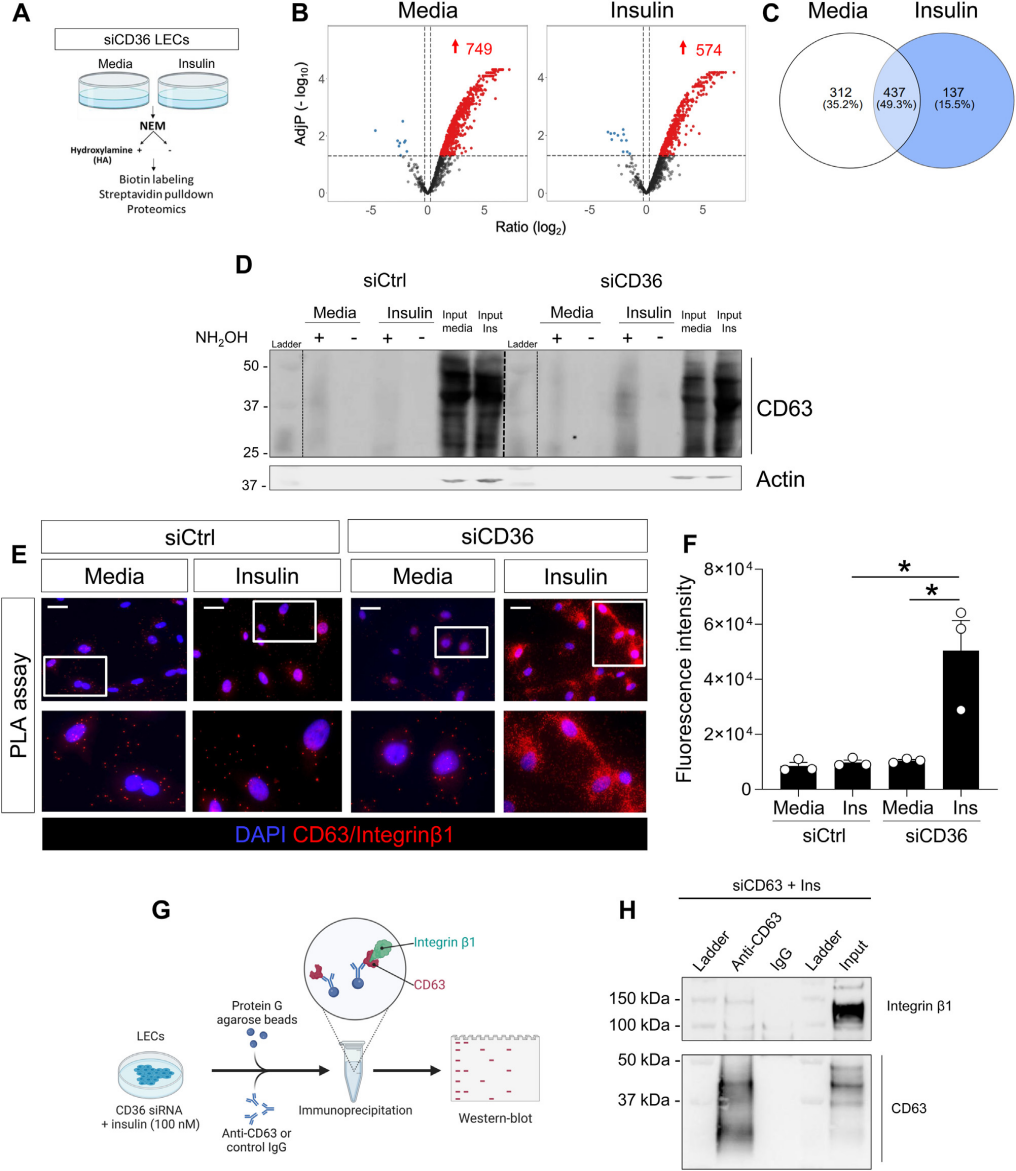
CD36 regulates LEC palmitoylation profile in LECs. A: Strategy for the isolation of the palmitoylated proteins. B: Volcano plots and (C) Venn diagram of palmitoylated targets in media or insulin treated siCD36 LECs. Three matched groups of media or insulin treated cell preparations were analyzed and normalized fold changes were generated with *P* values for individual proteins. D: Western blot analysis of pulldown CD63 palmitoylated by acyl-RAC assays in siCtrl and siCD36 LEC following media or insulin treatment. Lysates are from three independent experiments that were pulled together. Actin was used as a loading control for the input. E: In situ proximity ligation assay (PLA) detection of CD63 and integrin β1 vicinity (red fluorescence) in siCtrl or siCD36 LECs following media or insulin treatment (100 nM, 6 h) and (F) signal quantification. Signal (n = 3/10 cells/treatment) was quantified as a mean fluorescence intensity and background was subtracted as the mean fluorescence intensity of five different empty areas for each field using ImageJ software. Data (n = 3/group) are means ± SEM. ∗*P* < 0.05 by 2-tailed Student *t* test. G: co-immunoprecipitation strategy of CD63 and integrin β1 in insulin-treated siCD36 LECs and (H) resulting immunoblotting.

Palmitoylation can be fueled by the uptake of palmitate, imported across the plasma membrane via CD36 (39). Alternatively, palmitate can be synthesized endogenously from non-FA precursors by FA synthase (FASN). To determine whether CD36 is a rate-limiting substrate for palmitoylation cycling, we silenced CD36 expression in LECs using small interfering RNA (siCD36) before subjecting cells to media or insulin treatment (100 nM, 6 h) (Fig. 5A) and proteomic analysis. CD36 silencing, confirmed by western blotting, did not affect FASN expression (supplemental Fig. S3). CD36 silencing was associated with a 1.8-fold increase in palmitoylated targets in media (749 palmitoylated proteins in siCD36 vs. 380 in siCtrl LECs), and with an ∼ 3-fold increase in targets in insulin-treated LECs (574 palmitoylated proteins in insulin-treated siCD36 cells vs. 176 targets in WT LECs) (Fig. 5B, C, supplemental Fig. S4, and supplemental Table S3 and S4). As compared to insulin-stimulated siCtrl LECs, insulin stimulation in siCD36 LECs increased palmitoylation of proteins involved in mitochondrial respiration (*P* < 2.2e-16, FDR < 2.2e-16), protein localization to the endoplasmic reticulum (*P* < 3.34E-12; FDR<7.11E-10), and neutrophil-mediated immunity (*P* < 2.2e-16, FDR < 2.2e-16) (supplemental Table S5), while the palmitoylation of 90 proteins including R-Ras and claudin 11 was lost (supplemental Table S6).

### Insulin induces palmitoylation of tetraspanin CD63 and its interaction with integrin β1 in LECs devoid of CD36

Among the targets identified by our proteomic screens, insulin stimulation increased the palmitoylation of the tetraspanin CD63 in LECs devoid of CD36 compared to insulin-treated siCtrl LECs (supplemental Tables S4 and S5), which was validated by AcylRAc pulldown and Western blotting (Fig. 5D). In contrast, levels of CD63 palmitoylation did not differ in either unstimulated (media) siCtrl or siCD36 LECs (Fig. 5D). Of note, CD63 smearing pattern is due to a presence of glycosylation sites on this protein as previously reported (40). CD63 is a member of the transmembrane-4 glycoprotein superfamily that clusters with other tetraspanin family members (i.e., CD53) and with integrins (41) (i.e., integrin β1) to regulate membrane protein trafficking, leukocyte recruitment, and adhesion processes (32). In addition, there is evidence that signaling pathways initiated by growth factors synergize functionally with those triggered by integrins (42) to regulate interaction between the cell surface with the extracellular matrix. Based on these findings, we next sought to determine whether insulin signaling promotes CD63 interaction with integrin β1. Proximity ligation assay (PLA) showed a significant increase in CD63–integrin β1 interactions upon insulin stimulation in siCD36-treated cells compared to their media counterparts (Fig. 5E, F). In contrast, no differences in PLA signals were observed in control (siCtrl) cells under media or insulin-stimulated conditions. The interaction between CD63 and integrin β1 in insulin-treated siCD36 LECs was further confirmed by co-immunoprecipitation, which was not observed in the IgG control condition (Fig. 5G, H). Together, these findings indicate that CD63 palmitoylation is enhanced by insulin specifically in the absence of CD36 and suggest that this modification may facilitate CD63 association with integrin β1.

## Discussion

Our study is the first to investigate the role of palmitoylation—a reversible lipid-based post-translational modification—in LEC function. Using quantitative proteomics, we generated comprehensive palmitoylation profiles in human LECs across different conditions, providing novel insights into their regulatory role. In the unstimulated state, palmitoylation was predominantly enriched in proteins associated with vesicular and membrane trafficking, and translation initiation, reflecting its fundamental role in maintaining cellular homeostasis. Given the established link between insulin signaling, lipid metabolism, and LEC function, we further explored how insulin influences global palmitoylation. Upon insulin stimulation, we identified an enrichment of palmitoylation in proteins critical for maintaining LEC integrity, including junctional proteins, small GTPases involved in cytoskeletal dynamics, and ubiquitination enzymes that regulate protein turnover. Since cellular uptake of palmitate, the substrate for palmitoylation, is primarily mediated by the long-chain fatty acid transporter CD36, we investigated its role in regulating palmitoylation in LECs. Silencing CD36 led to a twofold increase in the palmitoylation of proteins involved in inflammation and immune cell activation indicating a critical regulatory role of CD36 in maintaining the specificity of palmitoylation under physiological conditions. Collectively, our findings establish palmitoylation as a key regulatory mechanism in LEC barrier function and highlight its interplay with insulin signaling and lipid metabolism.

The metabolic syndrome, characterized by an abnormal lipid profile and insulin resistance, is associated with impaired lymphatic function in peripheral tissues (22, 34). An abnormal lipid profile is detrimental to LEC survival (43, 44) and monolayer integrity. This is consistent with findings in rodent models of hypercholesterolemia (45, 46), dyslipidemia (47, 48), and diet-induced obesity (9). Together, these data support a link between impaired lipid metabolism and lymphatic vessel dysfunction. Compared to blood endothelial cells, LECs exhibit higher rates of FA uptake and fatty acid β-oxidation, facilitated by elevated expression of the FA transporter CD36 and of proteins mediating FA import in the mitochondria (3). Fatty acid β-oxidation is critical for LEC differentiation, as it generates acetyl-CoA, a key substrate for histone acetylation at lymphangiogenic genes, driving transcriptional programs essential for lymphatic development and function (5). Beyond its role in epigenetic regulation, acetyl-CoA can also be converted to palmitoyl-CoA, which supports palmitoylation (12). Reversible palmitoylation modulates spatiotemporal control of protein function and activity as well as protein–protein, and membrane–protein interactions (49). Dysregulated palmitoylation has been reported in a plethora of pathologies ranging from metabolic syndrome, neurological diseases, cancer, pathogen infections, or histamine-mediated endothelial inflammation (11), and is indicative of disrupted lipid homeostasis. Thus, elucidating the targets of palmitoylation in lymphatic vessels is crucial for understanding its regulatory role in lymphatic function in health and disease.

While palmitoylation has been investigated in several cell types (11) including venous endothelial cells (16), its role in LECs remains unexplored. Our proteomic analysis showed that, under unstimulated conditions, approximately 10% of total LEC proteins are palmitoylated, most of them being involved in vesicular or membrane trafficking, and in localization to ER. Insulin, instead, induced the palmitoylation of proteins involved in integrity such as small GTPases, which enhance lymphatic junction stability and permeability control by modulating cytoskeleton dynamics and GDP-GTP exchange (36). Additionally, insulin increased the palmitoylation of key adherens and tight junction components. Notably, we validated the palmitoylation of claudin 5 using AcylRAc assays and western blotting. Immunofluorescence studies further revealed that insulin treatment coincided with a more linearized remodeling of claudin 5 at the plasma membrane. This observation is significant, as claudin 5 exhibits overlapping distribution with VE-cadherin, a key gatekeeper of the lymphatic barrier. Tight junctions have been reported to be particularly abundant in cerebrovascular endothelial cells and necessary for the integrity and maintenance of the blood–brain barrier (50). Additionally, claudin 5^*+/−*^ mice exposed to ultraviolet B irradiation develop edema due to lymphatic vessel leakage (51), suggesting that claudin-5 is crucial for the maintenance of collecting lymphatic vessel integrity in most anatomical regions of the body. Consistent with this, our study showed that trans-endothelial electrical resistance (TEER) measurements in a fully confluent LEC monolayer showed increased barrier tightness following insulin treatment, whereas inhibition of palmitoylation by CMA reversed this effect. These elements suggest an important role for protein palmitoylation in the regulation of lymphatic vessel permeability/integrity.

Our studies also showed that insulin-induced palmitoylation of CD36, a transmembrane receptor with established functions in long-chain FA uptake and lipid metabolism (52, 53). This finding is in line with previous reports showing CD36 palmitoylation in adipocytes (38, 54), cardiomyocytes (55), melanoma cells (56), macrophages (57), and venous endothelial cells (16) in response to insulin and/or lipid stimulation. CD36 is most likely palmitoylated at the endoplasmic reticulum (ER), where this modification is essential for its maturation and proper functioning (58). In addition, palmitoylation of CD36 promotes its membrane localization (59), and inhibition of this modification leads to its accumulation in the ER, reducing the efficiency of lipid uptake (56). Notably, our study showed that the insulin stimulation increased lipid droplet formation in LECs and that inhibition of palmitoylation was associated with reduced formation of lipid droplets in line with a previous report in CMA-treated pre-adipocytes (23). Further studies are needed to determine if insulin and/or inhibition of palmitoylation could regulate CD36 trafficking and localization.

Since CD36 is a major route for the uptake of circulating palmitate into cells, we sought to determine whether CD36 silencing in LECs would affect the global palmitoylation profile. LECs devoid of CD36 presented doubled rates of palmitoylation, targeting proteins involved in inflammation, leukocyte recruitment, and neutrophil degranulation. Of note, palmitoylation of tetraspanin CD63 was significantly increased in insulin-treated siCD36 cells as compared to siCtrl LECs. Tetraspanins and many of their partner proteins have palmitoylation sites to support interactions with other tetraspanins and integrins for the propagation of distinct signaling pathways (60). We found that in siCD36 LECs, insulin increased CD63 interaction with binding partner integrin β1, which previous studies have shown to modulate recruitment and activation of neutrophils (61). These results are in line with our prior work showing neutrophil activation and infiltration in mice lacking CD36 globally or with a tissue-specific deletion restricted to the blood and lymphatic endothelium (62). Our current study also found that CD36 silencing increased palmitoylation of proteins involved in glycolysis and gluconeogenesis, which may have contributed to the increased glycolytic levels and expression of genes encoding for glycolytic enzymes previously reported by our group in cultured LECs and in gut lymphatics (6). We previously showed that CD36 silencing in LECs reduced VEGF-C mediated phosphorylation of VEGFR-2/AKT and impaired LEC migration, tube formation, and monolayer integrity. In vivo, mice with inducible deletion of CD36 in LECs manifested compromised integrity of the collectors, spillover of lymph in the mesentery, and spontaneous obesity (6). Our current study adds a new mechanism for CD36 effects in LECs as CD36 silencing causes a loss of palmitoylation by members of the RAS family, important for lymphangiogenesis (63), and by claudin 11, highly enriched at valves in lymphatic collectors (64). How palmitoylation contributes to RAS or claudin 11 functions in lymphatics is not known and warrants further investigation. Overall, our findings suggest that CD36 may regulate lymphatic function through multiple mechanisms, highlighting its potential as a key regulator of lymphatic biology.

In conclusion, our studies show that protein palmitoylation plays a critical role in LEC barrier function. The insights gained from our LEC palmitoylation profile, in unstimulated and insulin-treated cells, with or without CD36, provide a foundation for future research aimed at exploring the role of lipid metabolism and insulin signaling in lymphatic vessels during homeostasis and the metabolic syndrome, characterized by altered lipid profile and insulin resistance.

## Data availability

The authors confirm that the data supporting the palmitoylation proteomics findings of this study are available within the article and its supplementary materials. The data that support additional findings of this study are available on request from the corresponding author.

## Supplemental data

This article contains supplemental data.

## Conflicts of interest

The authors declare that they have no conflicts of interest with the contents of this article.

## Supplementary data

Suppl Tables 1-6
