# Supplementary material for: Insulin regulates lymphatic endothelial integrity via palmitoylation
Source: J Lipid Res. 2025 Mar 11;66(4):100775. doi: 10.1016/j.jlr.2025.100775 (PMC12002826; doi:10.1016/j.jlr.2025.100775)
Supplement: Suppl Tables 1-6 [file mmc1.pdf]

Supplementary Table 1. List of primary and secondary antibodies.

Antibody	Species	Identifier	Cat. No.	Clone	Dilution
**Immunofluorescence**					
CD36	Goat anti-human	R&D Systems	AF1955	n/a	1:100
Claudin 5	Rabbit anti-human	Abcam	Ab15106	n/a	1:250
VE-cadherin	Mouse anti-human	Santa Cruz Biotechnology	Sc-9989	F-8	1:100
CD63	Rabbit anti-human	Invitrogen	PA5-92370	n/a	1:100
Integrin β1	Mouse anti-human	Invitrogen	14-0299-82	Ts2/16	1:100
Secondary	Donkey anti-Rabbi AF488	Invitrogen	A21206	n/a	1:250
Secondary	Donkey anti-Mouse AF594	Invitrogen	A11005	n/a	1:250
Secondary	Donkey anti-Mouse AF568	Invitrogen	A10037	n/a	1:250
Secondary	Donkey anti-Goat AF647	Invitrogen	A32849	n/a	1:250
**Western Blotting**					
CD36	Goat anti-human	R&D Systems	AF1955	n/a	1:1000
Claudin 5	Mouse anti-human	Invitrogen	35-2500	4C3C2	1:1000
FASN	Rabbit anti-human	Abcam	ab128856	EPR7465	1:1000
CD63	Rabbit anti-human	Invitrogen	PA5-92370	n/a	1:500
Integrin β1	Rabbit anti-human	Abcam	ab52971	EP1041Y	1:2000
β-actin	Mouse anti-human	Cell Signaling	3700	8H10D10	1:10,000
Secondary	Donkey anti-mouse 800CW	LiCor	926-32212	n/a	1:10,000
Secondary	Donkey anti-goat 680RD	LiCor	926-68074	n/a	1:10,000
Secondary	Donkey anti-rabbit 680RD	LiCor	926-68073	n/a	1:10,000
Secondary	Donkey anti-rabbit 800CW	LiCor	926-32213	n/a	1:10,000
Secondary	Mouse anti-rabbit light chain specific-HRP	Jackson ImmunoResearch	211-032-171	n/a	1:10,000
Secondary		Jackson ImmunoResearch			
**Co-immunoprecipitation**					
CD63	Mouse anti-human	Santa Cruz Biotechnology	Sc-5275	n/a	n/a

Supplemental Table S2. Palmitoylated proteins in human LECs.

ENSEMBL ID	Gene Symbol	Description	UNIPROT	*p*-value	-Log Ratio (+/-HA)
ENSG00000120437	ACAT2	acetyl-CoA acetyltransferase 2	Q9BWD1	9.70E-07	6.42
ENSG00000168899	VAMP5	vesicle associated membrane protein 5	O95183	5.50E-07	6.42
ENSG00000049245	VAMP3	vesicle associated membrane protein 3	Q15836	4.40E-07	6.39
ENSG00000169908	TM4SF1	transmembrane 4 L six family member 1	P30408	1.70E-06	6.29
ENSG00000110651	CD81	CD81 molecule	E9PJK1	4.90E-06	6.21
ENSG00000249751	ECSCR	endothelial cell surface expressed chemotaxis and apoptosis regulator	Q19T08	1.10E-06	6.01
ENSG00000142089	IFITM3	interferon induced transmembrane protein 3	Q01628	5.00E-07	6.01
ENSG00000002586	CD99	CD99 molecule (Xg blood group)	P14209	7.70E-07	5.67
ENSG00000116754	SRSF11	serine and arginine rich splicing factor 11	Q05519	3.10E-06	5.62
ENSG00000129625	REEP5	receptor accessory protein 5	Q00765	2.40E-06	5.56
ENSG00000133818	RRAS2	RAS related 2	P62070	9.10E-07	5.56
ENSG00000134247	PTGFRN	prostaglandin F2 receptor inhibitor	Q9P2B2	1.60E-06	5.55
ENSG00000135404	CD63	CD63 molecule	A0A024RB05	3.10E-06	5.5
ENSG00000148175	STOM	stomatin	F8VSL7	1.20E-06	5.42
ENSG00000141526	SLC16A3	solute carrier family 16 member 3	A0A024R8U1	3.80E-06	5.41
ENSG00000102007	PLP2	proteolipid protein 2	A0A024QYW3	6.60E-05	5.4
ENSG00000059573	ALDH18A1	aldehyde dehydrogenase 18 family member A1	P54886	6.10E-06	5.25
ENSG00000010278	CD9	CD9 molecule	A6NNI4	5.60E-06	5.22
ENSG00000075239	ACAT1	acetyl-CoA acetyltransferase 1	A0A140VJX1	1.90E-06	5.21
ENSG00000076706	MCAM	melanoma cell adhesion molecule	A0A024R3I5	2.10E-06	5.19
ENSG00000078140	UBE2K	ubiquitin conjugating enzyme E2 K	P61086	1.50E-06	5.17
ENSG00000130725	UBE2M	ubiquitin conjugating enzyme E2 M	A0A024R4T4	2.30E-06	5.04
ENSG00000108848	LUC7L3	LUC7 like 3 pre-mRNA splicing factor	J3KPP4	3.30E-06	5.03
ENSG00000115310	RTN4	reticulon 4	Q9NQC3	2.40E-06	5.02
ENSG00000177889	UBE2N	ubiquitin conjugating enzyme E2 N	P61088	9.50E-06	4.87
ENSG00000139921	TMX1	thioredoxin related transmembrane protein 1	Q9H3N1	3.10E-06	4.7
ENSG00000136156	ITM2B	integral membrane protein 2B	Q9Y287	1.10E-05	4.64
ENSG00000143878	RHOB	ras homolog family member B	P62745	1.90E-05	4.56
ENSG00000143222	UFC1	ubiquitin-fold modifier conjugating enzyme 1	Q9Y3C8	1.30E-05	4.56
ENSG00000188313	PLSCR1	phospholipid scramblase 1	O15162	1.00E-05	4.54
ENSG00000103018	CYB5B	cytochrome b5 type B	J3KNF8	4.40E-06	4.49
ENSG00000026508	CD44	CD44 molecule (Indian blood group)	P16070	3.70E-06	4.42
ENSG00000243279	PRAF2	PRA1 domain family member 2	A0A024QZ22	1.90E-05	4.42
ENSG00000131037	EPS8L1	EPS8 like 1	Q8TE68	0.0038	4.41

ENSG00000197870	PRB3	proline rich protein BstNI subfamily 3	A0A0G2JNB4	3.00E-04	4.41
ENSG00000072274	TFRC	transferrin receptor	P02786	1.70E-05	4.34
ENSG00000112739	PRPF4B	pre-mRNA processing factor 4B	A0A024QZY5	1.90E-05	4.26
ENSG00000130985	UBA1	ubiquitin like modifier activating enzyme 1	A0A024R1A3	5.70E-06	4.26
ENSG00000185651	UBE2L3	ubiquitin conjugating enzyme E2 L3	P68036	1.10E-05	4.25
ENSG00000136026	CKAP4	cytoskeleton associated protein 4	A0A024RBH2	1.90E-05	4.24
ENSG00000198755	RPL10A	ribosomal protein L10a	P62906	5.90E-05	4.24
ENSG00000110090	CPT1A	carnitine palmitoyltransferase 1A	P50416	1.70E-05	4.23
ENSG00000072401	UBE2D1	ubiquitin conjugating enzyme E2 D1	A0A087WW00	1.10E-04	4.19
ENSG00000114353	GNAI2	G protein subunit alpha i2	B3KP24	9.40E-06	4.14
ENSG00000129353	SLC44A2	solute carrier family 44 member 2	A0A088QCU6	1.00E-05	4.13
ENSG00000006451	RALA	RAS like proto-oncogene A	P11233	1.00E-05	4.12
ENSG00000075142	SRI	sorcin	P30626	5.30E-04	4.11
ENSG00000135821	GLUL	glutamate-ammonia ligase	A8YXX4	0.027	4.04
ENSG00000198561	CTNND1	catenin delta 1	O60716	4.70E-05	3.97
ENSG00000182899	RPL35A	ribosomal protein L35a	P18077	1.50E-04	3.83
ENSG00000155380	SLC16A1	solute carrier family 16 member 1	A0A024R0H1	1.50E-04	3.82
ENSG00000059758	CDK17	cyclin dependent kinase 17	Q00537	8.90E-05	3.79
ENSG00000160221	GATD3B	glutamine amidotransferase class 1 domain containing 3	A0A0B4J2D5	1.50E-04	3.76
ENSG00000067057	PFKP	phosphofructokinase, platelet	Q01813	2.50E-05	3.7
ENSG00000143545	RAB13	RAB13, member RAS oncogene family	A0A087WWB9	5.80E-04	3.67
ENSG00000106263	EIF3B	eukaryotic translation initiation factor 3 subunit B	A0A024R821	6.00E-05	3.66
ENSG00000144746	ARL6IP5	ADP ribosylation factor like GTPase 6 interacting protein 5	A0A024R371	4.20E-04	3.64
ENSG00000131016	AKAP12	A-kinase anchoring protein 12	Q02952	3.20E-05	3.62
ENSG00000262814	MRPL12	mitochondrial ribosomal protein L12	P52815	6.20E-05	3.62
ENSG00000100201	DDX17	DEAD-box helicase 17	A0A1W2PQ51	3.10E-04	3.61
ENSG00000168703	WFDC12	WAP four-disulfide core domain 12	Q8WWY7	0.0073	3.61
ENSG00000167996	FTH1	ferritin heavy chain 1	A0A024R525	2.40E-05	3.6
ENSG00000198431	TXNRD1	thioredoxin reductase 1	Q16881	1.40E-04	3.54
ENSG00000265808	SEC22B	SEC22 homolog B, vesicle trafficking protein	O75396	1.30E-04	3.52
ENSG00000166479	TMX3	thioredoxin related transmembrane protein 3	Q96JJ7	3.00E-05	3.51
ENSG00000154473	BUB3	BUB3 mitotic checkpoint protein	A0A140VJF3	4.60E-05	3.49
ENSG00000141959	PFKL	phosphofructokinase, liver type	P17858	9.20E-04	3.49
ENSG00000164163	ABCE1	ATP binding cassette subfamily E member 1	P61221	1.00E-04	3.45
ENSG00000177674	AGTRAP	angiotensin II receptor associated protein	Q6RW13	7.00E-04	3.44
ENSG00000170889	RPS9	ribosomal protein S9	A0A024R4M0	9.20E-05	3.41
ENSG00000106636	YKT6	YKT6 v-SNARE homolog	A4D2J0	8.70E-05	3.4
ENSG00000142541	RPL13A	ribosomal protein L13a	P40429	2.40E-04	3.37

ENSG00000181163	NPM1	nucleophosmin 1	A0A140VJQ2	5.70E-04	3.33
ENSG00000108528	SLC25A11	solute carrier family 25 member 11	Q6IBH0	5.90E-04	3.33
ENSG00000127824	TUBA4A	tubulin alpha 4a	P68366	1.20E-04	3.33
ENSG00000172725	CORO1B	coronin 1B	A0A024R5K1	3.80E-05	3.32
ENSG00000159840	ZYX	zyxin	Q15942	1.20E-04	3.31
ENSG00000178597	PSAPL1	prosaposin like 1	Q6NUJ1	0.0097	3.3
ENSG00000112081	SRSF3	serine and arginine rich splicing factor 3	B2R6F3	5.80E-04	3.29
ENSG00000102886	GDPD3	glycerophosphodiester phosphodiesterase domain containing 3	Q7L5L3	0.0019	3.25
ENSG00000185627	PSMD13	proteasome 26S subunit, non-ATPase 13	Q9UNM6	1.00E-04	3.16
ENSG00000022267	FHL1	four and a half LIM domains 1	Q13642	2.10E-04	3.1
ENSG00000111737	RAB35	RAB35, member RAS oncogene family	Q15286	1.40E-04	3.08
ENSG00000089248	ERP29	endoplasmic reticulum protein 29	P30040	1.90E-04	3.07
ENSG00000111667	USP5	ubiquitin specific peptidase 5	A0A140VJZ1	8.00E-04	3.07
ENSG00000007168	PAFAH1B1	platelet activating factor acetylhydrolase 1b regulatory subunit 1	P43034	2.50E-04	3.06
ENSG00000091136	LAMB1	laminin subunit beta 1	P07942	1.70E-04	3.05
ENSG00000213625	LEPROT	leptin receptor overlapping transcript	A0A087X0N2	1.80E-04	3.04
ENSG00000109919	MTCH2	mitochondrial carrier 2	Q9Y6C9	1.60E-04	3.04
ENSG00000130309	COLGALT1	collagen beta(1-O)galactosyltransferase 1	Q8NBJ5	0.0013	3
ENSG00000115524	SF3B1	splicing factor 3b subunit 1	B4DGZ4	4.10E-04	2.98
ENSG00000103381	CPPED1	calcineurin like phosphoesterase domain containing 1	Q9BRF8	3.90E-04	2.97
ENSG00000103187	COTL1	coactosin like F-actin binding protein 1	Q14019	2.50E-04	2.96
ENSG00000186566	GPATCH8	G-patch domain containing 8	Q9UKJ3	2.80E-04	2.95
ENSG00000171564	FGB	fibrinogen beta chain	P02675	0.028	2.93
ENSG00000133318	RTN3	reticulon 3	O95197	4.10E-04	2.92
ENSG00000063177	RPL18	ribosomal protein L18	A0A024QZD1	7.10E-05	2.91
ENSG00000117448	AKR1A1	aldo-keto reductase family 1 member A1	P14550	1.20E-04	2.9
ENSG00000169710	FASN	fatty acid synthase	P49327	1.30E-04	2.9
ENSG00000138760	SCARB2	scavenger receptor class B member 2	Q14108	8.90E-05	2.9
ENSG00000105612	DNASE2	deoxyribonuclease 2, lysosomal	A0A024R7F4	6.20E-04	2.89
ENSG00000146963	LUC7L2	LUC7 like 2, pre-mRNA splicing factor	Q9Y383	1.40E-04	2.88
ENSG00000138119	MYOF	myoferlin	Q9NZM1	1.60E-04	2.88
ENSG00000125977	EIF2S2	eukaryotic translation initiation factor 2 subunit beta	P20042	2.20E-04	2.87
ENSG00000115380	EFEMP1	EGF containing fibulin extracellular matrix protein 1	A0A0S2Z4F1	1.30E-04	2.83
ENSG00000221914	PPP2R2A	protein phosphatase 2 regulatory subunit Balpha	P63151	1.10E-04	2.83
ENSG00000065978	YBX1	Y-box binding protein 1	P67809	2.30E-04	2.83
ENSG00000241553	ARPC4	actin related protein 2/3 complex subunit 4	P59998	7.00E-04	2.81

ENSG00000244687	UBE2V1	ubiquitin conjugating enzyme E2 V1	Q13404	0.0013	2.8
ENSG00000105953	OGDH	oxoglutarate dehydrogenase	A0A140VJQ5	1.00E-04	2.79
ENSG00000130255	RPL36	ribosomal protein L36	Q9Y3U8	4.40E-04	2.77
ENSG00000131469	RPL27	ribosomal protein L27	A0A024R1V4	9.40E-04	2.74
ENSG00000124570	SERPINB6	serpin family B member 6	A0A024QZX5	0.001	2.74
ENSG00000137486	ARRB1	arrestin beta 1	B7Z1Q3	3.90E-04	2.73
ENSG00000149218	ENDOD1	endonuclease domain containing 1	O94919	5.00E-04	2.72
ENSG00000198898	CAPZA2	capping actin protein of muscle Z-line subunit alpha 2	A4D0V4	5.60E-04	2.7
ENSG00000233276	GPX1	glutathione peroxidase 1	P07203	1.60E-04	2.7
ENSG00000138814	PPP3CA	protein phosphatase 3 catalytic subunit alpha	A0A0S2Z4C6	0.032	2.7
ENSG00000278637	H4C1	H4 clustered histone 1	B2R4R0	2.80E-04	2.69
ENSG00000104824	HNRNPL	heterogeneous nuclear ribonucleoprotein L	P14866	2.40E-04	2.68
ENSG00000147403	RPL10	ribosomal protein L10	P27635	3.00E-04	2.67
ENSG00000138029	HADHB	hydroxyacyl-CoA dehydrogenase trifunctional multienzyme complex subunit beta	P55084	9.40E-04	2.66
ENSG00000179091	CYC1	cytochrome c1	P08574	1.70E-04	2.63
ENSG00000105971	CAV2	caveolin 2	P51636	6.70E-04	2.62
ENSG00000182774	RPS17	ribosomal protein S17	P08708	0.002	2.62
ENSG00000101160	CTSZ	cathepsin Z	Q9UBR2	6.30E-04	2.61
ENSG00000138069	RAB1A	RAB1A, member RAS oncogene family	P62820	0.0014	2.59
ENSG00000165283	STOML2	stomatin like 2	Q9UJZ1	9.80E-04	2.58
ENSG00000072778	ACADVL	acyl-CoA dehydrogenase very long chain	P49748	2.50E-04	2.56
ENSG00000085491	SLC25A24	solute carrier family 25 member 24	Q6NUK1	0.013	2.56
ENSG00000079805	DNM2	dynamin 2	P50570	0.015	2.55
ENSG00000087460	GNAS	GNAS complex locus	A0A0S2Z3H8	2.80E-04	2.55
ENSG00000203879	GDI1	GDP dissociation inhibitor 1	A0A0S2Z3X8	4.70E-04	2.53
ENSG00000167792	NDUFV1	NADH:ubiquinone oxidoreductase core subunit V1	P49821	6.40E-04	2.5
ENSG00000124767	GLO1	glyoxalase I	Q04760	0.0019	2.47
ENSG00000004455	AK2	adenylate kinase 2	P54819	5.20E-04	2.46
ENSG00000184009	ACTG1	actin gamma 1	P63261	0.007	2.45
ENSG00000031698	SARS1	seryl-tRNA synthetase 1	Q5T5C7	3.60E-04	2.45
ENSG00000105379	ETFB	electron transfer flavoprotein subunit beta	P38117	3.80E-04	2.4
ENSG00000161057	PSMC2	proteasome 26S subunit, ATPase 2	B7Z571	4.90E-04	2.4
ENSG00000165799	RNASE7	ribonuclease A family member 7	Q9H1E1	0.026	2.4
ENSG00000113407	TARS1	threonyl-tRNA synthetase 1	P26639	0.0014	2.4
ENSG00000185825	BCAP31	B cell receptor associated protein 31	P51572	8.70E-04	2.38
ENSG00000165637	VDAC2	voltage dependent anion channel 2	P45880	5.70E-04	2.38
ENSG00000088832	FKBP1A	FKBP prolyl isomerase 1A	P62942	7.20E-04	2.37
ENSG00000071082	RPL31	ribosomal protein L31	P62899	0.009	2.36

ENSG00000117519	CNN3	calponin 3	Q15417	9.60E-04	2.34
ENSG00000198712	MT-CO2	mitochondrially encoded cytochrome c oxidase II	P00403	0.002	2.34
ENSG00000240386	LCE1F	late cornified envelope 1F	Q5T754	0.028	2.33
ENSG00000126432	PRDX5	peroxiredoxin 5	P30044	0.016	2.33
ENSG00000142676	RPL11	ribosomal protein L11	P62913	9.20E-04	2.32
ENSG00000109475	RPL34	ribosomal protein L34	A0A024RDH8	0.0014	2.32
ENSG00000139684	ESD	esterase D	A0A140VJJ2	0.0089	2.31
ENSG00000104388	RAB2A	RAB2A, member RAS oncogene family	P61019	8.70E-04	2.31
ENSG00000265681	RPL17	ribosomal protein L17	A0A024R261	0.0064	2.3
ENSG00000175793	SFN	stratifin	P31947	0.0042	2.29
ENSG00000088247	KHSRP	KH-type splicing regulatory protein	Q92945	9.00E-04	2.28
ENSG00000198363	ASPH	aspartate beta-hydroxylase	Q12797	8.90E-04	2.26
ENSG00000070831	CDC42	cell division cycle 42	A0A024RAE4	4.20E-04	2.23
ENSG00000172053	QARS1	glutaminyl-tRNA synthetase 1	B7Z840	6.20E-04	2.22
ENSG00000244038	DDOST	dolichyl-diphosphooligosaccharide--protein glycosyltransferase non-catalytic subunit	A0A024RAD5	0.0041	2.18
ENSG00000105974	CAV1	caveolin 1	A0A024R757	7.80E-04	2.17
ENSG00000092841	MYL6	myosin light chain 6	P60660	0.0033	2.17
ENSG00000161547	SRSF2	serine and arginine rich splicing factor 2	A0A024R8U5	0.0053	2.17
ENSG00000111275	ALDH2	aldehyde dehydrogenase 2 family member	P05091	0.0023	2.16
ENSG00000100030	MAPK1	mitogen-activated protein kinase 1	P28482	0.0014	2.16
ENSG00000138674	SEC31A	SEC31 homolog A, COPII coat complex component	O94979	0.0036	2.15
ENSG00000174444	RPL4	ribosomal protein L4	P36578	6.60E-04	2.13
ENSG00000108298	RPL19	ribosomal protein L19	P84098	0.0016	2.12
ENSG00000123416	TUBA1B	tubulin alpha 1b	P68363	0.023	2.11
ENSG00000197930	ERO1A	endoplasmic reticulum oxidoreductase 1 alpha	Q96HE7	0.0035	2.1
ENSG00000090863	GLG1	golgi glycoprotein 1	Q92896	0.0013	2.1
ENSG00000077147	TM9SF3	transmembrane 9 superfamily member 3	A0A024QYS2	9.80E-04	2.09
ENSG00000140740	UQCRC2	ubiquinol-cytochrome c reductase core protein 2	P22695	0.0016	2.08
ENSG00000135624	CCT7	chaperonin containing TCP1 subunit 7	Q99832	0.0038	2.05
ENSG00000064666	CNN2	calponin 2	B4DDF4	0.029	2.05
ENSG00000178035	IMPDH2	inosine monophosphate dehydrogenase 2	P12268	0.0028	2.05
ENSG00000165092	ALDH1A1	aldehyde dehydrogenase 1 family member A1	P00352	8.60E-04	2.04
ENSG00000023228	NDUFS1	NADH:ubiquinone oxidoreductase core subunit S1	P28331	9.50E-04	2.03
ENSG00000186468	RPS23	ribosomal protein S23	A8K517	0.014	2.03
ENSG00000142910	TINAGL1	tubulointerstitial nephritis antigen like 1	Q9GZM7	0.0011	2.03
ENSG00000075624	ACTB	actin beta	P60709	0.001	2.01

ENSG00000184743	ATL3	atlastin GTPase 3	B4DXC4	0.0011	2.01
ENSG00000196230	TUBB	tubulin beta class I	B4DY90	0.013	2.01
ENSG00000115541	HSPE1	heat shock protein family E (Hsp10) member 1	P61604	0.0019	2
ENSG00000197958	RPL12	ribosomal protein L12	P30050	0.001	2
ENSG00000128335	APOL2	apolipoprotein L2	A0A024R1M8	0.012	1.98
ENSG00000261150	EPPK1	epiplakin 1	A0A087X1U6	0.031	1.98
ENSG00000182199	SHMT2	serine hydroxymethyltransferase 2	P34897	0.0032	1.98
ENSG00000177688	SUMO4	small ubiquitin like modifier 4	Q6EEV6	0.026	1.97
ENSG00000133731	IMPA1	inositol monophosphatase 1	A0A140VJL8	0.0089	1.96
ENSG00000101150	TPD52L2	TPD52 like 2	O43399	0.032	1.96
ENSG00000143870	PDIA6	protein disulfide isomerase family A member 6	Q15084	0.0036	1.95
ENSG00000182809	CRIP2	cysteine rich protein 2	P52943	0.0015	1.94
ENSG00000170323	FABP4	fatty acid binding protein 4	E7DVW4	0.002	1.94
ENSG00000211677	IGLC2	immunoglobulin lambda constant 2	P0DOY2	0.014	1.94
ENSG00000197956	S100A6	S100 calcium binding protein A6	P06703	0.0019	1.94
ENSG00000175130	MARCKSL1	MARCKS like 1	P49006	0.0037	1.92
ENSG00000148834	GSTO1	glutathione S-transferase omega 1	P78417	0.0023	1.9
ENSG00000108671	PSMD11	proteasome 26S subunit, non-ATPase 11	O00231	0.0013	1.9
ENSG00000168385	SEPTIN2	septin 2	Q15019	0.013	1.9
ENSG00000122545	SEPTIN7	septin 7	A8K3D0	0.003	1.9
ENSG00000057757	PITHD1	PITH domain containing 1	Q9GZP4	0.0019	1.88
ENSG00000233927	RPS28	ribosomal protein S28	B2R4R9	0.03	1.88
ENSG00000167526	RPL13	ribosomal protein L13	A8K4C8	0.0024	1.86
ENSG00000086598	TMED2	transmembrane p24 trafficking protein 2	Q15363	0.014	1.86
ENSG00000165280	VCP	valosin containing protein	P55072	0.0032	1.86
ENSG00000112306	RPS12	ribosomal protein S12	P25398	0.0031	1.85
ENSG00000108774	RAB5C	RAB5C, member RAS oncogene family	P51148	0.0096	1.84
ENSG00000138385	SSB	small RNA binding exonuclease protection factor La	P05455	0.0018	1.84
ENSG00000126524	SBDS	SBDS ribosome maturation factor	A0A0S2Z5I7	0.003	1.83
ENSG00000140612	SEC11A	SEC11 homolog A, signal peptidase complex subunit	P67812	0.022	1.83
ENSG00000176986	SEC24C	SEC24 homolog C, COPII coat complex component	A0A024QZM6	0.017	1.83
ENSG00000023318	ERP44	endoplasmic reticulum protein 44	Q9BS26	0.0021	1.82
ENSG00000100034	PPM1F	protein phosphatase, Mg2+/Mn2+ dependent 1F	P49593	0.048	1.82
ENSG00000067560	RHOA	ras homolog family member A	A0A024R324	0.0014	1.82
ENSG00000177600	RPLP2	ribosomal protein lateral stalk subunit P2	A0A024RCA7	0.0071	1.82
ENSG00000144848	ATG3	autophagy related 3	Q9NT62	0.0019	1.81
ENSG00000114867	EIF4G1	eukaryotic translation initiation factor 4 gamma 1	B2RU06	0.0023	1.8
ENSG00000106803	SEC61B	SEC61 translocon subunit beta	P60468	0.0059	1.8

ENSG00000058668	ATP2B4	ATPase plasma membrane Ca2+ transporting 4	P23634	0.003	1.79
ENSG00000214078	CPNE1	copine 1	B0QZ18	0.042	1.77
ENSG00000144713	RPL32	ribosomal protein L32	A0A024R2G7	0.0022	1.77
ENSG00000101474	APMAP	adipocyte plasma membrane associated protein	Q9HDC9	0.0041	1.76
ENSG00000150753	CCT5	chaperonin containing TCP1 subunit 5	B4DX08	0.0029	1.76
ENSG00000126457	PRMT1	protein arginine methyltransferase 1	A0A087X1W2	0.0043	1.76
ENSG00000069329	VPS35	VPS35 retromer complex component	Q96QK1	0.0037	1.76
ENSG00000159176	CSRP1	cysteine and glycine rich protein 1	B4DY28	0.0026	1.75
ENSG00000137509	PRCP	prolylcarboxypeptidase	B7Z7Q6	0.043	1.75
ENSG00000130429	ARPC1B	actin related protein 2/3 complex subunit 1B	A4D275	0.002	1.74
ENSG00000136688	IL36G	interleukin 36 gamma	Q9NZH8	0.023	1.74
ENSG00000132341	RAN	RAN, member RAS oncogene family	B4DV51	0.0032	1.74
ENSG00000126602	TRAP1	TNF receptor associated protein 1	Q12931	0.025	1.73
ENSG00000213585	VDAC1	voltage dependent anion channel 1	A0A1L1UHR1	0.0093	1.73
ENSG00000110880	CORO1C	coronin 1C	Q9ULV4	0.01	1.72
ENSG00000104529	EEF1D	eukaryotic translation elongation factor 1 delta	B2RAR6	0.0069	1.71
ENSG00000239672	NME1	NME/NM23 nucleoside diphosphate kinase 1	P15531	0.01	1.71
ENSG00000168906	MAT2A	methionine adenosyltransferase 2A	A0A140VJP5	0.013	1.7
ENSG00000122406	RPL5	ribosomal protein L5	A2RUM7	0.0035	1.7
ENSG00000138768	USO1	USO1 vesicle transport factor	O60763	0.026	1.7
ENSG00000111530	CAND1	cullin associated and neddylation dissociated 1	Q86VP6	0.004	1.69
ENSG00000134440	NARS1	asparaginyl-tRNA synthetase 1	O43776	0.022	1.69
ENSG00000189221	MAOA	monoamine oxidase A	P21397	0.0046	1.68
ENSG00000131828	PDHA1	pyruvate dehydrogenase E1 subunit alpha 1	A0A024RBX9	0.013	1.68
ENSG00000172354	GNB2	G protein subunit beta 2	P62879	0.013	1.67
ENSG00000100387	RBX1	ring-box 1	P62877	0.0054	1.67
ENSG00000136942	RPL35	ribosomal protein L35	P42766	0.023	1.66
ENSG00000072210	ALDH3A2	aldehyde dehydrogenase 3 family member A2	P51648	0.0027	1.65
ENSG00000117592	PRDX6	peroxiredoxin 6	P30041	0.0034	1.64
ENSG00000164733	CTSB	cathepsin B	Q5HYG5	0.0039	1.62
ENSG00000273749	CYFIP1	cytoplasmic FMR1 interacting protein 1	Q7L576	0.0083	1.61
ENSG00000116560	SFPQ	splicing factor proline and glutamine rich	P23246	0.014	1.61
ENSG00000110108	TMEM109	transmembrane protein 109	Q9BVC6	0.018	1.6
ENSG00000177469	CAVIN1	caveolae associated protein 1	Q6NZI2	0.0068	1.59
ENSG00000021355	SERPINB1	serpin family B member 1	P30740	0.0056	1.59
ENSG00000099797	TECR	trans-2,3-enoyl-CoA reductase	B3KSQ1	0.022	1.59
ENSG00000172757	CFL1	cofilin 1	P23528	0.0053	1.58

ENSG00000119396	RAB14	RAB14, member RAS oncogene family	A0A024R845	0.017		1.58
ENSG00000075785	RAB7A	RAB7A, member RAS oncogene family	A0A158RFU6	0.008		1.58
ENSG00000275895	U2AF1L5	U2 small nuclear RNA auxiliary factor 1-like 5	B5BU08	0.0091		1.57
ENSG00000111640	GAPDH	glyceraldehyde-3-phosphate dehydrogenase	P04406	0.0042		1.56
ENSG00000111144	LTA4H	leukotriene A4 hydrolase	A0A140VK27	0.0071		1.56
ENSG00000130755	GMFG	glia maturation factor gamma	M0R1D2	0.0056		1.55
ENSG00000150593	PDCD4	programmed cell death 4	B4DKX4	0.026		1.55
ENSG00000152952	PLOD2	procollagen-lysine,2-oxoglutarate 5-dioxygenase 2	O00469	0.0051		1.55
ENSG00000158710	TAGLN2	transgelin 2	P37802	0.006		1.55
ENSG00000205542	TMSB4X	thymosin beta 4 X-linked	A2VCK8	0.035		1.55
ENSG00000124557	BTN1A1	butyrophilin subfamily 1 member A1	Q13410	0.0076		1.54
ENSG00000102393	GLA	galactosidase alpha	P06280	0.0083		1.54
ENSG00000116251	RPL22	ribosomal protein L22	P35268	0.0053		1.54
ENSG00000084774	CAD	carbamoyl-phosphate synthetase 2, aspartate transcarbamylase, and dihydroorotase	F8VPD4	0.045		1.52
ENSG00000130811	EIF3G	eukaryotic translation initiation factor 3 subunit G	O75821	0.0091		1.52
ENSG00000078369	GNB1	G protein subunit beta 1	B3KVK2	0.02		1.52
ENSG00000100028	SNRPD3	small nuclear ribonucleoprotein D3 polypeptide	P62318	0.0079		1.52
ENSG00000206503	HLA-A	major histocompatibility complex, class I, A	P30443	0.0044		1.51
ENSG00000146731	CCT6A	chaperonin containing TCP1 subunit 6A	P40227	0.0097		1.49
ENSG00000167656	LY6D	lymphocyte antigen 6 family member D	Q14210	0.036		1.49
ENSG00000147604	RPL7	ribosomal protein L7	P18124	0.0059		1.48
ENSG00000128245	YWHAH	tyrosine 3-monooxygenase/tryptophan 5-monooxygenase activation protein eta	A0A024R1K7	0.026		1.48
ENSG00000163466	ARPC2	actin related protein 2/3 complex subunit 2	O15144	0.016		1.47
ENSG00000129351	ILF3	interleukin enhancer binding factor 3	Q12906	0.0071		1.47
ENSG00000058262	SEC61A1	SEC61 translocon subunit alpha 1	B3KME8	0.015		1.46
ENSG00000085063	CD59	CD59 molecule (CD59 blood group)	P13987	0.012		1.44
ENSG00000134333	LDHA	lactate dehydrogenase A	P00338	0.014		1.44
ENSG00000015479	MATR3	matrin 3	A0A0R4J2E8	0.022	1.43	
ENSG00000141522	ARHGDIA	Rho GDP dissociation inhibitor alpha	P52565	0.03	1.42	
ENSG00000179085	DPM3	dolichyl-phosphate mannosyltransferase subunit 3, regulatory	A0A140VJI4	0.015	1.42	
ENSG00000125868	DSTN	destrin, actin depolymerizing factor	P60981	0.0082	1.42	
ENSG00000239264	TXNDC5	thioredoxin domain containing 5	A0A024QZV0	0.019	1.42	
ENSG00000145425	RPS3A	ribosomal protein S3A	P61247	0.018	1.41	

ENSG00000196419	XRCC6	X-ray repair cross complementing 6	A0A024R1N4	0.041	1.41
ENSG00000134308	YWHAQ	tyrosine 3-monooxygenase/tryptophan 5-monooxygenase activation protein theta	P27348	0.0093	1.41
ENSG00000133313	CNDP2	carnosine dipeptidase 2	Q96KP4	0.0093	1.4
ENSG00000108654	DDX5	DEAD-box helicase 5	P17844	0.0095	1.4
ENSG00000259207	ITGB3	integrin subunit beta 3	P05106	0.048	1.4
ENSG00000107438	PDLIM1	PDZ and LIM domain 1	O00151	0.018	1.4
ENSG00000108107	RPL28	ribosomal protein L28	P46779	0.04	1.4
ENSG00000124939	SCGB2A1	secretoglobin family 2A member 1	O75556	0.047	1.4
ENSG00000108828	VAT1	vesicle amine transport 1	A0A024R1Z6	0.037	1.4
ENSG00000083845	RPS5	ribosomal protein S5	A0A024R4Q8	0.024	1.38
ENSG00000153187	HNRNPU	heterogeneous nuclear ribonucleoprotein U	Q00839	0.02	1.37
ENSG00000142864	SERBP1	SERPINE1 mRNA binding protein 1	Q8NC51	0.039	1.36
ENSG00000213719	CLIC1	chloride intracellular channel 1	O00299	0.017	1.35
ENSG00000106105	GARS1	glycyl-tRNA synthetase 1	A0A090N8G0	0.047	1.35
ENSG00000118680	MYL12B	myosin light chain 12B	O14950	0.0089	1.35
ENSG00000149273	RPS3	ribosomal protein S3	P23396	0.025	1.35
ENSG00000130402	ACTN4	actinin alpha 4	A0A0S2Z3G9	0.0097	1.34
ENSG00000148672	GLUD1	glutamate dehydrogenase 1	P00367	0.015	1.34
ENSG00000168028	RPSA	ribosomal protein SA	A0A0C4DG17	0.015	1.34
ENSG00000100823	APEX1	apurinic/apyrimidinic endodeoxyribonuclease 1	P27695	0.011	1.33
ENSG00000167460	TPM4	tropomyosin 4	P67936	0.028	1.33
ENSG00000254772	EEF1G	eukaryotic translation elongation factor 1 gamma	P26641	0.0097	1.32
ENSG00000100097	LGALS1	galectin 1	P09382	0.015	1.32
ENSG00000089157	RPLP0	ribosomal protein lateral stalk subunit P0	A0A024RBS2	0.01	1.32
ENSG00000104763	ASAH1	N-acylsphingosine amidohydrolase 1	A8K0B6	0.031	1.31
ENSG00000136160	EDNRB	endothelin receptor type B	P24530	0.031	1.29
ENSG00000196531	NACA	nascent polypeptide associated complex subunit alpha	A0A024RB41	0.021	1.29
ENSG00000067113	PLPP1	phospholipid phosphatase 1	A0A024QZS3	0.047	1.29
ENSG00000105568	PPP2R1A	protein phosphatase 2 scaffold subunit Aalpha	A8K7B7	0.047	1.29
ENSG00000134871	COL4A2	collagen type IV alpha 2 chain	A0A024RDW8	0.029	1.28
ENSG00000145349	CAMK2D	calcium/calmodulin dependent protein kinase II delta	A0A024RDK3	0.031	1.27
ENSG00000167468	GPX4	glutathione peroxidase 4	P36969	0.039	1.27
ENSG00000103415	HMOX2	heme oxygenase 2	P30519	0.012	1.27
ENSG00000162909	CAPN2	calpain 2	B4DN77	0.032	1.25
ENSG00000137154	RPS6	ribosomal protein S6	A2A3R6	0.019	1.25
ENSG00000108518	PFN1	profilin 1	P07737	0.047	1.24
ENSG00000169504	CLIC4	chloride intracellular channel 4	Q6FIC5	0.012	1.23

ENSG00000138095	LRPPRC	leucine rich pentatricopeptide repeat containing	E5KNY5	0.023	1.23
ENSG00000089009	RPL6	ribosomal protein L6	A0A024RBK3	0.025	1.23
ENSG00000164924	YWHAZ	tyrosine 3-monooxygenase/tryptophan 5-monooxygenase activation protein zeta	D0PNI1	0.015	1.23
ENSG00000128591	FLNC	filamin C	Q14315	0.013	1.22
ENSG00000148484	RSU1	Ras suppressor protein 1	Q15404	0.02	1.22
ENSG00000100934	SEC23A	SEC23 homolog A, COPII coat complex component	Q15436	0.025	1.22
ENSG00000204628	RACK1	receptor for activated C kinase 1	E9KL35	0.028	1.21
ENSG00000100714	MTHFD1	methylenetetrahydrofolate dehydrogenase, cyclohydrolase and formyltetrahydrofolate synthetase 1	P11586	0.021	1.2
ENSG00000135636	DYSF	dysferlin	O75923	0.014	1.19
ENSG00000161960	EIF4A1	eukaryotic translation initiation factor 4A1	P60842	0.021	1.19
ENSG00000185624	P4HB	prolyl 4-hydroxylase subunit beta	A0A024R8S5	0.034	1.19
ENSG00000079246	XRCC5	X-ray repair cross complementing 5	P13010	0.035	1.19
ENSG00000129562	DAD1	defender against cell death 1	P61803	0.047	1.18
ENSG00000168374	ARF4	ADP ribosylation factor 4	P18085	0.036	1.16
ENSG00000092964	DPYSL2	dihydropyrimidinase like 2	A0A1C7CYX9	0.026	1.16
ENSG00000196586	MYO6	myosin VI	Q9UM54	0.026	1.16
ENSG00000060138	YBX3	Y-box binding protein 3	A0A024RAV4	0.043	1.16
ENSG00000171314	PGAM1	phosphoglycerate mutase 1	B7Z9E5	0.019	1.15
ENSG00000004700	RECQL	RecQ like helicase	A0A024RAV2	0.047	1.15
ENSG00000163468	CCT3	chaperonin containing TCP1 subunit 3	B3KX11	0.02	1.14
ENSG00000100316	RPL3	ribosomal protein L3	P39023	0.039	1.14
ENSG00000075415	SLC25A3	solute carrier family 25 member 3	A0A024RBE8	0.039	1.14
ENSG00000174437	ATP2A2	ATPase sarcoplasmic/endoplasmic reticulum Ca2+ transporting 2	P16615	0.031	1.12
ENSG00000196205	EEF1A1P5	eukaryotic translation elongation factor 1 alpha 1 pseudogene 5	Q5VTE0	0.046	1.11
ENSG00000120694	HSPH1	heat shock protein family H (Hsp110) member 1	A0A024RDQ0	0.034	1.11
ENSG00000175166	PSMD2	proteasome 26S subunit ubiquitin receptor, non-ATPase 2	Q13200	0.032	1.11
ENSG00000170759	KIF5B	kinesin family member 5B	P33176	0.046	1.1
ENSG00000092010	PSME1	proteasome activator subunit 1	Q06323	0.023	1.1
ENSG00000197157	SND1	staphylococcal nuclease and tudor domain containing 1	A0A140VK49	0.031	1.1
ENSG00000135829	DHX9	DExH-box helicase 9	B3KU66	0.025	1.09
ENSG00000100401	RANGAP1	Ran GTPase activating protein 1	A0A024R1U0	0.044	1.09
ENSG00000198959	TGM2	transglutaminase 2	P21980	0.02	1.09
ENSG00000130741	EIF2S3	eukaryotic translation initiation factor 2 subunit gamma	P41091	0.044	1.08
ENSG00000137106	GRHPR	glyoxylate and hydroxypyruvate reductase	Q9UBQ7	0.021	1.08
ENSG00000142798	HSPG2	heparan sulfate proteoglycan 2	P98160	0.027	1.08

ENSG00000105835	NAMPT	nicotinamide phosphoribosyltransferase	A0A024R718	0.032	1.07
ENSG00000024422	EHD2	EH domain containing 2	A0A024R0S6	0.029	1.06
ENSG00000187514	PTMA	prothymosin alpha	P06454	0.046	1.04
ENSG00000189171	S100A13	S100 calcium binding protein A13	Q99584	0.033	1.03
ENSG00000096384	HSP90AB1	heat shock protein 90 alpha family class B member 1	A0A024RD80	0.034	1.02
ENSG00000163902	RPN1	ribophorin I	P04843	0.046	1.02
ENSG00000138668	HNRNPD	heterogeneous nuclear ribonucleoprotein D	Q14103	0.047	1.01
ENSG00000167085	PHB1	prohibitin 1	P35232	0.043	1.01
ENSG00000137767	SQOR	sulfide quinone oxidoreductase	A0A024R5X2	0.039	1.01
ENSG00000110799	VWF	von Willebrand factor	P04275	0.042	0.99
ENSG00000167658	EEF2	eukaryotic translation elongation factor 2	P13639	0.042	0.98
ENSG00000136068	FLNB	filamin B	O75369	0.04	0.96
ENSG00000138772	ANXA3	annexin A3	P12429	0.045	0.94
ENSG00000127022	CANX	calnexin	P27824	0.049	0.94
ENSG00000135316	SYNCRIP	synaptotagmin binding cytoplasmic RNA interacting protein	B7Z645	0.045	0.94
ENSG00000261371	PECAM1	platelet and endothelial cell adhesion molecule 1	A0A075B738	0.047	0.93
ENSG00000140988	RPS2	ribosomal protein S2	P15880	0.044	0.92
ENSG00000077549	CAPZB	capping actin protein of muscle Z-line subunit beta	P47756	0.049	0.87

Adjustments of p-values for multiple comparisons were used with Benjamini-Hochberg (BH) correction.

Supplemental Table S3. Insulin induced palmitoylated proteins in human LECs.

ENSEMBL ID	Gene Symbol	Description	UNIPROT	*p*-value	-Log Ratio (+/-HA)
ENSG00000054116	TRAPPC3	trafficking protein particle complex subunit 3	A0A087WWM0	1.10E-06	6.86
ENSG00000102007	PLP2	proteolipid protein 2	A0A024QYW3	2.80E-05	6.11
ENSG00000114698	PLSCR4	phospholipid scramblase 4	Q9NRQ2	5.40E-07	5.97
ENSG00000126458	RRAS	RAS related	A0A024QZF2	7.40E-07	5.94
ENSG00000156052	GNAQ	G protein subunit alpha q	A0A024R240	8.20E-07	5.91
ENSG00000120063	GNA13	G protein subunit alpha 13	Q14344	2.40E-06	5.81
ENSG00000099864	PALM	paralemmin	A0A024R207	1.30E-06	5.69
ENSG00000162618	ADGRL4	adhesion G protein-coupled receptor L4	Q9HBW9	2.00E-06	5.66
ENSG00000108219	TSPAN14	tetraspanin 14	Q8NG11	3.20E-06	5.47
ENSG00000183726	TMEM50A	transmembrane protein 50A	O95807	1.20E-05	5.46
ENSG00000111897	SERINC1	serine incorporator 1	Q9NRX5	2.80E-06	5.39
ENSG00000135047	CTSL	cathepsin L	A0A024R276	2.00E-06	5.08
ENSG00000153551	CMTM7	CKLF like MARVEL transmembrane domain containing 7	A0A024R2L3	7.20E-06	4.95
ENSG00000099203	TMED1	transmembrane p24 trafficking protein 1	Q13445	3.30E-06	4.91
ENSG00000243279	PRAF2	PRA1 domain family member 2	A0A024QZ22	9.30E-06	4.88
ENSG00000169908	TM4SF1	transmembrane 4 L six family member 1	P30408	1.10E-05	4.84
ENSG00000227500	SCAMP4	secretory carrier membrane protein 4	Q969E2	4.00E-06	4.82
ENSG00000002586	CD99	CD99 molecule (Xg blood group)	P14209	2.70E-06	4.78
ENSG00000116754	SRSF11	serine and arginine rich splicing factor 11	Q05519	1.10E-05	4.73
ENSG00000249751	ECSCR	endothelial cell surface expressed chemotaxis and apoptosis regulator	Q19T08	6.70E-06	4.65
ENSG00000013297	CLDN11	claudin 11	O75508	7.10E-06	4.63
ENSG00000147533	GOLGA7	golgin A7	Q7Z5G4	5.90E-06	4.62
ENSG00000142089	IFITM3	interferon induced transmembrane protein 3	Q01628	4.90E-06	4.39
ENSG00000026508	CD44	CD44 molecule (Indian blood group)	P16070	4.10E-06	4.36
ENSG00000092531	SNAP23	synaptosome associated protein 23	A8K287	1.30E-05	4.34
ENSG00000159140	SON	SON DNA and RNA binding protein	P18583	2.70E-05	4.28
ENSG00000177697	CD151	CD151 molecule (Raph blood group)	A0A024RCB3	1.20E-05	4.21
ENSG00000139921	TMX1	thioredoxin related transmembrane protein 1	Q9H3N1	7.10E-06	4.19
ENSG00000136156	ITM2B	integral membrane protein 2B	Q9Y287	2.60E-05	4.11
ENSG00000135821	GLUL	glutamate-ammonia ligase	A8YXX4	0.021	4.1
ENSG00000140497	SCAMP2	secretory carrier membrane protein 2	A8K769	8.40E-06	4.1
ENSG00000088256	GNA11	G protein subunit alpha 11	P29992	3.70E-05	4.08

ENSG00000123728	RAP2C	RAP2C, member of RAS oncogene family	Q9Y3L5	6.50E-06	4.04
ENSG00000129625	REEP5	receptor accessory protein 5	Q00765	2.30E-05	4.04
ENSG00000213699	SLC35F6	solute carrier family 35 member F6	Q8N357	4.00E-05	3.97
ENSG00000133818	RRAS2	RAS related 2	P62070	1.30E-05	3.84
ENSG00000144959	NCEH1	neutral cholesterol ester hydrolase 1	A0A0A0MTJ9	4.60E-05	3.82
ENSG00000010278	CD9	CD9 molecule	A6NNI4	5.10E-05	3.81
ENSG00000114062	UBE3A	ubiquitin protein ligase E3A	Q05086	4.10E-05	3.79
ENSG00000185651	UBE2L3	ubiquitin conjugating enzyme E2 L3	P68036	2.80E-05	3.73
ENSG00000057149	SERPINB3	serpin family B member 3	P29508	0.0068	3.7
ENSG00000132388	UBE2G1	ubiquitin conjugating enzyme E2 G1	P62253	2.40E-04	3.69
ENSG00000181104	F2R	coagulation factor II thrombin receptor	P25116	2.70E-05	3.67
ENSG00000004468	CD38	CD38 molecule	B4E006	8.00E-05	3.66
ENSG00000143878	RHOB	ras homolog family member B	P62745	8.60E-05	3.66
ENSG00000100258	LMF2	lipase maturation factor 2	Q9BU23	4.20E-05	3.65
ENSG00000133226	SRRM1	serine and arginine repetitive matrix 1	B7Z7U0	1.90E-05	3.65
ENSG00000136026	CKAP4	cytoskeleton associated protein 4	A0A024RBH2	5.50E-05	3.64
ENSG00000110651	CD81	CD81 molecule	E9PJK1	2.00E-04	3.63
ENSG00000108848	LUC7L3	LUC7 like 3 pre-mRNA splicing factor	J3KPP4	3.40E-05	3.61
ENSG00000003056	M6PR	mannose-6-phosphate receptor, cation dependent	F5GX30	1.70E-04	3.58
ENSG00000148175	STOM	stomatin	F8VSL7	2.40E-05	3.58
ENSG00000104852	SNRNP70	small nuclear ribonucleoprotein U1 subunit 70	P08621	2.70E-05	3.46
ENSG00000213625	LEPROT	leptin receptor overlapping transcript	A0A087X0N2	8.20E-05	3.42
ENSG00000206073	SERPINB4	serpin family B member 4	P48594	0.023	3.42
ENSG00000076706	MCAM	melanoma cell adhesion molecule	A0A024R3I5	4.20E-05	3.41
ENSG00000188313	PLSCR1	phospholipid scramblase 1	O15162	7.40E-05	3.41
ENSG00000063854	HAGH	hydroxyacylglutathione hydrolase	Q16775	0.022	3.4
ENSG00000130725	UBE2M	ubiquitin conjugating enzyme E2 M	A0A024R4T4	3.90E-05	3.37
ENSG00000146411	SLC2A12	solute carrier family 2 member 12	Q8TD20	5.50E-04	3.36
ENSG00000049245	VAMP3	vesicle associated membrane protein 3	Q15836	4.40E-05	3.36
ENSG00000120437	ACAT2	acetyl-CoA acetyltransferase 2	Q9BWD1	9.60E-05	3.35
ENSG00000137497	NUMA1	nuclear mitotic apparatus protein 1	A0A024R5M9	1.80E-04	3.35
ENSG00000143222	UFC1	ubiquitin-fold modifier conjugating enzyme 1	Q9Y3C8	1.10E-04	3.35
ENSG00000132589	FLOT2	flotillin 2	J3QLD9	1.20E-04	3.31
ENSG00000078140	UBE2K	ubiquitin conjugating enzyme E2 K	P61086	4.10E-05	3.25

ENSG00000135124	P2RX4	purinergic receptor P2X 4	Q99571	9.10E-05	3.2
ENSG00000110917	MLEC	malectin	F5GX14	7.10E-05	3.17
ENSG00000112739	PRPF4B	pre-mRNA processing factor 4B	A0A024QZY5	1.40E-04	3.17
ENSG00000109084	TMEM97	transmembrane protein 97	Q5BJF2	0.0093	3.16
ENSG00000184470	TXNRD2	thioredoxin reductase 2	E7EWK1	9.70E-05	3.14
ENSG00000112531	QKI	QKI, KH domain containing RNA binding	Q96PU8	1.20E-04	3.13
ENSG00000072401	UBE2D1	ubiquitin conjugating enzyme E2 D1	A0A087WW00	7.30E-04	3.11
ENSG00000125148	MT2A	metallothionein 2A	P02795	8.90E-04	3.08
ENSG00000168899	VAMP5	vesicle associated membrane protein 5	O95183	1.10E-04	3.04
ENSG00000136688	IL36G	interleukin 36 gamma	Q9NZH8	8.40E-04	2.96
ENSG00000157227	MMP14	matrix metallopeptidase 14	P50281	7.70E-05	2.96
ENSG00000254087	LYN	LYN proto-oncogene, Src family tyrosine kinase	P07948	2.20E-04	2.92
ENSG00000134247	PTGFRN	prostaglandin F2 receptor inhibitor	Q9P2B2	1.50E-04	2.91
ENSG00000072274	TFRC	transferrin receptor	P02786	2.60E-04	2.9
ENSG00000145833	DDX46	DEAD-box helicase 46	A0A0C4DG89	1.90E-04	2.85
ENSG00000000003	TSPAN6	tetraspanin 6	A0A024RCI0	0.0021	2.85
ENSG00000177889	UBE2N	ubiquitin conjugating enzyme E2 N	P61088	4.10E-04	2.8
ENSG00000143546	S100A8	S100 calcium binding protein A8	P05109	0.046	2.79
ENSG00000139433	GLTP	glycolipid transfer protein	A0A024RBI7	0.0011	2.78
ENSG00000130985	UBA1	ubiquitin like modifier activating enzyme 1	A0A024R1A3	1.10E-04	2.77
ENSG00000198833	UBE2J1	ubiquitin conjugating enzyme E2 J1	Q9Y385	3.10E-04	2.76
ENSG00000187688	TRPV2	transient receptor potential cation channel subfamily V member 2	Q9Y5S1	1.40E-04	2.73
ENSG00000084674	APOB	apolipoprotein B	P04114	0.0062	2.7
ENSG00000205076	LGALS7	galectin 7	P47929	0.041	2.7
ENSG00000184363	PKP3	plakophilin 3	Q9Y446	0.031	2.61
ENSG00000184113	CLDN5	claudin 5	D3DX19	4.80E-04	2.58
ENSG00000146963	LUC7L2	LUC7 like 2, pre-mRNA splicing factor	Q9Y383	3.10E-04	2.55
ENSG00000205937	RNPS1	RNA binding protein with serine rich domain 1	D3DU92	0.0054	2.53
ENSG00000204568	MRPS18B	mitochondrial ribosomal protein S18B	B0S7P4	0.0011	2.52
ENSG00000144848	ATG3	autophagy related 3	Q9NT62	2.60E-04	2.5
ENSG00000131378	RFTN1	raftlin, lipid raft linker 1	Q14699	4.30E-04	2.5
ENSG00000066056	TIE1	tyrosine kinase with immunoglobulin like and EGF like domains 1	B4DTW8	0.001	2.47
ENSG00000014257	ACP3	acid phosphatase 3	P15309	0.035	2.46
ENSG00000168394	TAP1	transporter 1, ATP binding cassette subfamily B member	A0A0S2Z5A6	2.80E-04	2.39
ENSG00000004961	HCCS	holocytochrome c synthase	A0A024RBY9	7.70E-04	2.33
ENSG00000138760	SCARB2	scavenger receptor class B member 2	Q14108	3.90E-04	2.32

ENSG00000198431	TXNRD1	thioredoxin reductase 1	Q16881	0.002	2.32
ENSG00000101160	CTSZ	cathepsin Z	Q9UBR2	0.0013	2.3
ENSG00000129353	SLC44A2	solute carrier family 44 member 2	A0A088QCU6	5.40E-04	2.3
ENSG00000074696	HACD3	3-hydroxyacyl-CoA dehydratase 3	Q9P035	0.0012	2.28
ENSG00000139180	NDUFA9	NADH:ubiquinone oxidoreductase subunit A9	Q16795	0.034	2.27
ENSG00000150768	DLAT	dihydrolipoamide S-acetyltransferase	P10515	0.0011	2.26
ENSG00000183291	SELENOF	selenoprotein F	O60613	5.70E-04	2.26
ENSG00000233276	GPX1	glutathione peroxidase 1	P07203	5.10E-04	2.25
ENSG00000159202	UBE2Z	ubiquitin conjugating enzyme E2 Z	Q9H832	6.60E-04	2.24
ENSG00000144118	RALB	RAS like proto-oncogene B	A0A024RAG3	6.60E-04	2.23
ENSG00000144744	UBA3	ubiquitin like modifier activating enzyme 3	Q8TBC4	0.0015	2.2
ENSG00000197728	RPS26	ribosomal protein S26	A0A024RB14	0.0096	2.19
ENSG00000065135	GNAI3	G protein subunit alpha i3	P08754	7.80E-04	2.18
ENSG00000170889	RPS9	ribosomal protein S9	A0A024R4M0	0.0016	2.18
ENSG00000017260	ATP2C1	ATPase secretory pathway Ca2+ transporting 1	P98194	8.00E-04	2.16
ENSG00000025708	TYMP	thymidine phosphorylase	B2RBL3	0.048	2.16
ENSG00000135218	CD36	CD36 molecule	A4D1B1	0.0014	2.13
ENSG00000126261	UBA2	ubiquitin like modifier activating enzyme 2	Q9UBT2	0.0011	2.12
ENSG00000239306	RBM14	RNA binding motif protein 14	A0A0S2Z567	0.038	2.09
ENSG00000147419	CCDC25	coiled-coil domain containing 25	G3V121	0.04	2.05
ENSG00000147649	MTDH	metadherin	A0A024R9D2	7.80E-04	2.05
ENSG00000167754	KLK5	kallikrein related peptidase 5	Q9Y337	0.0093	2.04
ENSG00000103018	CYB5B	cytochrome b5 type B	J3KNF8	0.00093	2.02
ENSG00000138119	MYOF	myoferlin	Q9NZM1	0.0016	2
ENSG00000156026	MCU	mitochondrial calcium uniporter	Q8NE86	0.0053	1.99
ENSG00000033178	UBA6	ubiquitin like modifier activating enzyme 6	A0A024RDB0	0.0016	1.93
ENSG00000114353	GNAI2	G protein subunit alpha i2	B3KP24	0.0018	1.86
ENSG00000155380	SLC16A1	solute carrier family 16 member 1	A0A024R0H1	0.01	1.86
ENSG00000136160	EDNRB	endothelin receptor type B	P24530	0.0066	1.82
ENSG00000105971	CAV2	caveolin 2	P51636	0.0056	1.81
ENSG00000063177	RPL18	ribosomal protein L18	A0A024QZD1	0.0015	1.81
ENSG00000006451	RALA	RAS like proto-oncogene A	P11233	0.0025	1.77
ENSG00000087086	FTL	ferritin light chain	P02792	0.0073	1.76
ENSG00000130193	THEM6	thioesterase superfamily member 6	Q8WUY1	0.0058	1.76
ENSG00000177674	AGTRAP	angiotensin II receptor associated protein	Q6RW13	0.026	1.73
ENSG00000131016	AKAP12	A-kinase anchoring protein 12	Q02952	0.0036	1.72
ENSG00000066654	THUMPD1	THUMP domain containing 1	A0A024R388	0.018	1.72

ENSG00000206341	HLA-H	major histocompatibility complex, class I, H (pseudogene)	P01893	0.043	1.71
ENSG00000169710	FASN	fatty acid synthase	P49327	0.0037	1.67
ENSG00000073008	PVR	PVR cell adhesion molecule	P15151	0.0049	1.65
ENSG00000196329	GIMAP5	GTPase, IMAP family member 5	A0A090N8P9	0.022	1.64
ENSG00000115310	RTN4	reticulon 4	Q9NQC3	0.0039	1.63
ENSG00000141526	SLC16A3	solute carrier family 16 member 3	A0A024R8U1	0.008	1.63
ENSG00000127824	TUBA4A	tubulin alpha 4a	P68366	0.0081	1.63
ENSG00000113441	LNPEP	leucyl and cystinyl aminopeptidase	Q9UIQ6	0.012	1.62
ENSG00000116350	SRSF4	serine and arginine rich splicing factor 4	Q08170	0.025	1.59
ENSG00000130255	RPL36	ribosomal protein L36	Q9Y3U8	0.012	1.55
ENSG00000075239	ACAT1	acetyl-CoA acetyltransferase 1	A0A140VJX1	0.0063	1.5
ENSG00000158270	COLEC12	collectin subfamily member 12	Q5KU26	0.013	1.48
ENSG00000138029	HADHB	hydroxyacyl-CoA dehydrogenase trifunctional multienzyme complex subunit beta	P55084	0.021	1.48
ENSG00000074755	ZZEF1	zinc finger ZZ-type and EF-hand domain containing 1	O43149	0.0096	1.45
ENSG00000167996	FTH1	ferritin heavy chain 1	A0A024R525	0.0071	1.44
ENSG00000145349	CAMK2D	calcium/calmodulin dependent protein kinase II delta	A0A024RDK3	0.019	1.43
ENSG00000111640	GAPDH	glyceraldehyde-3-phosphate dehydrogenase	P04406	0.0076	1.39
ENSG00000148634	HERC4	HECT and RLD domain containing E3 ubiquitin protein ligase 4	Q5GLZ8	0.007	1.36
ENSG00000198561	CTNND1	catenin delta 1	O60716	0.024	1.35
ENSG00000144713	RPL32	ribosomal protein L32	A0A024R2G7	0.0093	1.35
ENSG00000133318	RTN3	reticulon 3	O95197	0.026	1.35
ENSG00000117298	ECE1	endothelin converting enzyme 1	A0A024RAB0	0.013	1.34
ENSG00000262814	MRPL12	mitochondrial ribosomal protein L12	P52815	0.019	1.34
ENSG00000174444	RPL4	ribosomal protein L4	P36578	0.0096	1.32
ENSG00000105974	CAV1	caveolin 1	A0A024R757	0.013	1.3
ENSG00000103415	HMOX2	heme oxygenase 2	P30519	0.012	1.28
ENSG00000109475	RPL34	ribosomal protein L34	A0A024RDH8	0.029	1.27
ENSG00000197081	IGF2R	insulin like growth factor 2 receptor	P11717	0.034	1.19
ENSG00000115159	GPD2	glycerol-3-phosphate dehydrogenase 2	P43304	0.024	1.18
ENSG00000198380	GFPT1	glutamine--fructose-6-phosphate transaminase 1	Q06210	0.026	1.16
ENSG00000166479	TMX3	thioredoxin related transmembrane protein 3	Q96JJ7	0.027	1.08
ENSG00000184584	STING1	stimulator of interferon response cGAMP interactor 1	V5V0K2	0.044	1.07

ENSG00000119711	ALDH6A1	aldehyde dehydrogenase 6 family member A1	Q02252	0.036	1.06
ENSG00000087460	GNAS	GNAS complex locus	A0A0S2Z3H8	0.031	1.06
ENSG00000137845	ADAM10	ADAM metallopeptidase domain 10	A0A024R5U5	0.042	1.01
ENSG00000137767	SQOR	sulfide quinone oxidoreductase	A0A024R5X2	0.042	0.99
ENSG00000161011	SQSTM1	sequestosome 1	Q13501	0.038	0.94

Adjustments of p-values for multiple comparisons were used with Benjamini-Hochberg (BH) correction.

Supplemental Table S4. Palmitoylated proteins in siCD36 LECs (media).

ENSEMBL ID	Gene Symbol	Description	UNIPROT	*p*-value	-Log Ratio (+/- HA)
ENSG00000054116	TRAPPC3	trafficking protein particle complex subunit 3	A0A087WWM0	2.70E-07	7.2
ENSG00000116521	SCAMP3	secretory carrier membrane protein 3	O14828	3.40E-07	6.63
ENSG00000136156	ITM2B	integral membrane protein 2B	Q9Y287	3.10E-07	6.58
ENSG00000137312	FLOT1	flotillin 1	O75955	6.90E-07	6.57
ENSG00000114698	PLSCR4	phospholipid scramblase 4	Q9NRQ2	5.10E-07	6.53
ENSG00000110651	CD81	CD81 molecule	E9PJK1	3.40E-07	6.44
ENSG00000133818	RRAS2	RAS related 2	P62070	6.10E-07	6.44
ENSG00000002586	CD99	CD99 molecule (Xg blood group)	P14209	3.20E-07	6.35
ENSG00000132824	SERINC3	serine incorporator 3	Q13530	5.10E-07	6.3
ENSG00000213699	SLC35F6	solute carrier family 35 member F6	Q8N357	1.00E-06	6.2
ENSG00000169908	TM4SF1	transmembrane 4 L six family member 1	P30408	4.50E-07	6.03
ENSG00000177889	UBE2N	ubiquitin conjugating enzyme E2 N	P61088	4.10E-07	6.02
ENSG00000177697	CD151	CD151 molecule (Raph blood group)	A0A024RCB3	2.00E-06	6
ENSG00000085365	SCAMP1	secretory carrier membrane protein 1	A0A087WXB0	4.80E-05	5.98
ENSG00000102007	PLP2	proteolipid protein 2	A0A024QYW3	4.90E-06	5.97
ENSG00000148175	STOM	stomatin	F8VSL7	9.60E-07	5.96
ENSG00000139921	TMX1	thioredoxin related transmembrane protein 1	Q9H3N1	5.50E-07	5.95
ENSG00000135047	CTSL	cathepsin L	A0A024R276	6.70E-07	5.92
ENSG00000129353	SLC44A2	solute carrier family 44 member 2	A0A088QCU6	4.70E-06	5.92
ENSG00000113811	SELENOK	selenoprotein K	Q9Y6D0	1.50E-06	5.89
ENSG00000076706	MCAM	melanoma cell adhesion molecule	A0A024R3I5	9.40E-07	5.88
ENSG00000142188	TMEM50B	transmembrane protein 50B	P56557	7.90E-07	5.87
ENSG00000087191	PSMC5	proteasome 26S subunit, ATPase 5	P62195	5.70E-07	5.82
ENSG00000142089	IFITM3	interferon induced transmembrane protein 3	Q01628	1.30E-06	5.8
ENSG00000134247	PTGFRN	prostaglandin F2 receptor inhibitor	Q9P2B2	2.50E-06	5.78
ENSG00000213281	NRAS	NRAS proto-oncogene, GTPase	P01111	4.20E-06	5.77
ENSG00000243279	PRAF2	PRA1 domain family member 2	A0A024QZ22	2.90E-06	5.76
ENSG00000010278	CD9	CD9 molecule	A6NNI4	7.00E-07	5.75
ENSG00000092531	SNAP23	synaptosome associated protein 23	A8K287	6.40E-07	5.72
ENSG00000112972	HMGCS1	3-hydroxy-3-methylglutaryl-CoA synthase 1	A0A024R059	1.60E-06	5.71
ENSG00000129625	REEP5	receptor accessory protein 5	Q00765	1.30E-06	5.68
ENSG00000187838	PLSCR3	phospholipid scramblase 3	Q9NRY6	2.80E-06	5.66
ENSG00000111897	SERINC1	serine incorporator 1	Q9NRX5	1.60E-06	5.58
ENSG00000168899	VAMP5	vesicle associated membrane protein 5	O95183	2.80E-06	5.58
ENSG00000078140	UBE2K	ubiquitin conjugating enzyme E2 K	P61086	1.40E-06	5.56
ENSG00000204632	HLA-G	major histocompatibility complex, class I, G	P17693	3.10E-06	5.55
ENSG00000049245	VAMP3	vesicle associated membrane protein 3	Q15836	1.70E-06	5.53
ENSG00000130725	UBE2M	ubiquitin conjugating enzyme E2 M	A0A024R4T4	3.10E-06	5.47
ENSG00000099864	PALM	paralemmin	A0A024R207	3.00E-06	5.46

ENSG00000175792	RUVBL1	RuvB like AAA ATPase 1	Q9Y265	1.50E-06	5.37
ENSG00000137288	UQCC2	ubiquinol-cytochrome c reductase complex assembly factor 2	Q9BRT2	1.30E-06	5.34
ENSG00000073060	SCARB1	scavenger receptor class B member 1	Q8WTV0	2.60E-06	5.29
ENSG00000143878	RHOB	ras homolog family member B	P62745	6.40E-06	5.22
ENSG00000033178	UBA6	ubiquitin like modifier activating enzyme 6	A0A024RDB0	1.70E-06	5.19
ENSG00000126261	UBA2	ubiquitin like modifier activating enzyme 2	Q9UBT2	2.50E-06	5.14
ENSG00000099282	TSPAN15	tetraspanin 15	O95858	1.80E-05	5.11
ENSG00000233276	GPX1	glutathione peroxidase 1	P07203	3.20E-06	5.08
ENSG00000144118	RALB	RAS like proto-oncogene B	A0A024RAG3	2.70E-06	5.06
ENSG00000185651	UBE2L3	ubiquitin conjugating enzyme E2 L3	P68036	1.70E-05	5.04
ENSG00000132470	ITGB4	integrin subunit beta 4	A0A024R8T0	5.10E-06	4.92
ENSG00000163466	ARPC2	actin related protein 2/3 complex subunit 2	O15144	3.60E-06	4.91
ENSG00000135404	CD63	CD63 molecule	A0A024RB05	2.70E-05	4.91
ENSG00000132388	UBE2G1	ubiquitin conjugating enzyme E2 G1	P62253	2.80E-06	4.89
ENSG00000227500	SCAMP4	secretory carrier membrane protein 4	Q969E2	3.30E-06	4.88
ENSG00000204525	HLA-C	major histocompatibility complex, class I, C	O19617	3.10E-05	4.86
ENSG00000163762	TM4SF18	transmembrane 4 L six family member 18	Q96CE8	2.10E-05	4.81
ENSG00000108219	TSPAN14	tetraspanin 14	Q8NG11	9.70E-06	4.8
ENSG00000183726	TMEM50A	transmembrane protein 50A	O95807	8.80E-06	4.77
ENSG00000078369	GNB1	G protein subunit beta 1	B3KVK2	3.00E-05	4.72
ENSG00000132589	FLOT2	flotillin 2	J3QLD9	1.70E-05	4.71
ENSG00000120437	ACAT2	acetyl-CoA acetyltransferase 2	Q9BWD1	1.50E-05	4.7
ENSG00000184113	CLDN5	claudin 5	D3DX19	1.50E-05	4.7
ENSG00000166479	TMX3	thioredoxin related transmembrane protein 3	Q96JJ7	4.60E-06	4.68
ENSG00000087460	GNAS	GNAS complex locus	A0A0S2Z3H8	1.80E-05	4.65
ENSG00000136026	CKAP4	cytoskeleton associated protein 4	A0A024RBH2	1.10E-05	4.62
ENSG00000163110	PDLIM5	PDZ and LIM domain 5	Q96HC4	9.60E-06	4.61
ENSG00000010270	STARD3NL	STARD3 N-terminal like	A0A024RA89	6.50E-05	4.6
ENSG00000157227	MMP14	matrix metallopeptidase 14	P50281	1.10E-04	4.58
ENSG00000112531	QKI	QKI, KH domain containing RNA binding	Q96PU8	3.30E-06	4.55
ENSG00000112739	PRPF4B	pre-mRNA processing factor 4B	A0A024QZY5	5.40E-06	4.52
ENSG00000167996	FTH1	ferritin heavy chain 1	A0A024R525	2.00E-05	4.5
ENSG00000100823	APEX1	apurinic/apyrimidinic endodeoxyribonuclease 1	P27695	1.90E-05	4.49
ENSG00000066322	ELOVL1	ELOVL fatty acid elongase 1	Q9BW60	1.20E-05	4.48
ENSG00000114353	GNAI2	G protein subunit alpha i2	B3KP24	9.30E-06	4.46
ENSG00000133706	LARS1	leucyl-tRNA synthetase 1	B4E266	6.10E-06	4.45
ENSG00000006451	RALA	RAS like proto-oncogene A	P11233	1.50E-05	4.44
ENSG00000121774	KHDRBS1	KH RNA binding domain containing, signal transduction associated 1	Q07666	4.00E-05	4.43
ENSG00000100764	PSMC1	proteasome 26S subunit, ATPase 1	P62191	2.00E-05	4.39
ENSG00000037280	FLT4	fms related receptor tyrosine kinase 4	P35916	1.50E-05	4.35

ENSG00000110917	MLEC	malectin	F5GX14	7.70E-06	4.34
ENSG00000035687	ADSS2	adenylosuccinate synthase 2	A0A024R5Q7	9.00E-06	4.33
ENSG00000133789	SWAP70	switching B cell complex subunit SWAP70	B3KUB9	8.50E-06	4.31
ENSG00000131711	MAP1B	microtubule associated protein 1B	A2BDK6	2.40E-05	4.29
ENSG00000142676	RPL11	ribosomal protein L11	P62913	9.40E-05	4.29
ENSG00000115310	RTN4	reticulon 4	Q9NQC3	3.10E-05	4.28
ENSG00000101294	HM13	histocompatibility minor 13	A0A0S2Z5V7	1.20E-05	4.24
ENSG00000177674	AGTRAP	angiotensin II receptor associated protein	Q6RW13	1.40E-05	4.21
ENSG00000136111	TBC1D4	TBC1 domain family member 4	O60343	1.80E-04	4.2
ENSG00000130193	THEM6	thioesterase superfamily member 6	Q8WUY1	2.00E-05	4.15
ENSG00000144848	ATG3	autophagy related 3	Q9NT62	1.50E-05	4.1
ENSG00000198561	CTNND1	catenin delta 1	O60716	1.70E-05	4.1
ENSG00000138029	HADHB	hydroxyacyl-CoA dehydrogenase trifunctional multienzyme complex subunit beta	P55084	1.10E-05	4.1
ENSG00000112118	MCM3	minichromosome maintenance complex component 3	B4DUQ9	7.40E-04	4.1
ENSG00000198431	TXNRD1	thioredoxin reductase 1	Q16881	1.80E-05	4.1
ENSG00000149218	ENDOD1	endonuclease domain containing 1	O94919	9.90E-05	4.05
ENSG00000188313	PLSCR1	phospholipid scramblase 1	O15162	6.90E-05	4.05
ENSG00000101160	CTSZ	cathepsin Z	Q9UBR2	4.90E-05	4.03
ENSG00000150768	DLAT	dihydrolipoamide S-acetyltransferase	P10515	4.00E-05	4.01
ENSG00000177469	CAVIN1	caveolae associated protein 1	Q6NZI2	3.30E-05	4
ENSG00000126088	UROD	uroporphyrinogen decarboxylase	P06132	6.70E-05	4
ENSG00000078668	VDAC3	voltage dependent anion channel 3	Q9Y277	1.30E-05	4
ENSG00000126214	KLC1	kinesin light chain 1	Q07866	1.40E-05	3.93
ENSG00000169045	HNRNPH1	heterogeneous nuclear ribonucleoprotein H1	P31943	3.20E-05	3.91
ENSG00000182809	CRIP2	cysteine rich protein 2	P52943	1.10E-04	3.9
ENSG00000117298	ECE1	endothelin converting enzyme 1	A0A024RAB0	1.00E-04	3.9
ENSG00000143321	HDGF	heparin binding growth factor	P51858	2.90E-05	3.89
ENSG00000142634	EFHD2	EF-hand domain family member D2	A0A024QZ77	5.20E-05	3.88
ENSG00000141458	NPC1	NPC intracellular cholesterol transporter 1	O15118	8.70E-05	3.88
ENSG00000108671	PSMD11	proteasome 26S subunit, non-ATPase 11	O00231	1.70E-05	3.88
ENSG00000105971	CAV2	caveolin 2	P51636	7.30E-05	3.87
ENSG00000197956	S100A6	S100 calcium binding protein A6	P06703	0.0015	3.87
ENSG00000197756	RPL37A	ribosomal protein L37a	P61513	0.014	3.85
ENSG00000138119	MYOF	myoferlin	Q9NZM1	5.10E-05	3.84
ENSG00000179091	CYC1	cytochrome c1	P08574	2.60E-05	3.83
ENSG00000203879	GDI1	GDP dissociation inhibitor 1	A0A0S2Z3X8	1.20E-05	3.8
ENSG00000138760	SCARB2	scavenger receptor class B member 2	Q14108	2.00E-05	3.8
ENSG00000178695	KCTD12	potassium channel tetramerization domain containing 12	A0A140VJM4	1.80E-05	3.79
ENSG00000007168	PAFAH1B1	platelet activating factor acetylhydrolase 1b regulatory subunit 1	P43034	4.90E-05	3.78

ENSG00000069345	DNAJA2	DnaJ heat shock protein family (Hsp40) member A2	A0A024R6S1	0.0013	3.75
ENSG00000107796	ACTA2	actin alpha 2, smooth muscle	D2JYH4	8.30E-05	3.74
ENSG00000112306	RPS12	ribosomal protein S12	P25398	1.10E-04	3.74
ENSG00000111737	RAB35	RAB35, member RAS oncogene family	Q15286	3.10E-05	3.7
ENSG00000158467	AHCYL2	adenosylhomocysteinase like 2	Q96HN2	2.60E-04	3.68
ENSG00000159176	CSRP1	cysteine and glycine rich protein 1	B4DY28	7.20E-05	3.68
ENSG00000136937	NCBP1	nuclear cap binding protein subunit 1	A0A024R179	1.20E-04	3.68
ENSG00000159840	ZYX	zyxin	Q15942	3.30E-05	3.68
ENSG00000184584	STING1	stimulator of interferon response cGAMP interactor 1	V5V0K2	2.50E-05	3.67
ENSG00000165637	VDAC2	voltage dependent anion channel 2	P45880	5.40E-05	3.67
ENSG00000070831	CDC42	cell division cycle 42	A0A024RAE4	3.80E-04	3.66
ENSG00000008988	RPS20	ribosomal protein S20	P60866	1.40E-04	3.65
ENSG00000005194	CIAPIN1	cytokine induced apoptosis inhibitor 1	Q6FI81	9.40E-05	3.63
ENSG00000103335	PIEZO1	piezo type mechanosensitive ion channel component 1	Q92508	1.90E-04	3.62
ENSG00000167461	RAB8A	RAB8A, member RAS oncogene family	A0A024R7I3	6.80E-05	3.61
ENSG00000172757	CFL1	cofilin 1	P23528	9.70E-05	3.6
ENSG00000196329	GIMAP5	GTPase, IMAP family member 5	A0A090N8P9	5.40E-05	3.6
ENSG00000013375	PGM3	phosphoglucomutase 3	O95394	1.70E-04	3.6
ENSG00000144746	ARL6IP5	ADP ribosylation factor like GTPase 6 interacting protein 5	A0A024R371	0.0017	3.59
ENSG00000103415	HMOX2	heme oxygenase 2	P30519	1.10E-04	3.59
ENSG00000136160	EDNRB	endothelin receptor type B	P24530	4.20E-05	3.58
ENSG00000169564	PCBP1	poly(rC) binding protein 1	Q15365	9.90E-05	3.57
ENSG00000124570	SERPINB6	serpin family B member 6	A0A024QZX5	1.10E-04	3.57
ENSG00000124767	GLO1	glyoxalase I	Q04760	1.30E-04	3.55
ENSG00000108774	RAB5C	RAB5C, member RAS oncogene family	P51148	4.10E-05	3.55
ENSG00000137962	ARHGAP29	Rho GTPase activating protein 29	Q52LW3	2.30E-05	3.54
ENSG00000164305	CASP3	caspase 3	P42574	3.40E-04	3.53
ENSG00000139726	DENR	density regulated re-initiation and release factor	A0A024RBR3	5.10E-04	3.52
ENSG00000142864	SERBP1	SERPINE1 mRNA binding protein 1	Q8NC51	3.50E-04	3.52
ENSG00000119335	SET	SET nuclear proto-oncogene	Q01105	1.10E-04	3.52
ENSG00000130985	UBA1	ubiquitin like modifier activating enzyme 1	A0A024R1A3	1.10E-04	3.52
ENSG00000108883	EFTUD2	elongation factor Tu GTP binding domain containing 2	B3KX19	5.10E-05	3.49
ENSG00000166128	RAB8B	RAB8B, member RAS oncogene family	Q92930	8.90E-05	3.49
ENSG00000120802	TMPO	thymopoietin	A0A024RBE7	4.30E-04	3.49
ENSG00000136813	ECPAS	Ecm29 proteasome adaptor and scaffold	NA	3.20E-04	3.48
ENSG00000104852	SNRNP70	small nuclear ribonucleoprotein U1 subunit 70	P08621	4.80E-05	3.47
ENSG00000141756	FKBP10	FKBP prolyl isomerase 10	A0A024R1W3	2.60E-04	3.45
ENSG00000067704	IARS2	isoleucyl-tRNA synthetase 2, mitochondrial	Q9NSE4	1.00E-04	3.45

ENSG00000198682	PAPSS2	3'-phosphoadenosine 5'-phosphosulfate synthase 2	O95340	5.30E-05	3.45
ENSG00000158195	WASF2	WASP family member 2	Q9Y6W5	0.0014	3.45
ENSG00000239672	NME1	NME/NM23 nucleoside diphosphate kinase 1	P15531	7.60E-05	3.44
ENSG00000165434	PGM2L1	phosphoglucomutase 2 like 1	Q6PCE3	1.00E-04	3.44
ENSG00000072274	TFRC	transferrin receptor	P02786	2.90E-04	3.44
ENSG00000114942	EEF1B2	eukaryotic translation elongation factor 1 beta 2	A0A024R3W7	1.40E-04	3.43
ENSG00000240972	MIF	macrophage migration inhibitory factor	I4AY87	6.70E-05	3.43
ENSG00000184009	ACTG1	actin gamma 1	P63261	9.70E-05	3.42
ENSG00000135269	TES	testin LIM domain protein	A4D0U5	5.10E-05	3.4
ENSG00000131016	AKAP12	A-kinase anchoring protein 12	Q02952	5.10E-05	3.39
ENSG00000137497	NUMA1	nuclear mitotic apparatus protein 1	A0A024R5M9	1.80E-04	3.39
ENSG00000213719	CLIC1	chloride intracellular channel 1	O00299	8.40E-05	3.37
ENSG00000131143	COX4I1	cytochrome c oxidase subunit 4I1	P13073	5.60E-05	3.37
ENSG00000129562	DAD1	defender against cell death 1	P61803	2.20E-04	3.37
ENSG00000168872	DDX19A	DEAD-box helicase 19A	B4DS24	3.20E-04	3.37
ENSG00000099901	RANBP1	RAN binding protein 1	F6WQW2	4.70E-05	3.36
ENSG00000135318	NT5E	5'-nucleotidase ecto	P21589	0.018	3.35
ENSG00000127314	RAP1B	RAP1B, member of RAS oncogene family	A0A024RB87	1.70E-04	3.35
ENSG00000197894	ADH5	alcohol dehydrogenase 5 (class III), chi polypeptide	P11766	6.10E-05	3.34
ENSG00000149781	FERMT3	FERM domain containing kindlin 3	Q86UX7	1.20E-04	3.34
ENSG00000144959	NCEH1	neutral cholesterol ester hydrolase 1	A0A0A0MTJ9	6.50E-04	3.34
ENSG00000100300	TSPO	translocator protein	O76068	6.00E-05	3.34
ENSG00000101361	NOP56	NOP56 ribonucleoprotein	O00567	6.40E-05	3.33
ENSG00000164828	SUN1	Sad1 and UNC84 domain containing 1	O94901	1.40E-04	3.32
ENSG00000128245	YWHAH	tyrosine 3-monooxygenase/tryptophan 5-monooxygenase activation protein eta	A0A024R1K7	1.20E-04	3.32
ENSG00000104687	GSR	glutathione-disulfide reductase	P00390	2.10E-04	3.31
ENSG00000100664	EIF5	eukaryotic translation initiation factor 5	A0A024R6Q1	0.003	3.3
ENSG00000087470	DNM1L	dynamin 1 like	B4DYR6	1.90E-04	3.29
ENSG00000125691	RPL23	ribosomal protein L23	A0A024R1Q8	0.00093	3.29
ENSG00000087274	ADD1	adducin 1	P35611	1.50E-04	3.28
ENSG00000134748	PRPF38A	pre-mRNA processing factor 38A	Q8NAV1	1.40E-04	3.28
ENSG00000108848	LUC7L3	LUC7 like 3 pre-mRNA splicing factor	J3KPP4	4.80E-05	3.27
ENSG00000151247	EIF4E	eukaryotic translation initiation factor 4E	P06730	0.0033	3.26
ENSG00000114480	GBE1	1,4-alpha-glucan branching enzyme 1	Q04446	1.20E-04	3.26
ENSG00000141543	EIF4A3	eukaryotic translation initiation factor 4A3	A0A024R8W0	1.60E-04	3.25
ENSG00000170027	YWHAG	tyrosine 3-monooxygenase/tryptophan 5-monooxygenase activation protein gamma	P61981	1.70E-04	3.25
ENSG00000185825	BCAP31	B cell receptor associated protein 31	P51572	1.30E-04	3.24
ENSG00000169710	FASN	fatty acid synthase	P49327	9.10E-05	3.24

ENSG00000170348	TMED10	transmembrane p24 trafficking protein 10	A0A024R6I3	2.90E-04	3.24
ENSG00000105568	PPP2R1A	protein phosphatase 2 scaffold subunit Aalpha	A8K7B7	8.50E-05	3.23
ENSG00000010256	UQCRC1	ubiquinol-cytochrome c reductase core protein 1	P31930	6.20E-04	3.23
ENSG00000105974	CAV1	caveolin 1	A0A024R757	4.80E-05	3.22
ENSG00000100519	PSMC6	proteasome 26S subunit, ATPase 6	A0A087X2I1	4.70E-05	3.22
ENSG00000119707	RBM25	RNA binding motif protein 25	P49756	1.70E-04	3.22
ENSG00000100075	SLC25A1	solute carrier family 25 member 1	D9HTE9	6.60E-05	3.22
ENSG00000079785	DDX1	DEAD-box helicase 1	A3RJH1	8.90E-05	3.21
ENSG00000125868	DSTN	destrin, actin depolymerizing factor	P60981	6.50E-04	3.21
ENSG00000100097	LGALS1	galectin 1	P09382	4.50E-04	3.2
ENSG00000126561	STAT5A	signal transducer and activator of transcription 5A	A8K6I5	0.013	3.2
ENSG00000265808	SEC22B	SEC22 homolog B, vesicle trafficking protein	O75396	1.70E-04	3.19
ENSG00000178035	IMPDH2	inosine monophosphate dehydrogenase 2	P12268	5.80E-04	3.18
ENSG00000095139	ARCN1	archain 1	P48444	1.20E-04	3.17
ENSG00000146731	CCT6A	chaperonin containing TCP1 subunit 6A	P40227	2.60E-04	3.17
ENSG00000122545	SEPTIN7	septin 7	A8K3D0	1.80E-04	3.17
ENSG00000135316	SYNCRIP	synaptotagmin binding cytoplasmic RNA interacting protein	B7Z645	3.90E-04	3.17
ENSG00000100934	SEC23A	SEC23 homolog A, COPII coat complex component	Q15436	0.0012	3.16
ENSG00000176014	TUBB6	tubulin beta 6 class V	Q9BUF5	4.30E-04	3.15
ENSG00000055609	KMT2C	lysine methyltransferase 2C	Q8NEZ4	0.0011	3.14
ENSG00000178921	PFAS	phosphoribosylformylglycinamidine synthase	A8K9T9	2.10E-04	3.14
ENSG00000109084	TMEM97	transmembrane protein 97	Q5BJF2	4.10E-04	3.14
ENSG00000134186	PRPF38B	pre-mRNA processing factor 38B	Q5VTL8	6.20E-05	3.13
ENSG00000117592	PRDX6	peroxiredoxin 6	P30041	2.60E-04	3.12
ENSG00000091409	ITGA6	integrin subunit alpha 6	P23229	2.80E-04	3.11
ENSG00000166181	API5	apoptosis inhibitor 5	Q9BZZ5	5.10E-04	3.1
ENSG00000211893	IGHG2	immunoglobulin heavy constant gamma 2 (G2m marker)	NA	0.0021	3.08
ENSG00000111581	NUP107	nucleoporin 107	P57740	0.0037	3.08
ENSG00000175582	RAB6A	RAB6A, member RAS oncogene family	P20340	0.0011	3.08
ENSG00000163636	PSMD6	proteasome 26S subunit, non-ATPase 6	Q15008	3.10E-04	3.07
ENSG00000116754	SRSF11	serine and arginine rich splicing factor 11	Q05519	1.10E-04	3.07
ENSG00000179218	CALR	calreticulin	P27797	1.90E-04	3.06
ENSG00000100030	MAPK1	mitogen-activated protein kinase 1	P28482	1.30E-04	3.06
ENSG00000100813	ACIN1	apoptotic chromatin condensation inducer 1	Q9UKV3	9.50E-05	3.05
ENSG00000146376	ARHGAP18	Rho GTPase activating protein 18	Q8N392	4.40E-04	3.05
ENSG00000125944	HNRNPR	heterogeneous nuclear ribonucleoprotein R	Q0VGD6	3.10E-04	3.04
ENSG00000167553	TUBA1C	tubulin alpha 1c	B7Z1K5	0.022	3.01

ENSG00000130309	COLGALT1	collagen beta(1-O)galactosyltransferase 1	Q8NBJ5	2.40E-04	2.99
ENSG00000062485	CS	citrate synthase	A0A024RB75	0.0014	2.98
ENSG00000143815	LBR	lamin B receptor	Q14739	2.30E-04	2.98
ENSG00000109501	WFS1	wolframin ER transmembrane glycoprotein	A0A0S2Z4V6	1.00E-04	2.98
ENSG00000102898	NUTF2	nuclear transport factor 2	A0A024R6Y2	0.0012	2.97
ENSG00000075624	ACTB	actin beta	P60709	3.30E-04	2.95
ENSG00000134440	NARS1	asparaginyl-tRNA synthetase 1	O43776	1.70E-04	2.95
ENSG00000104325	DECR1	2,4-dienoyl-CoA reductase 1	Q16698	4.70E-04	2.94
ENSG00000169919	GUSB	glucuronidase beta	P08236	1.30E-04	2.93
ENSG00000169504	CLIC4	chloride intracellular channel 4	Q6FIC5	1.90E-04	2.92
ENSG00000172725	CORO1B	coronin 1B	A0A024R5K1	1.20E-04	2.92
ENSG00000070756	PABPC1	poly(A) binding protein cytoplasmic 1	A0A024R9C1	3.80E-04	2.92
ENSG00000130313	PGLS	6-phosphogluconolactonase	A0A0K0K1K7	8.80E-05	2.92
ENSG00000108946	PRKAR1A	protein kinase cAMP-dependent type I regulatory subunit alpha	B2R5T5	0.0031	2.91
ENSG00000132341	RAN	RAN, member RAS oncogene family	B4DV51	0.0012	2.91
ENSG00000100504	PYGL	glycogen phosphorylase L	P06737	0.0037	2.9
ENSG00000022267	FHL1	four and a half LIM domains 1	Q13642	2.20E-04	2.89
ENSG00000176658	MYO1D	myosin ID	J3QRN6	2.20E-04	2.89
ENSG00000213639	PPP1CB	protein phosphatase 1 catalytic subunit beta	P62140	2.90E-04	2.89
ENSG00000092201	SUPT16H	SPT16 homolog, facilitates chromatin remodeling subunit	Q9Y5B9	1.80E-04	2.88
ENSG00000169067	ACTBL2	actin beta like 2	Q562R1	3.90E-04	2.87
ENSG00000074696	HACD3	3-hydroxyacyl-CoA dehydratase 3	Q9P035	2.30E-04	2.87
ENSG00000106397	PLOD3	procollagen-lysine,2-oxoglutarate 5-dioxygenase 3	O60568	3.10E-04	2.87
ENSG00000198363	ASPH	aspartate beta-hydroxylase	Q12797	3.10E-04	2.86
ENSG00000133318	RTN3	reticulon 3	O95197	1.90E-04	2.86
ENSG00000171560	FGA	fibrinogen alpha chain	P02671	0.013	2.84
ENSG00000132507	EIF5A	eukaryotic translation initiation factor 5A	P63241	2.70E-04	2.83
ENSG00000169738	DCXR	dicarbonyl and L-xylulose reductase	Q7Z4W1	4.70E-04	2.81
ENSG00000197111	PCBP2	poly(rC) binding protein 2	Q15366	3.40E-04	2.81
ENSG00000101558	VAPA	VAMP associated protein A	Q9P0L0	3.80E-04	2.81
ENSG00000130429	ARPC1B	actin related protein 2/3 complex subunit 1B	A4D275	2.70E-04	2.8
ENSG00000198668	CALM1	calmodulin 1	B4DJ51	6.00E-04	2.8
ENSG00000075856	SART3	spliceosome associated factor 3, U4/U6 recycling protein	Q15020	0.0099	2.79
ENSG00000138772	ANXA3	annexin A3	P12429	1.50E-04	2.77
ENSG00000150593	PDCD4	programmed cell death 4	B4DKX4	4.50E-04	2.77
ENSG00000239264	TXNDC5	thioredoxin domain containing 5	A0A024QZV0	2.90E-04	2.77
ENSG00000072778	ACADVL	acyl-CoA dehydrogenase very long chain	P49748	4.20E-04	2.76
ENSG00000154473	BUB3	BUB3 mitotic checkpoint protein	A0A140VJF3	0.0011	2.75
ENSG00000143621	ILF2	interleukin enhancer binding factor 2	B4DY09	4.10E-04	2.74
ENSG00000103642	LACTB	lactamase beta	P83111	0.0022	2.74

ENSG00000167004	PDIA3	protein disulfide isomerase family A member 3	P30101	4.20E-04	2.74
ENSG00000157020	SEC13	SEC13 homolog, nuclear pore and COPII coat complex component	P55735	5.90E-04	2.74
ENSG00000090273	NUDC	nuclear distribution C, dynein complex regulator	Q9Y266	0.0013	2.73
ENSG00000197702	PARVA	parvin alpha	J3KNQ4	0.0021	2.73
ENSG00000167699	GLOD4	glyoxalase domain containing 4	Q9HC38	2.00E-04	2.72
ENSG00000083845	RPS5	ribosomal protein S5	A0A024R4Q8	5.30E-04	2.72
ENSG00000149089	APIP	APAF1 interacting protein	Q96GX9	0.0014	2.71
ENSG00000158710	TAGLN2	transgelin 2	P37802	0.0033	2.71
ENSG00000122958	VPS26A	VPS26 retromer complex component A	O75436	0.01	2.71
ENSG00000169021	UQCRFS1	ubiquinol-cytochrome c reductase, Rieske iron-sulfur polypeptide 1	P47985	0.0017	2.7
ENSG00000130396	AFDN	afadin, adherens junction formation factor	P55196	0.0022	2.69
ENSG00000177731	FLII	FLII actin remodeling protein	Q13045	4.60E-04	2.69
ENSG00000120254	MTHFD1L	methylenetetrahydrofolate dehydrogenase (NADP+ dependent) 1 like	B7ZM99	1.60E-04	2.69
ENSG00000125977	EIF2S2	eukaryotic translation initiation factor 2 subunit beta	P20042	0.0011	2.68
ENSG00000067113	PLPP1	phospholipid phosphatase 1	A0A024QZS3	0.0028	2.68
ENSG00000168003	SLC3A2	solute carrier family 3 member 2	J3KPF3	2.90E-04	2.68
ENSG00000166747	AP1G1	adaptor related protein complex 1 subunit gamma 1	A0A140VJE7	3.70E-04	2.65
ENSG00000143819	EPHX1	epoxide hydrolase 1	P07099	9.70E-04	2.65
ENSG00000105379	ETFB	electron transfer flavoprotein subunit beta	P38117	6.10E-04	2.65
ENSG00000119396	RAB14	RAB14, member RAS oncogene family	A0A024R845	0.0071	2.65
ENSG00000140740	UQCRC2	ubiquinol-cytochrome c reductase core protein 2	P22695	0.0031	2.65
ENSG00000151693	ASAP2	ArfGAP with SH3 domain, ankyrin repeat and PH domain 2	O43150	5.30E-04	2.64
ENSG00000108094	CUL2	cullin 2	A0A140VKB1	0.035	2.64
ENSG00000154174	TOMM70	translocase of outer mitochondrial membrane 70	O94826	0.0014	2.64
ENSG00000091164	TXNL1	thioredoxin like 1	O43396	0.0012	2.64
ENSG00000164022	AIMP1	aminoacyl tRNA synthetase complex interacting multifunctional protein 1	B4DNK3	5.60E-04	2.63
ENSG00000148841	ITPRIP	inositol 1,4,5-trisphosphate receptor interacting protein	Q8IWB1	3.20E-04	2.63
ENSG00000099246	RAB18	RAB18, member RAS oncogene family	Q9NP72	6.00E-04	2.63
ENSG00000132842	AP3B1	adaptor related protein complex 3 subunit beta 1	A0A0S2Z5J4	4.20E-04	2.6
ENSG00000138777	PPA2	inorganic pyrophosphatase 2	Q9H2U2	0.0024	2.6
ENSG00000084652	TXLNA	taxilin alpha	P40222	3.70E-04	2.6
ENSG00000145833	DDX46	DEAD-box helicase 46	A0A0C4DG89	9.00E-04	2.59
ENSG00000118257	NRP2	neuropilin 2	O60462	3.20E-04	2.59
ENSG00000100911	PSME2	proteasome activator subunit 2	Q86SZ7	9.20E-04	2.59
ENSG00000169398	PTK2	protein tyrosine kinase 2	Q05397	3.30E-04	2.59
ENSG00000137710	RDX	radixin	P35241	2.40E-04	2.58

ENSG00000073969	NSF	N-ethylmaleimide sensitive factor, vesicle fusing ATPase	P46459	2.70E-04	2.57
ENSG00000089157	RPLP0	ribosomal protein lateral stalk subunit P0	A0A024RBS2	3.40E-04	2.57
ENSG00000147140	NONO	non-POU domain containing octamer binding	A0A0S2Z4Z9	0.0043	2.55
ENSG00000031698	SARS1	seryl-tRNA synthetase 1	Q5T5C7	8.10E-04	2.54
ENSG00000170876	TMEM43	transmembrane protein 43	A0A024R2F9	0.0023	2.54
ENSG00000107862	GBF1	golgi brefeldin A resistant guanine nucleotide exchange factor 1	Q92538	4.50E-04	2.53
ENSG00000067560	RHOA	ras homolog family member A	A0A024R324	0.011	2.53
ENSG00000163191	S100A11	S100 calcium binding protein A11	P31949	0.0022	2.53
ENSG00000115233	PSMD14	proteasome 26S subunit, non-ATPase 14	A0A140VKF2	0.003	2.52
ENSG00000136450	SRSF1	serine and arginine rich splicing factor 1	Q07955	0.0082	2.52
ENSG00000188229	TUBB4B	tubulin beta 4B class IVb	P68371	9.50E-04	2.52
ENSG00000134308	YWHAQ	tyrosine 3-monooxygenase/tryptophan 5-monooxygenase activation protein theta	P27348	6.60E-04	2.52
ENSG00000148700	ADD3	adducin 3	Q9UEY8	3.80E-04	2.5
ENSG00000168374	ARF4	ADP ribosylation factor 4	P18085	0.0018	2.5
ENSG00000125107	CNOT1	CCR4-NOT transcription complex subunit 1	A5YKK6	0.0065	2.5
ENSG00000197728	RPS26	ribosomal protein S26	A0A024RB14	0.013	2.5
ENSG00000165092	ALDH1A1	aldehyde dehydrogenase 1 family member A1	P00352	7.80E-04	2.49
ENSG00000065485	PDIA5	protein disulfide isomerase family A member 5	Q14554	0.0011	2.49
ENSG00000187514	PTMA	prothymosin alpha	P06454	0.015	2.49
ENSG00000177105	RHOG	ras homolog family member G	P84095	0.0016	2.47
ENSG00000162695	SLC30A7	solute carrier family 30 member 7	Q8NEW0	8.60E-04	2.47
ENSG00000142192	APP	amyloid beta precursor protein	A0A140VJC8	0.0035	2.46
ENSG00000162616	DNAJB4	DnaJ heat shock protein family (Hsp40) member B4	Q9UDY4	6.20E-04	2.46
ENSG00000197892	KIF13B	kinesin family member 13B	Q9NQT8	0.0038	2.46
ENSG00000168259	DNAJC7	DnaJ heat shock protein family (Hsp40) member C7	Q99615	0.0011	2.45
ENSG00000176619	LMNB2	lamin B2	Q03252	6.80E-04	2.45
ENSG00000130726	TRIM28	tripartite motif containing 28	Q13263	0.0035	2.45
ENSG00000113810	SMC4	structural maintenance of chromosomes 4	Q58F29	8.30E-04	2.44
ENSG00000163931	TKT	transketolase	P29401	4.40E-04	2.44
ENSG00000101150	TPD52L2	TPD52 like 2	O43399	4.80E-04	2.44
ENSG00000100243	CYB5R3	cytochrome b5 reductase 3	P00387	6.50E-04	2.42
ENSG00000137509	PRCP	prolylcarboxypeptidase	B7Z7Q6	0.008	2.42
ENSG00000054118	THRAP3	thyroid hormone receptor associated protein 3	Q9Y2W1	0.013	2.42
ENSG00000140350	ANP32A	acidic nuclear phosphoprotein 32 family member A	P39687	0.0012	2.41
ENSG00000166825	ANPEP	alanyl aminopeptidase, membrane	A0A024RC61	0.0042	2.41
ENSG00000057757	PITHD1	PITH domain containing 1	Q9GZP4	6.40E-04	2.41
ENSG00000101474	APMAP	adipocyte plasma membrane associated protein	Q9HDC9	0.0022	2.39

ENSG00000273703	H2BC14	H2B clustered histone 14	Q99879	0.044	2.39
ENSG00000141959	PFKL	phosphofructokinase, liver type	P17858	9.40E-04	2.39
ENSG00000133226	SRRM1	serine and arginine repetitive matrix 1	B7Z7U0	6.10E-04	2.39
ENSG00000085063	CD59	CD59 molecule (CD59 blood group)	P13987	0.026	2.38
ENSG00000166333	ILK	integrin linked kinase	Q13418	0.0045	2.38
ENSG00000112245	PTP4A1	protein tyrosine phosphatase 4A1	A0A024R8J2	0.019	2.38
ENSG00000075415	SLC25A3	solute carrier family 25 member 3	A0A024RBE8	5.80E-04	2.38
ENSG00000142937	RPS8	ribosomal protein S8	P62241	8.40E-04	2.37
ENSG00000143549	TPM3	tropomyosin 3	A0A0S2Z4I4	5.90E-04	2.37
ENSG00000102401	ARMCX3	armadillo repeat containing X-linked 3	A0A024RCF9	7.30E-04	2.36
ENSG00000241553	ARPC4	actin related protein 2/3 complex subunit 4	P59998	0.0024	2.36
ENSG00000133112	TPT1	tumor protein, translationally-controlled 1	A0A0B4J2C3	0.026	2.36
ENSG00000153904	DDAH1	dimethylarginine dimethylaminohydrolase 1	B4E3V1	0.0011	2.35
ENSG00000104408	EIF3E	eukaryotic translation initiation factor 3 subunit E	P60228	5.20E-04	2.35
ENSG00000169756	LIMS1	LIM zinc finger domain containing 1	P48059	6.90E-04	2.35
ENSG00000147274	RBMX	RNA binding motif protein X-linked	P38159	0.0013	2.35
ENSG00000197958	RPL12	ribosomal protein L12	P30050	4.30E-04	2.35
ENSG00000079332	SAR1A	secretion associated Ras related GTPase 1A	Q5SQT9	0.0029	2.35
ENSG00000028528	SNX1	sorting nexin 1	Q13596	8.30E-04	2.35
ENSG00000115380	EFEMP1	EGF containing fibulin extracellular matrix protein 1	A0A0S2Z4F1	0.0015	2.34
ENSG00000132780	NASP	nuclear autoantigenic sperm protein	P49321	9.10E-04	2.34
ENSG00000167792	NDUFV1	NADH:ubiquinone oxidoreductase core subunit V1	P49821	9.70E-04	2.34
ENSG00000075618	FSCN1	fascin actin-bundling protein 1	B3KTA3	0.002	2.33
ENSG00000103342	GSPT1	G1 to S phase transition 1	P15170	5.40E-04	2.32
ENSG00000092841	MYL6	myosin light chain 6	P60660	0.0016	2.32
ENSG00000023228	NDUFS1	NADH:ubiquinone oxidoreductase core subunit S1	P28331	0.0035	2.32
ENSG00000076043	REXO2	RNA exonuclease 2	Q9Y3B8	0.0011	2.32
ENSG00000072501	SMC1A	structural maintenance of chromosomes 1A	G8JLG1	0.0014	2.32
ENSG00000148834	GSTO1	glutathione S-transferase omega 1	P78417	7.10E-04	2.31
ENSG00000171863	RPS7	ribosomal protein S7	P62081	0.0021	2.31
ENSG00000127022	CANX	calnexin	P27824	5.60E-04	2.3
ENSG00000139684	ESD	esterase D	A0A140VJJ2	0.0026	2.3
ENSG00000167770	OTUB1	OTU deubiquitinase, ubiquitin aldehyde binding 1	B3KUV5	5.70E-04	2.3
ENSG00000166340	TPP1	tripeptidyl peptidase 1	O14773	0.0046	2.3
ENSG00000185624	P4HB	prolyl 4-hydroxylase subunit beta	A0A024R8S5	0.004	2.29
ENSG00000241973	PI4KA	phosphatidylinositol 4-kinase alpha	B4DYG5	0.0077	2.29

Adjustments of p-values for multiple comparisons were used with Benjamini-Hochberg (BH) correction.

Supplemental Table S5. Palmitoylated proteins in insulin treated siCD36 LECs.

ENSEMBL ID	Gene Symbol	Description	UNIPROT	*p*-value	-Log Ratio (+/- HA)
ENSG00000213699	SLC35F6	solute carrier family 35 member F6	Q8N357	0.00000017	7.76
ENSG00000002586	CD99	CD99 molecule (Xg blood group)	P14209	0.00000014	7.02
ENSG00000054116	TRAPPC3	trafficking protein particle complex subunit 3	A0A087WWM0	0.00000041	6.82
ENSG00000111897	SERINC1	serine incorporator 1	Q9NRX5	0.00000036	6.71
ENSG00000102007	PLP2	proteolipid protein 2	A0A024QYW3	0.0000021	6.67
ENSG00000136156	ITM2B	integral membrane protein 2B	Q9Y287	0.00000029	6.63
ENSG00000110651	CD81	CD81 molecule	E9PJK1	0.00000032	6.49
ENSG00000177697	CD151	CD151 molecule (Raph blood group)	A0A024RCB3	0.0000012	6.41
ENSG00000076706	MCAM	melanoma cell adhesion molecule	A0A024R3I5	0.00000056	6.27
ENSG00000143878	RHOB	ras homolog family member B	P62745	0.0000016	6.24
ENSG00000139921	TMX1	thioredoxin related transmembrane protein 1	Q9H3N1	0.00000047	6.08
ENSG00000132388	UBE2G1	ubiquitin conjugating enzyme E2 G1	P62253	0.00000052	6.03
ENSG00000049245	VAMP3	vesicle associated membrane protein 3	Q15836	0.00000085	6.02
ENSG00000114698	PLSCR4	phospholipid scramblase 4	Q9NRQ2	0.00000099	6.02
ENSG00000135404	CD63	CD63 molecule	A0A024RB05	0.0000058	6.01
ENSG00000092531	SNAP23	synaptosome associated protein 23	A8K287	0.00000044	5.99
ENSG00000003056	M6PR	mannose-6-phosphate receptor, cation dependent	F5GX30	0.00000081	5.96
ENSG00000133818	RRAS2	RAS related 2	P62070	0.0000011	5.96
ENSG00000185651	UBE2L3	ubiquitin conjugating enzyme E2 L3	P68036	0.0000049	5.9
ENSG00000144744	UBA3	ubiquitin like modifier activating enzyme 3	Q8TBC4	0.00000072	5.86
ENSG00000110917	MLEC	malectin	F5GX14	0.00000084	5.76
ENSG00000187838	PLSCR3	phospholipid scramblase 3	Q9NRY6	0.0000024	5.76
ENSG00000143222	UFC1	ubiquitin-fold modifier conjugating enzyme 1	Q9Y3C8	0.000012	5.74
ENSG00000072778	ACADVL	acyl-CoA dehydrogenase very long chain	P49748	0.0000019	5.71
ENSG00000184113	CLDN5	claudin 5	D3DX19	0.0000034	5.67
ENSG00000169908	TM4SF1	transmembrane 4 L six family member 1	P30408	0.00000083	5.59
ENSG00000130725	UBE2M	ubiquitin conjugating enzyme E2 M	A0A024R4T4	0.0000027	5.59
ENSG00000148175	STOM	stomatin	F8VSL7	0.0000016	5.58
ENSG00000129625	REEP5	receptor accessory protein 5	Q00765	0.0000016	5.54
ENSG00000227500	SCAMP4	secretory carrier membrane protein 4	Q969E2	0.0000014	5.44
ENSG00000033178	UBA6	ubiquitin like modifier activating enzyme 6	A0A024RDB0	0.0000013	5.38

ENSG00000005194	CIAPIN1	cytokine induced apoptosis inhibitor 1	Q6FI81	0.0000049	5.37
ENSG00000141543	EIF4A3	eukaryotic translation initiation factor 4A3	A0A024R8W0	0.0000039	5.35
ENSG00000142089	IFITM3	interferon induced transmembrane protein 3	Q01628	0.0000026	5.33
ENSG00000143079	CTTNBP2NL	CTTNBP2 N-terminal like	A0A024R0C7	0.000024	5.32
ENSG00000108219	TSPAN14	tetraspanin 14	Q8NG11	0.0000044	5.31
ENSG00000168899	VAMP5	vesicle associated membrane protein 5	O95183	0.0000043	5.28
ENSG00000116521	SCAMP3	secretory carrier membrane protein 3	O14828	0.0000023	5.21
ENSG00000183726	TMEM50A	transmembrane protein 50A	O95807	0.0000051	5.11
ENSG00000168374	ARF4	ADP ribosylation factor 4	P18085	0.000012	5.11
ENSG00000169756	LIMS1	LIM zinc finger domain containing 1	P48059	0.0000024	5.1
ENSG00000129353	SLC44A2	solute carrier family 44 member 2	A0A088QCU6	0.000015	5.08
ENSG00000132589	FLOT2	flotillin 2	J3QLD9	0.0000097	5.07
ENSG00000204525	HLA-C	major histocompatibility complex, class I, C	O19617	0.000024	5.05
ENSG00000037280	FLT4	fms related receptor tyrosine kinase 4	P35916	0.0000051	5.02
ENSG00000115310	RTN4	reticulon 4	Q9NQC3	0.000011	4.93
ENSG00000243279	PRAF2	PRA1 domain family member 2	A0A024QZ22	0.00001	4.91
ENSG00000010278	CD9	CD9 molecule	A6NNI4	0.0000025	4.9
ENSG00000204632	HLA-G	major histocompatibility complex, class I, G	P17693	0.0000084	4.88
ENSG00000249751	ECSCR	endothelial cell surface expressed chemotaxis and apoptosis regulator	Q19T08	0.0000087	4.88
ENSG00000113811	SELENOK	selenoprotein K	Q9Y6D0	0.0000067	4.87
ENSG00000141458	NPC1	NPC intracellular cholesterol transporter 1	O15118	0.000016	4.86
ENSG00000099282	TSPAN15	tetraspanin 15	O95858	0.000027	4.86
ENSG00000134247	PTGFRN	prostaglandin F2 receptor inhibitor	Q9P2B2	0.00001	4.83
ENSG00000006451	RALA	RAS like proto-oncogene A	P11233	0.0000079	4.82
ENSG00000078140	UBE2K	ubiquitin conjugating enzyme E2 K	P61086	0.0000045	4.79
ENSG00000105379	ETFB	electron transfer flavoprotein subunit beta	P38117	0.0000096	4.72
ENSG00000168515	SCGB1D1	secretoglobin family 1D member 1	O95968	0.0019	4.69
ENSG00000099864	PALM	paralemmin	A0A024R207	0.00001	4.66
ENSG00000114115	RBP1	retinol binding protein 1	P09455	0.0000054	4.65
ENSG00000188313	PLSCR1	phospholipid scramblase 1	O15162	0.000024	4.65
ENSG00000122378	PRXL2A	peroxiredoxin like 2A	Q9BRX8	0.0000069	4.62
ENSG00000177889	UBE2N	ubiquitin conjugating enzyme E2 N	P61088	0.0000034	4.6
ENSG00000087460	GNAS	GNAS complex locus	A0A0S2Z3H8	0.00002	4.59
ENSG00000143815	LBR	lamin B receptor	Q14739	0.00001	4.56
ENSG00000133318	RTN3	reticulon 3	O95197	0.0000063	4.54

ENSG00000163762	TM4SF18	transmembrane 4 L six family member 18	Q96CE8	0.000034	4.49
ENSG00000114353	GNAI2	G protein subunit alpha i2	B3KP24	0.0000097	4.43
ENSG00000178035	IMPDH2	inosine monophosphate dehydrogenase 2	P12268	0.000058	4.42
ENSG00000118873	RAB3GAP2	RAB3 GTPase activating non-catalytic protein subunit 2	Q9H2M9	0.0000069	4.39
ENSG00000233276	GPX1	glutathione peroxidase 1	P07203	0.00001	4.38
ENSG00000144118	RALB	RAS like proto-oncogene B	A0A024RAG3	0.0000088	4.36
ENSG00000135047	CTSL	cathepsin L	A0A024R276	0.0000078	4.34
ENSG00000144848	ATG3	autophagy related 3	Q9NT62	0.000011	4.27
ENSG00000213281	NRAS	NRAS proto-oncogene, GTPase	P01111	0.000051	4.16
ENSG00000168172	HOOK3	hook microtubule tethering protein 3	Q86VS8	0.00085	4.16
ENSG00000198431	TXNRD1	thioredoxin reductase 1	Q16881	0.000017	4.15
ENSG00000141526	SLC16A3	solute carrier family 16 member 3	A0A024R8U1	0.000011	4.12
ENSG00000167996	FTH1	ferritin heavy chain 1	A0A024R525	0.00004	4.1
ENSG00000129562	DAD1	defender against cell death 1	P61803	0.000055	4.09
ENSG00000120437	ACAT2	acetyl-CoA acetyltransferase 2	Q9BWD1	0.000048	4.03
ENSG00000101160	CTSZ	cathepsin Z	Q9UBR2	0.000051	4.01
ENSG00000182809	CRIP2	cysteine rich protein 2	P52943	0.000091	4
ENSG00000197956	S100A6	S100 calcium binding protein A6	P06703	0.0013	3.97
ENSG00000109133	TMEM33	transmembrane protein 33	A0A024R9W7	0.000043	3.95
ENSG00000117298	ECE1	endothelin converting enzyme 1	A0A024RAB0	0.00012	3.83
ENSG00000072274	TFRC	transferrin receptor	P02786	0.00014	3.83
ENSG00000152661	GJA1	gap junction protein alpha 1	P17302	0.00014	3.81
ENSG00000052749	RRP12	ribosomal RNA processing 12 homolog	B3KMR5	0.000047	3.77
ENSG00000197747	S100A10	S100 calcium binding protein A10	P60903	0.00012	3.75
ENSG00000066322	ELOVL1	ELOVL fatty acid elongase 1	Q9BW60	0.000046	3.73
ENSG00000107796	ACTA2	actin alpha 2, smooth muscle	D2JYH4	0.000087	3.71
ENSG00000078668	VDAC3	voltage dependent anion channel 3	Q9Y277	0.000024	3.7
ENSG00000164022	AIMP1	aminoacyl tRNA synthetase complex interacting multifunctional protein 1	B4DNK3	0.000051	3.69
ENSG00000126214	KLC1	kinesin light chain 1	Q07866	0.000023	3.68
ENSG00000138760	SCARB2	scavenger receptor class B member 2	Q14108	0.000026	3.68
ENSG00000104131	EIF3J	eukaryotic translation initiation factor 3 subunit J	O75822	0.000059	3.68
ENSG00000125868	DSTN	destrin, actin depolymerizing factor	P60981	0.00026	3.68
ENSG00000160285	LSS	lanosterol synthase	B2R694	0.000021	3.63
ENSG00000166747	AP1G1	adaptor related protein complex 1 subunit gamma 1	A0A140VJE7	0.00004	3.62
ENSG00000184575	XPOT	exportin for tRNA	O43592	0.000033	3.6
ENSG00000114480	GBE1	1,4-alpha-glucan branching enzyme 1	Q04446	0.000059	3.6

ENSG00000115419	GLS	glutaminase	O94925	0.00014	3.6
ENSG00000101367	MAPRE1	microtubule associated protein RP/EB family member 1	Q15691	0.00003	3.58
ENSG00000136026	CKAP4	cytoskeleton associated protein 4	A0A024RBH2	0.000074	3.58
NA	5MP2_HUMAN	NA	NA	0.00081	3.56
ENSG00000135269	TES	testin LIM domain protein	A4D0U5	0.000038	3.54
ENSG00000262246	CORO7	coronin 7	P57737	0.00018	3.54
ENSG00000151914	DST	dystonin	B4DSS9	0.0021	3.52
ENSG00000112531	QKI	QKI, KH domain containing RNA binding	Q96PU8	0.000027	3.46
ENSG00000197249	SERPINA1	serpin family A member 1	E9KL23	0.0064	3.45
ENSG00000163565	IFI16	interferon gamma inducible protein 16	Q16666	0.00024	3.43
ENSG00000179091	CYC1	cytochrome c1	P08574	0.00006	3.42
ENSG00000106636	YKT6	YKT6 v-SNARE homolog	A4D2J0	0.00026	3.42
ENSG00000057757	PITHD1	PITH domain containing 1	Q9GZP4	0.000058	3.4
ENSG00000143549	TPM3	tropomyosin 3	A0A0S2Z4I4	0.000048	3.38
ENSG00000132470	ITGB4	integrin subunit beta 4	A0A024R8T0	0.000088	3.37
ENSG00000066056	TIE1	tyrosine kinase with immunoglobulin like and EGF like domains 1	B4DTW8	0.000041	3.33
ENSG00000179051	RCC2	regulator of chromosome condensation 2	A0A024RAC5	0.00022	3.3
ENSG00000157916	RER1	retention in endoplasmic reticulum sorting receptor 1	O15258	0.00026	3.3
ENSG00000147155	EBP	EBP cholestenol delta-isomerase	A0A024QYX0	0.00033	3.3
ENSG00000022267	FHL1	four and a half LIM domains 1	Q13642	0.000089	3.29
ENSG00000110841	PPFIBP1	PPFIA binding protein 1	A0A024RB02	0.00042	3.28
ENSG00000112739	PRPF4B	pre-mRNA processing factor 4B	A0A024QZY5	0.000062	3.27
ENSG00000153904	DDAH1	dimethylarginine dimethylaminohydrolase 1	B4E3V1	0.00013	3.26
ENSG00000169067	ACTBL2	actin beta like 2	Q562R1	0.00016	3.25
ENSG00000105971	CAV2	caveolin 2	P51636	0.00025	3.25
ENSG00000110619	CARS1	cysteinyl-tRNA synthetase 1	P49589	0.00025	3.22
ENSG00000114416	FXR1	FMR1 autosomal homolog 1	P51114	0.000074	3.21
ENSG00000017260	ATP2C1	ATPase secretory pathway Ca2+ transporting 1	P98194	0.000057	3.2
ENSG00000139684	ESD	esterase D	A0A140VJJ2	0.00033	3.18
ENSG00000134001	EIF2S1	eukaryotic translation initiation factor 2 subunit alpha	P05198	0.0004	3.18
ENSG00000100519	PSMC6	proteasome 26S subunit, ATPase 6	A0A087X2I1	0.000054	3.17
ENSG00000100030	MAPK1	mitogen-activated protein kinase 1	P28482	0.000098	3.17
ENSG00000103342	GSPT1	G1 to S phase transition 1	P15170	0.000061	3.16
ENSG00000130985	UBA1	ubiquitin like modifier activating enzyme 1	A0A024R1A3	0.00023	3.16
ENSG00000112306	RPS12	ribosomal protein S12	P25398	0.00036	3.15
ENSG00000135048	CEMIP2	cell migration inducing hyaluronidase 2	Q9UHN6	0.000067	3.11

ENSG00000138029	HADHB	hydroxyacyl-CoA dehydrogenase trifunctional multienzyme complex subunit beta	P55084	0.000086	3.1
ENSG00000008988	RPS20	ribosomal protein S20	P60866	0.00045	3.1
ENSG00000172725	CORO1B	coronin 1B	A0A024R5K1	0.000078	3.09
ENSG00000184584	STING1	stimulator of interferon response cGAMP interactor 1	V5V0K2	0.000093	3.08
ENSG00000138119	MYOF	myoferlin	Q9NZM1	0.00028	3.01
ENSG00000231500	RPS18	ribosomal protein S18	P62269	0.00014	3
ENSG00000111275	ALDH2	aldehyde dehydrogenase 2 family member	P05091	0.000094	2.98
ENSG00000185825	BCAP31	B cell receptor associated protein 31	P51572	0.00024	2.98
ENSG00000163110	PDLIM5	PDZ and LIM domain 5	Q96HC4	0.00024	2.97
ENSG00000159840	ZYX	zyxin	Q15942	0.00016	2.96
ENSG00000087365	SF3B2	splicing factor 3b subunit 2	Q13435	0.00028	2.96
ENSG00000103018	CYB5B	cytochrome b5 type B	J3KNF8	0.00063	2.96
ENSG00000211899	IGHM	immunoglobulin heavy constant mu	NA	0.0051	2.95
ENSG00000075624	ACTB	actin beta	P60709	0.00035	2.93
ENSG00000172354	GNB2	G protein subunit beta 2	P62879	0.00092	2.93
ENSG00000137486	ARRB1	arrestin beta 1	B7Z1Q3	0.0006	2.92
ENSG00000124181	PLCG1	phospholipase C gamma 1	P19174	0.0018	2.92
ENSG00000101361	NOP56	NOP56 ribonucleoprotein	O00567	0.00017	2.9
ENSG00000067560	RHOA	ras homolog family member A	A0A024R324	0.0052	2.9
ENSG00000060237	WNK1	WNK lysine deficient protein kinase 1	Q9H4A3	0.0063	2.9
ENSG00000100823	APEX1	apurinic/apyrimidinic endodeoxyribonuclease 1	P27695	0.00044	2.89
ENSG00000110047	EHD1	EH domain containing 1	B2R5U3	0.0001	2.87
ENSG00000126261	UBA2	ubiquitin like modifier activating enzyme 2	Q9UBT2	0.0002	2.87
ENSG00000125970	RALY	RALY heterogeneous nuclear ribonucleoprotein	Q9UKM9	0.0068	2.87
ENSG00000105974	CAV1	caveolin 1	A0A024R757	0.00011	2.86
ENSG00000150768	DLAT	dihydrolipoamide S-acetyltransferase	P10515	0.00044	2.86
ENSG00000149218	ENDOD1	endonuclease domain containing 1	O94919	0.0011	2.86
ENSG00000214078	CPNE1	copine 1	B0QZ18	0.004	2.86
ENSG00000048828	FAM120A	family with sequence similarity 120 member A	Q9NZB2	0.0011	2.84
ENSG00000062822	POLD1	DNA polymerase delta 1, catalytic subunit	A0A024R4F4	0.0012	2.82
ENSG00000115286	NDUFS7	NADH:ubiquinone oxidoreductase core subunit S7	O75251	0.00027	2.81
ENSG00000091879	ANGPT2	angiopoietin 2	O15123	0.00048	2.81
ENSG00000178695	KCTD12	potassium channel tetramerization domain containing 12	A0A140VJM4	0.00017	2.8
ENSG00000107438	PDLIM1	PDZ and LIM domain 1	O00151	0.00044	2.8
ENSG00000142634	EFHD2	EF-hand domain family member D2	A0A024QZ77	0.00053	2.8

ENSG00000142676	RPL11	ribosomal protein L11	P62913	0.0017	2.8
ENSG00000104419	NDRG1	N-myc downstream regulated 1	Q92597	0.00082	2.79
ENSG00000175931	UBE2O	ubiquitin conjugating enzyme E2 O	Q9C0C9	0.00015	2.78
ENSG00000141522	ARHGDIA	Rho GDP dissociation inhibitor alpha	P52565	0.00059	2.78
ENSG00000117054	ACADM	acyl-CoA dehydrogenase medium chain	A0A0S2Z366	0.00071	2.77
ENSG00000113441	LNPEP	leucyl and cystinyl aminopeptidase	Q9UIQ6	0.00018	2.76
ENSG00000171314	PGAM1	phosphoglycerate mutase 1	B7Z9E5	0.00014	2.75
ENSG00000244687	UBE2V1	ubiquitin conjugating enzyme E2 V1	Q13404	0.00023	2.75
ENSG00000241973	PI4KA	phosphatidylinositol 4-kinase alpha	B4DYG5	0.0028	2.74
ENSG00000132507	EIF5A	eukaryotic translation initiation factor 5A	P63241	0.00036	2.72
ENSG00000196923	PDLIM7	PDZ and LIM domain 7	Q9NR12	0.00057	2.72
ENSG00000117592	PRDX6	peroxiredoxin 6	P30041	0.00066	2.72
ENSG00000087274	ADD1	adducin 1	P35611	0.00055	2.71
ENSG00000122406	RPL5	ribosomal protein L5	A2RUM7	0.00023	2.7
ENSG00000082516	GEMIN5	gem nuclear organelle associated protein 5	B7ZLC9	0.00024	2.7
ENSG00000092841	MYL6	myosin light chain 6	P60660	0.00059	2.7
ENSG00000167792	NDUFV1	NADH:ubiquinone oxidoreductase core subunit V1	P49821	0.00039	2.68
ENSG00000116251	RPL22	ribosomal protein L22	P35268	0.00049	2.68
ENSG00000186642	PDE2A	phosphodiesterase 2A	O00408	0.00046	2.66
ENSG00000111737	RAB35	RAB35, member RAS oncogene family	Q15286	0.00035	2.64
ENSG00000150593	PDCD4	programmed cell death 4	B4DKX4	0.00062	2.64
ENSG00000100220	RTCB	RNA 2',3'-cyclic phosphate and 5'-OH ligase	Q9Y3I0	0.00041	2.63
ENSG00000104408	EIF3E	eukaryotic translation initiation factor 3 subunit E	P60228	0.00025	2.62
ENSG00000158467	AHCYL2	adenosylhomocysteinase like 2	Q96HN2	0.0023	2.62
ENSG00000102898	NUTF2	nuclear transport factor 2	A0A024R6Y2	0.0034	2.62
ENSG00000165609	NUDT5	nudix hydrolase 5	Q9UKK9	0.00038	2.61
ENSG00000105323	HNRNPUL1	heterogeneous nuclear ribonucleoprotein U like 1	B7Z4B8	0.00046	2.61
ENSG00000164163	ABCE1	ATP binding cassette subfamily E member 1	P61221	0.0006	2.61
ENSG00000023228	NDUFS1	NADH:ubiquinone oxidoreductase core subunit S1	P28331	0.0017	2.61
ENSG00000128245	YWHAH	tyrosine 3-monooxygenase/tryptophan 5-monooxygenase activation protein eta	A0A024R1K7	0.00063	2.6
ENSG00000184009	ACTG1	actin gamma 1	P63261	0.00064	2.6
ENSG00000172757	CFL1	cofilin 1	P23528	0.0009	2.6
ENSG00000130741	EIF2S3	eukaryotic translation initiation factor 2 subunit gamma	P41091	0.0014	2.6
ENSG00000108848	LUC7L3	LUC7 like 3 pre-mRNA splicing factor	J3KPP4	0.00025	2.59
ENSG00000011485	PPP5C	protein phosphatase 5 catalytic subunit	A0A024R0Q7	0.00024	2.58

ENSG00000140350	ANP32A	acidic nuclear phosphoprotein 32 family member A	P39687	0.00078	2.58
ENSG00000141429	GALNT1	polypeptide N-acetylgalactosaminyltransferase 1	A0A024RC48	0.0011	2.58
ENSG00000115091	ACTR3	actin related protein 3	B4DXW1	0.0021	2.58
ENSG00000104687	GSR	glutathione-disulfide reductase	P00390	0.0012	2.57
ENSG00000149480	MTA2	metastasis associated 1 family member 2	O94776	0.0022	2.56
ENSG00000136938	ANP32B	acidic nuclear phosphoprotein 32 family member B	Q92688	0.003	2.56
ENSG00000100243	CYB5R3	cytochrome b5 reductase 3	P00387	0.00047	2.54
ENSG00000162616	DNAJB4	DnaJ heat shock protein family (Hsp40) member B4	Q9UDY4	0.00051	2.53
ENSG00000197958	RPL12	ribosomal protein L12	P30050	0.00026	2.52
ENSG00000165637	VDAC2	voltage dependent anion channel 2	P45880	0.00074	2.52
ENSG00000070756	PABPC1	poly(A) binding protein cytoplasmic 1	A0A024R9C1	0.001	2.51
ENSG00000100097	LGALS1	galectin 1	P09382	0.0021	2.51
ENSG00000141456	PELP1	proline, glutamate and leucine rich protein 1	B4DEX7	0.011	2.51
ENSG00000130429	ARPC1B	actin related protein 2/3 complex subunit 1B	A4D275	0.0006	2.49
ENSG00000167699	GLOD4	glyoxalase domain containing 4	Q9HC38	0.00038	2.48
ENSG00000137497	NUMA1	nuclear mitotic apparatus protein 1	A0A024R5M9	0.0015	2.48
ENSG00000162521	RBBP4	RB binding protein 4, chromatin remodeling factor	Q09028	0.0022	2.48
ENSG00000142507	PSMB6	proteasome 20S subunit beta 6	A0A087X2I4	0.003	2.48
ENSG00000213639	PPP1CB	protein phosphatase 1 catalytic subunit beta	P62140	0.00085	2.47
ENSG00000103335	PIEZO1	piezo type mechanosensitive ion channel component 1	Q92508	0.0024	2.47
ENSG00000120438	TCP1	t-complex 1	E7EQR6	0.00061	2.45
ENSG00000169710	FASN	fatty acid synthase	P49327	0.00068	2.43
ENSG00000108518	PFN1	profilin 1	P07737	0.0016	2.43
ENSG00000136930	PSMB7	proteasome 20S subunit beta 7	E9KL30	0.0016	2.43
ENSG00000090615	GOLGA3	golgin A3	Q08378	0.022	2.43
ENSG00000106397	PLOD3	procollagen-lysine,2-oxoglutarate 5-dioxygenase 3	O60568	0.00096	2.42
ENSG00000169021	UQCRFS1	ubiquinol-cytochrome c reductase, Rieske iron-sulfur polypeptide 1	P47985	0.0032	2.42
ENSG00000140750	ARHGAP17	Rho GTPase activating protein 17	Q68EM7	0.02	2.42
ENSG00000030066	NUP160	nucleoporin 160	Q12769	0.00074	2.41
ENSG00000240972	MIF	macrophage migration inhibitory factor	I4AY87	0.00078	2.41
ENSG00000130309	COLGALT1	collagen beta(1-O)galactosyltransferase 1	Q8NBJ5	0.001	2.41
ENSG00000126602	TRAP1	TNF receptor associated protein 1	Q12931	0.017	2.41
ENSG00000169504	CLIC4	chloride intracellular channel 4	Q6FIC5	0.00078	2.38
ENSG00000185236	RAB11B	RAB11B, member RAS oncogene family	Q15907	0.00081	2.38

ENSG00000138668	HNRNPD	heterogeneous nuclear ribonucleoprotein D	Q14103	0.00068	2.37
ENSG00000135926	TMBIM1	transmembrane BAX inhibitor motif containing 1	A0A024R472	0.0009	2.36
ENSG00000182774	RPS17	ribosomal protein S17	P08708	0.0012	2.35
ENSG00000001497	LAS1L	LAS1 like ribosome biogenesis factor	Q9Y4W2	0.018	2.35
ENSG00000119707	RBM25	RNA binding motif protein 25	P49756	0.0015	2.34
ENSG00000078369	GNB1	G protein subunit beta 1	B3KVK2	0.0034	2.33
ENSG00000120254	MTHFD1L	methylenetetrahydrofolate dehydrogenase (NADP+ dependent) 1 like	B7ZM99	0.00045	2.32
ENSG00000164924	YWHAZ	tyrosine 3-monooxygenase/tryptophan 5-monooxygenase activation protein zeta	D0PNI1	0.0012	2.31
ENSG00000198363	ASPH	aspartate beta-hydroxylase	Q12797	0.0013	2.31
ENSG00000067113	PLPP1	phospholipid phosphatase 1	A0A024QZS3	0.0064	2.31
ENSG00000133112	TPT1	tumor protein, translationally-controlled 1	A0A0B4J2C3	0.028	2.31
ENSG00000175792	RUVBL1	RuvB like AAA ATPase 1	Q9Y265	0.00078	2.3
ENSG00000135677	GNS	glucosamine (N-acetyl)-6-sulfatase	A0A024RBC5	0.0032	2.3
ENSG00000213719	CLIC1	chloride intracellular channel 1	O00299	0.0012	2.29
ENSG00000169714	CNBP	CCHC-type zinc finger nucleic acid binding protein	P62633	0.0018	2.29
ENSG00000132341	RAN	RAN, member RAS oncogene family	B4DV51	0.0049	2.29
ENSG00000142910	TINAGL1	tubulointerstitial nephritis antigen like 1	Q9GZM7	0.00095	2.28
ENSG00000083845	RPS5	ribosomal protein S5	A0A024R4Q8	0.0017	2.27
ENSG00000157020	SEC13	SEC13 homolog, nuclear pore and COPII coat complex component	P55735	0.0019	2.27
ENSG00000167978	SRRM2	serine/arginine repetitive matrix 2	A0A140VK53	0.0012	2.26
NA	MT-CO2	NA	NA	0.0016	2.25
ENSG00000124570	SERPINB6	serpin family B member 6	A0A024QZX5	0.0024	2.25
ENSG00000161011	SQSTM1	sequestosome 1	Q13501	0.0085	2.25
ENSG00000189403	HMGB1	high mobility group box 1	A0A024RDR0	0.0012	2.24
ENSG00000169100	SLC25A6	solute carrier family 25 member 6	P12236	0.0026	2.24
ENSG00000122565	CBX3	chromobox 3	A4D177	0.0029	2.24
ENSG00000143416	SELENBP1	selenium binding protein 1	Q13228	0.03	2.23
ENSG00000142937	RPS8	ribosomal protein S8	P62241	0.0013	2.22
ENSG00000116005	PCYOX1	prenylcysteine oxidase 1	Q9UHG3	0.0023	2.22
ENSG00000115380	EFEMP1	EGF containing fibulin extracellular matrix protein 1	A0A0S2Z4F1	0.0021	2.21
ENSG00000196230	TUBB	tubulin beta class I	B4DY90	0.0054	2.21
ENSG00000148700	ADD3	adducin 3	Q9UEY8	0.00089	2.2
ENSG00000121774	KHDRBS1	KH RNA binding domain containing, signal transduction associated 1	Q07666	0.0041	2.2
ENSG00000154473	BUB3	BUB3 mitotic checkpoint protein	A0A140VJF3	0.0042	2.19

ENSG00000100504	PYGL	glycogen phosphorylase L	P06737	0.016	2.18
ENSG00000107862	GBF1	golgi brefeldin A resistant guanine nucleotide exchange factor 1	Q92538	0.0013	2.16
ENSG00000203879	GDI1	GDP dissociation inhibitor 1	A0A0S2Z3X8	0.00073	2.15
ENSG00000101294	HM13	histocompatibility minor 13	A0A0S2Z5V7	0.0014	2.15
ENSG00000176619	LMNB2	lamin B2	Q03252	0.0016	2.15
ENSG00000100280	AP1B1	adaptor related protein complex 1 subunit beta 1	Q10567	0.0063	2.15
ENSG00000148834	GSTO1	glutathione S-transferase omega 1	P78417	0.0012	2.14
ENSG00000168259	DNAJC7	DnaJ heat shock protein family (Hsp40) member C7	Q99615	0.0025	2.14
ENSG00000104325	DECR1	2,4-dienoyl-CoA reductase 1	Q16698	0.0036	2.13
ENSG00000108671	PSMD11	proteasome 26S subunit, non-ATPase 11	O00231	0.0012	2.12
ENSG00000092199	HNRNPC	heterogeneous nuclear ribonucleoprotein C	P07910	0.0022	2.12
ENSG00000165280	VCP	valosin containing protein	P55072	0.0048	2.12
ENSG00000131016	AKAP12	A-kinase anchoring protein 12	Q02952	0.0013	2.11
ENSG00000197111	PCBP2	poly(rC) binding protein 2	Q15366	0.0021	2.11
ENSG00000163513	TGFBR2	transforming growth factor beta receptor 2	D2JYI1	0.0022	2.11
ENSG00000170027	YWHAG	tyrosine 3-monooxygenase/tryptophan 5-monooxygenase activation protein gamma	P61981	0.0028	2.11
ENSG00000076201	PTPN23	protein tyrosine phosphatase non-receptor type 23	B4DST5	0.0055	2.11
ENSG00000125977	EIF2S2	eukaryotic translation initiation factor 2 subunit beta	P20042	0.0047	2.1
ENSG00000127483	HP1BP3	heterochromatin protein 1 binding protein 3	Q5SSJ5	0.0066	2.1
ENSG00000158710	TAGLN2	transgelin 2	P37802	0.013	2.1
ENSG00000112118	MCM3	minichromosome maintenance complex component 3	B4DUQ9	0.028	2.1
ENSG00000188257	PLA2G2A	phospholipase A2 group IIA	A0A024RA96	0.036	2.1
ENSG00000109501	WFS1	wolframin ER transmembrane glycoprotein	A0A0S2Z4V6	0.0012	2.09
ENSG00000159176	CSRP1	cysteine and glycine rich protein 1	B4DY28	0.0031	2.09
ENSG00000204628	RACK1	receptor for activated C kinase 1	E9KL35	0.0044	2.09
ENSG00000150753	CCT5	chaperonin containing TCP1 subunit 5	B4DX08	0.0012	2.06
ENSG00000108515	ENO3	enolase 3	P13929	0.0015	2.06
ENSG00000198561	CTNND1	catenin delta 1	O60716	0.002	2.06
ENSG00000100911	PSME2	proteasome activator subunit 2	Q86SZ7	0.0037	2.06
ENSG00000116288	PARK7	Parkinsonism associated deglycase	Q99497	0.0015	2.05
ENSG00000121152	NCAPH	non-SMC condensin I complex subunit H	Q15003	0.0056	2.05
ENSG00000067369	TP53BP1	tumor protein p53 binding protein 1	Q12888	0.025	2.05
ENSG00000166794	PPIB	peptidylprolyl isomerase B	P23284	0.0015	2.04
ENSG00000170323	FABP4	fatty acid binding protein 4	E7DVW4	0.0036	2.04

ENSG00000130158	DOCK6	dedicator of cytokinesis 6	B7Z9U8	0.005	2.03
ENSG00000095380	NANS	N-acetylneuraminate synthase	Q9NR45	0.0038	2.02
ENSG00000170348	TMED10	transmembrane p24 trafficking protein 10	A0A024R6I3	0.0055	2.02
ENSG00000084652	TXLNA	taxilin alpha	P40222	0.0019	2.01
ENSG00000198898	CAPZA2	capping actin protein of muscle Z-line subunit alpha 2	A4D0V4	0.0024	2.01
ENSG00000176986	SEC24C	SEC24 homolog C, COPII coat complex component	A0A024QZM6	0.02	2.01
ENSG00000146731	CCT6A	chaperonin containing TCP1 subunit 6A	P40227	0.0048	2
ENSG00000091164	TXNL1	thioredoxin like 1	O43396	0.0059	2
ENSG00000113569	NUP155	nucleoporin 155	B4DLT2	0.0065	1.99
ENSG00000144746	ARL6IP5	ADP ribosylation factor like GTPase 6 interacting protein 5	A0A024R371	0.034	1.99
ENSG00000113758	DBN1	drebrin 1	Q16643	0.0032	1.97
ENSG00000163131	CTSS	cathepsin S	P25774	0.0017	1.96
ENSG00000107719	PALD1	phosphatase domain containing paladin 1	A0A024QZM5	0.0018	1.96
ENSG00000161057	PSMC2	proteasome 26S subunit, ATPase 2	B7Z571	0.0033	1.96
ENSG00000004700	RECQL	RecQ like helicase	A0A024RAV2	0.0039	1.96
ENSG00000054654	SYNE2	spectrin repeat containing nuclear envelope protein 2	Q8WXH0	0.0048	1.96
ENSG00000140416	TPM1	tropomyosin 1	A0A0K0K1I0	0.0016	1.95
ENSG00000211623	IGKV2D-26	immunoglobulin kappa variable 2D-26	NA	0.027	1.95
ENSG00000110107	PRPF19	pre-mRNA processing factor 19	Q9UMS4	0.002	1.94
ENSG00000062485	CS	citrate synthase	A0A024RB75	0.015	1.94
ENSG00000177674	AGTRAP	angiotensin II receptor associated protein	Q6RW13	0.0029	1.93
ENSG00000166333	ILK	integrin linked kinase	Q13418	0.013	1.93
ENSG00000111144	LTA4H	leukotriene A4 hydrolase	A0A140VK27	0.0018	1.92
ENSG00000140105	WARS1	tryptophanyl-tRNA synthetase 1	A0A024R6K8	0.0019	1.92
ENSG00000075415	SLC25A3	solute carrier family 25 member 3	A0A024RBE8	0.0023	1.92
ENSG00000085832	EPS15	epidermal growth factor receptor pathway substrate 15	B7Z240	0.0068	1.92
ENSG00000138772	ANXA3	annexin A3	P12429	0.0017	1.91
ENSG00000127022	CANX	calnexin	P27824	0.0018	1.91
ENSG00000173267	SNCG	synuclein gamma	F8W754	0.0076	1.91
ENSG00000137509	PRCP	prolylcarboxypeptidase	B7Z7Q6	0.024	1.91
ENSG00000073969	NSF	N-ethylmaleimide sensitive factor, vesicle fusing ATPase	P46459	0.0019	1.9
ENSG00000005022	SLC25A5	solute carrier family 25 member 5	P05141	0.0029	1.9
ENSG00000171793	CTPS1	CTP synthase 1	B4E1E0	0.0038	1.9
ENSG00000175203	DCTN2	dynactin subunit 2	Q13561	0.0066	1.9
ENSG00000168003	SLC3A2	solute carrier family 3 member 2	J3KPF3	0.0028	1.89
ENSG00000114573	ATP6V1A	ATPase H+ transporting V1 subunit A	P38606	0.0031	1.89

ENSG00000058262	SEC61A1	SEC61 translocon subunit alpha 1	B3KME8	0.0051	1.89
ENSG00000119689	DLST	dihydrolipoamide S-succinyltransferase	B7Z6J1	0.012	1.88
ENSG00000167674	HDGFL2	HDGF like 2	Q7Z4V5	0.0055	1.87
ENSG00000170144	HNRNPA3	heterogeneous nuclear ribonucleoprotein A3	B4DDB6	0.0044	1.86
ENSG00000165092	ALDH1A1	aldehyde dehydrogenase 1 family member A1	P00352	0.0046	1.86
ENSG00000168610	STAT3	signal transducer and activator of transcription 3	P40763	0.0047	1.86
ENSG00000145833	DDX46	DEAD-box helicase 46	A0A0C4DG89	0.0065	1.86
ENSG00000005893	LAMP2	lysosomal associated membrane protein 2	P13473	0.012	1.86
ENSG00000102119	EMD	emerin	P50402	0.02	1.86
ENSG00000110090	CPT1A	carnitine palmitoyltransferase 1A	P50416	0.0036	1.85
ENSG00000149084	HSD17B12	hydroxysteroid 17-beta dehydrogenase 12	Q53GQ0	0.019	1.85
ENSG00000151176	PLBD2	phospholipase B domain containing 2	Q8NHP8	0.0044	1.84
ENSG00000125166	GOT2	glutamic-oxaloacetic transaminase 2	P00505	0.0036	1.83
ENSG00000198755	RPL10A	ribosomal protein L10a	P62906	0.0053	1.83
ENSG00000119655	NPC2	NPC intracellular cholesterol transporter 2	A0A024R6C0	0.0074	1.83
ENSG00000067225	PKM	pyruvate kinase M1/2	P14618	0.004	1.81
ENSG00000159140	SON	SON DNA and RNA binding protein	P18583	0.0073	1.81
ENSG00000113643	RARS1	arginyl-tRNA synthetase 1	P54136	0.0051	1.8
ENSG00000104388	RAB2A	RAB2A, member RAS oncogene family	P61019	0.008	1.8
ENSG00000197635	DPP4	dipeptidyl peptidase 4	P27487	0.018	1.8
ENSG00000140740	UQCRC2	ubiquinol-cytochrome c reductase core protein 2	P22695	0.022	1.8
ENSG00000023191	RNH1	ribonuclease/angiogenin inhibitor 1	A0A140VJT8	0.0055	1.79
ENSG00000169223	LMAN2	lectin, mannose binding 2	Q12907	0.0059	1.79
ENSG00000172037	LAMB2	laminin subunit beta 2	A0A024R319	0.0066	1.78
ENSG00000149273	RPS3	ribosomal protein S3	P23396	0.0073	1.78
ENSG00000162402	USP24	ubiquitin specific peptidase 24	Q9UPU5	0.047	1.78
ENSG00000123416	TUBA1B	tubulin alpha 1b	P68363	0.0048	1.77
ENSG00000066777	ARFGEF1	ADP ribosylation factor guanine nucleotide exchange factor 1	A0A024R7X0	0.0079	1.77
ENSG00000147140	NONO	non-POU domain containing octamer binding	A0A0S2Z4Z9	0.026	1.77
ENSG00000132780	NASP	nuclear autoantigenic sperm protein	P49321	0.005	1.76
ENSG00000196497	IPO4	importin 4	B3KT38	0.01	1.76
ENSG00000175130	MARCKSL1	MARCKS like 1	P49006	0.017	1.76
ENSG00000089157	RPLP0	ribosomal protein lateral stalk subunit P0	A0A024RBS2	0.0039	1.75
ENSG00000186468	RPS23	ribosomal protein S23	A8K517	0.0069	1.75

Adjustments of p-values for multiple comparisons were used with Benjamini-Hochberg (BH) correction.

Supplemental Table S6. LEC protein palmitoylated enriched only in siCtrl and insulin treatment condition.

ENSEMBL ID	Description	UNIPROT	Enrichment in siCtrl + Ins	
			p-value	-Log Ratio (+/- HA)
ENSG00000075239	acetyl-CoA acetyltransferase 1	A0A140VJX1	0.0063	1.5
ENSG00000014257	acid phosphatase 3	P15309	0.035	2.46
ENSG00000137845	ADAM metallopeptidase domain 10	A0A024R5U5	0.042	1.01
ENSG00000162618	adhesion G protein-coupled receptor L4	Q9HBW9	2.00E-06	5.66
ENSG00000119711	aldehyde dehydrogenase 6 family member A1	Q02252	0.036	1.06
ENSG00000084674	apolipoprotein B	P04114	0.0062	2.7
ENSG00000145349	calcium/calmodulin dependent protein kinase II delta	A0A024RDK3	0.019	1.43
ENSG00000147419	coiled-coil domain containing 25	G3V121	0.04	2.05
ENSG00000135218	CD36 molecule	A4D1B1	0.0014	2.13
ENSG00000004468	CD38 molecule	B4E006	8.00E-05	3.66
ENSG00000026508	CD44 molecule (Indian blood group)	P16070	4.10E-06	4.36
ENSG00000013297	claudin 11	O75508	7.10E-06	4.63
ENSG00000153551	CKLF like MARVEL transmembrane domain containing 7	A0A024R2L3	7.20E-06	4.95
ENSG00000158270	collectin subfamily member 12	Q5KU26	0.013	1.48
ENSG00000136160	endothelin receptor type B	P24530	0.0066	1.82
ENSG00000181104	coagulation factor II thrombin receptor	P25116	2.70E-05	3.67
ENSG00000087086	ferritin light chain	P02792	0.0073	1.76
ENSG00000198380	glutamine--fructose-6-phosphate transaminase 1	Q06210	0.026	1.16
ENSG00000196329	GTPase, IMAP family member 5	A0A090N8P9	0.022	1.64
ENSG00000139433	glycolipid transfer protein	A0A024RBI7	0.0011	2.78
ENSG00000135821	glutamate-ammonia ligase	A8YXX4	0.021	4.1
ENSG00000088256	G protein subunit alpha 11	P29992	3.70E-05	4.08
ENSG00000120063	G protein subunit alpha 13	Q14344	2.40E-06	5.81
ENSG00000065135	G protein subunit alpha i3	P08754	0.00078	2.18
ENSG00000156052	G protein subunit alpha q	A0A024R240	8.20E-07	5.91
ENSG00000147533	golgin A7	Q7Z5G4	5.90E-06	4.62
ENSG00000115159	glycerol-3-phosphate dehydrogenase 2	P43304	0.024	1.18
ENSG00000074696	3-hydroxyacyl-CoA dehydratase 3	Q9P035	0.0012	2.28
ENSG00000063854	hydroxyacylglutathione hydrolase	Q16775	0.022	3.4
ENSG00000004961	holocytochrome c synthase	A0A024RBY9	0.00077	2.33
ENSG00000148634	HECT and RLD domain containing E3 ubiquitin protein ligase 4	Q5GLZ8	0.007	1.36
NA	major histocompatibility complex, class I, H (pseudogene)	NA	0.043	1.71
ENSG00000103415	heme oxygenase 2	P30519	0.012	1.28
ENSG00000197081	insulin like growth factor 2 receptor	P11717	0.034	1.19
ENSG00000136688	interleukin 36 gamma	Q9NZH8	0.00084	2.96
ENSG00000167754	kallikrein related peptidase 5	Q9Y337	0.0093	2.04

ENSG00000213625	leptin receptor overlapping transcript	A0A087X0N2	8.20E-05	3.42
ENSG00000205076	galectin 7	P47929	0.041	2.7
ENSG00000100258	lipase maturation factor 2	Q9BU23	4.20E-05	3.65
ENSG00000254087	LYN proto-oncogene, Src family tyrosine kinase	P07948	0.00022	2.92
ENSG00000156026	mitochondrial calcium uniporter	Q8NE86	0.0053	1.99
ENSG00000157227	matrix metallopeptidase 14	P50281	7.70E-05	2.96
ENSG00000262814	mitochondrial ribosomal protein L12	P52815	0.019	1.34
ENSG00000204568	mitochondrial ribosomal protein S18B	B0S7P4	0.0011	2.52
ENSG00000125148	metallothionein 2A	P02795	0.00089	3.08
ENSG00000147649	metadherin	A0A024R9D2	0.00078	2.05
ENSG00000144959	neutral cholesterol ester hydrolase 1	A0A0A0MTJ9	4.60E-05	3.82
ENSG00000139180	NADH:ubiquinone oxidoreductase subunit A9	Q16795	0.034	2.27
ENSG00000135124	purinergic receptor P2X 4	Q99571	9.10E-05	3.2
ENSG00000184363	plakophilin 3	Q9Y446	0.031	2.61
ENSG00000073008	PVR cell adhesion molecule	P15151	0.0049	1.65
ENSG00000123728	RAP2C, member of RAS oncogene family	Q9Y3L5	6.50E-06	4.04
ENSG00000239306	RNA binding motif protein 14	A0A0S2Z567	0.038	2.09
ENSG00000131378	raftlin, lipid raft linker 1	Q14699	0.00043	2.5
ENSG00000205937	RNA binding protein with serine rich domain 1	D3DU92	0.0054	2.53
ENSG00000063177	ribosomal protein L18	A0A024QZD1	0.0015	1.81
ENSG00000144713	ribosomal protein L32	A0A024R2G7	0.0093	1.35
ENSG00000109475	ribosomal protein L34	A0A024RDH8	0.029	1.27
ENSG00000130255	ribosomal protein L36	Q9Y3U8	0.012	1.55
ENSG00000174444	ribosomal protein L4	P36578	0.0096	1.32
ENSG00000197728	ribosomal protein S26	A0A024RB14	0.0096	2.19
ENSG00000170889	ribosomal protein S9	A0A024R4M0	0.0016	2.18
ENSG00000126458	RAS related	A0A024QZF2	7.40E-07	5.94
ENSG00000143546	S100 calcium binding protein A8	P05109	0.046	2.79
ENSG00000140497	secretory carrier membrane protein 2	A8K769	8.40E-06	4.1
ENSG00000183291	selenoprotein F	O60613	0.00057	2.26
ENSG00000057149	serpin family B member 3	P29508	0.0068	3.7
ENSG00000206073	serpin family B member 4	P48594	0.023	3.42
ENSG00000155380	solute carrier family 16 member 1	A0A024R0H1	0.01	1.86
ENSG00000146411	solute carrier family 2 member 12	Q8TD20	0.00055	3.36
ENSG00000104852	small nuclear ribonucleoprotein U1 subunit 70	P08621	2.70E-05	3.46
ENSG00000133226	serine and arginine repetitive matrix 1	B7Z7U0	1.90E-05	3.65
ENSG00000116754	serine and arginine rich splicing factor 11	Q05519	1.10E-05	4.73
ENSG00000116350	serine and arginine rich splicing factor 4	Q08170	0.025	1.59
ENSG00000168394	transporter 1, ATP binding cassette subfamily B member	A0A0S2Z5A6	0.00028	2.39
ENSG00000130193	thioesterase superfamily member 6	Q8WUY1	0.0058	1.76
ENSG00000066654	THUMP domain containing 1	A0A024R388	0.018	1.72
ENSG00000099203	transmembrane p24 trafficking protein 1	Q13445	3.30E-06	4.91

ENSG00000109084	transmembrane protein 97	Q5BJF2	0.0093	3.16
ENSG00000166479	thioredoxin related transmembrane protein 3	Q96JJ7	0.027	1.08
ENSG00000187688	transient receptor potential cation channel subfamily V member 2	Q9Y5S1	0.00014	2.73
ENSG00000000003	tetraspanin 6	A0A024RCI0	0.0021	2.85
ENSG00000127824	tubulin alpha 4a	P68366	0.0081	1.63
ENSG00000184470	thioredoxin reductase 2	E7EWK1	9.70E-05	3.14
ENSG00000025708	thymidine phosphorylase	B2RBL3	0.048	2.16
ENSG00000072401	ubiquitin conjugating enzyme E2 D1	A0A087WW00	0.00073	3.11
ENSG00000198833	ubiquitin conjugating enzyme E2 J1	Q9Y385	0.00031	2.76
ENSG00000159202	ubiquitin conjugating enzyme E2 Z	Q9H832	0.00066	2.24
ENSG00000114062	ubiquitin protein ligase E3A	Q05086	4.10E-05	3.79
ENSG00000074755	zinc finger ZZ-type and EF-hand domain containing 1	O43149	0.0096	1.45
