# Supplementary material for: Insulin regulates lymphatic endothelial integrity via palmitoylation
Source: J Lipid Res. 2025 Mar 11;66(4):100775. doi: 10.1016/j.jlr.2025.100775 (PMC12002826; doi:10.1016/j.jlr.2025.100775)
Supplement: Supplementary Figures [file mmc2.pdf]

Supplemental Figure 1

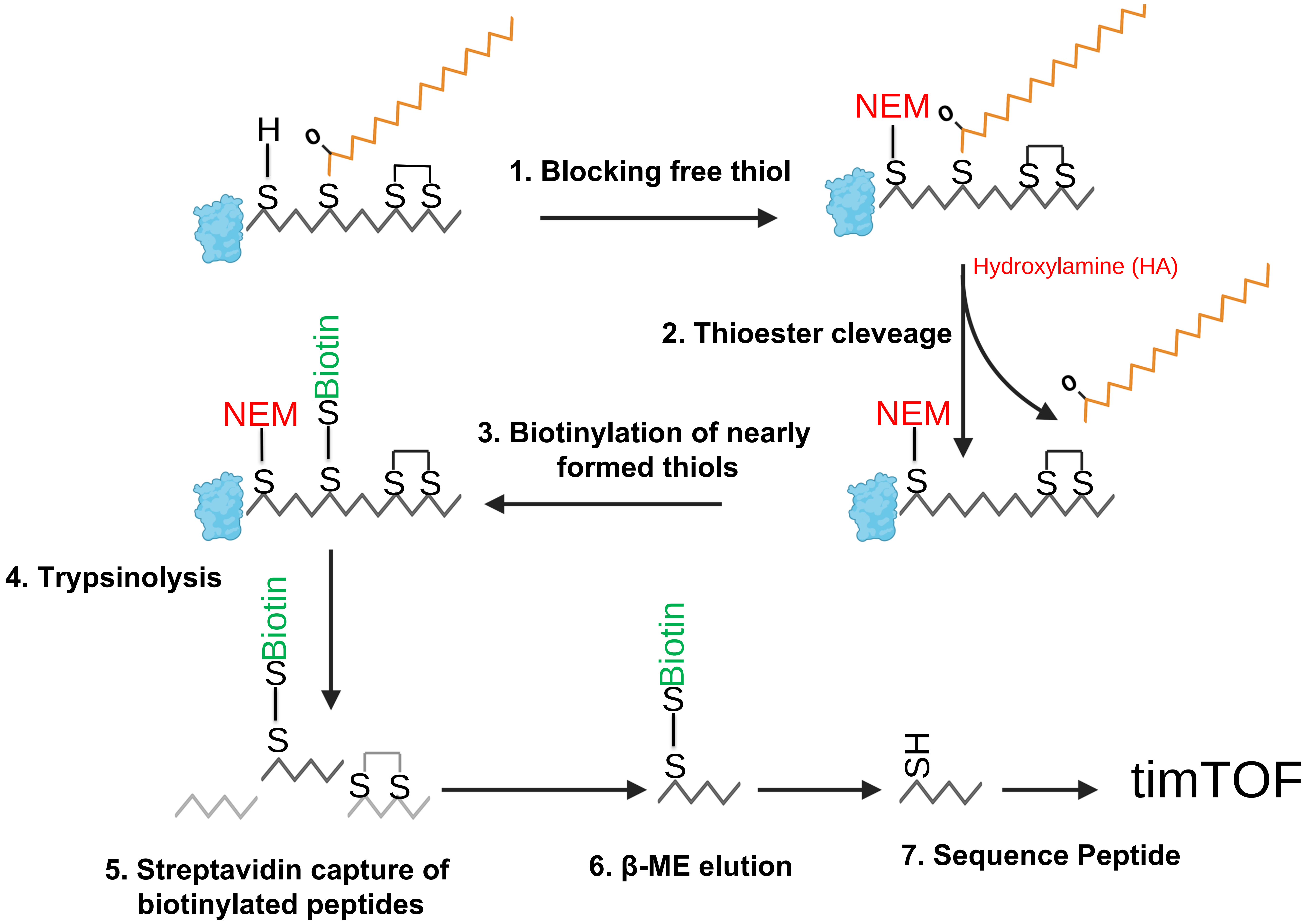

Supplemental Figure 2

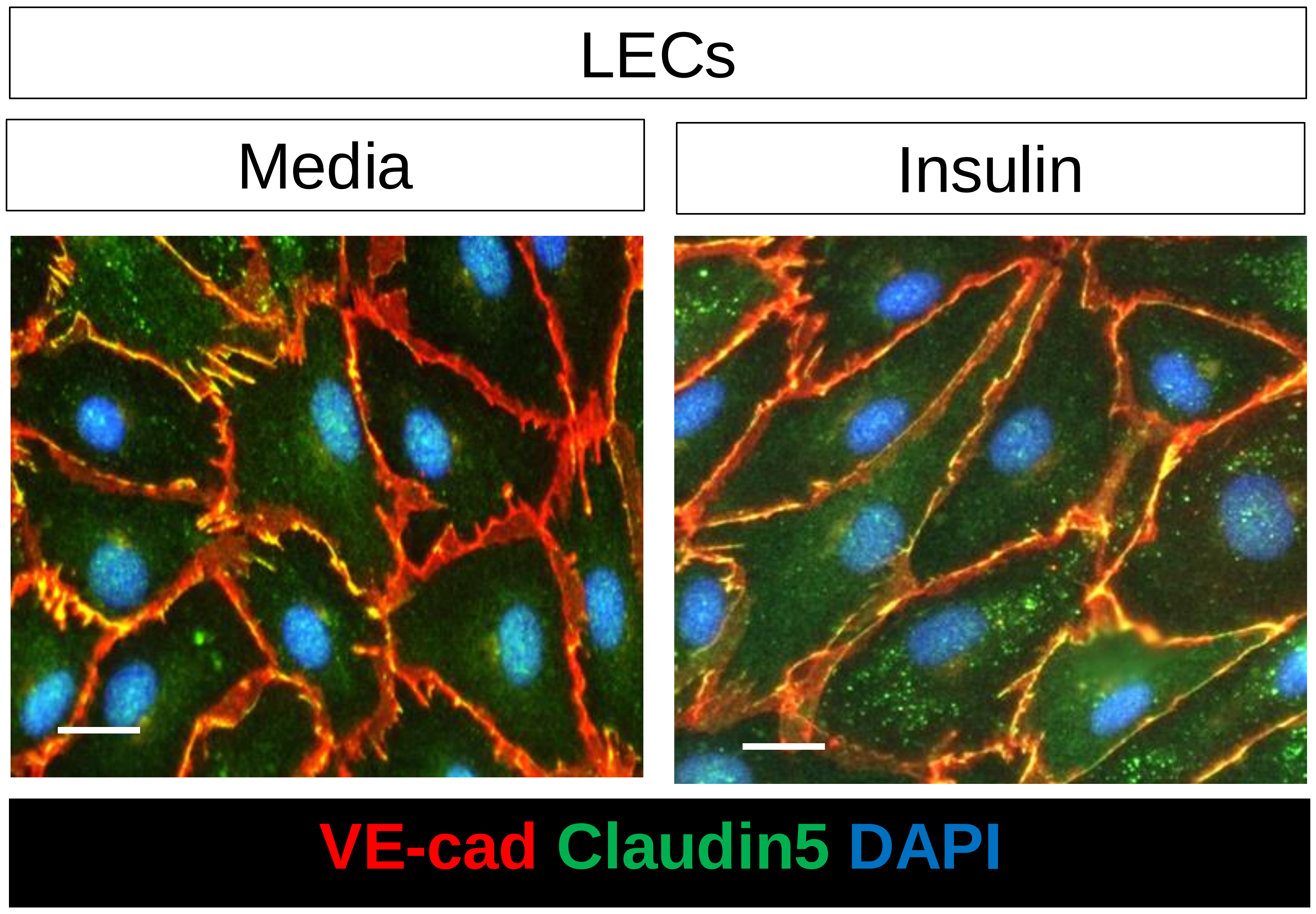

Supplementary Figure 3

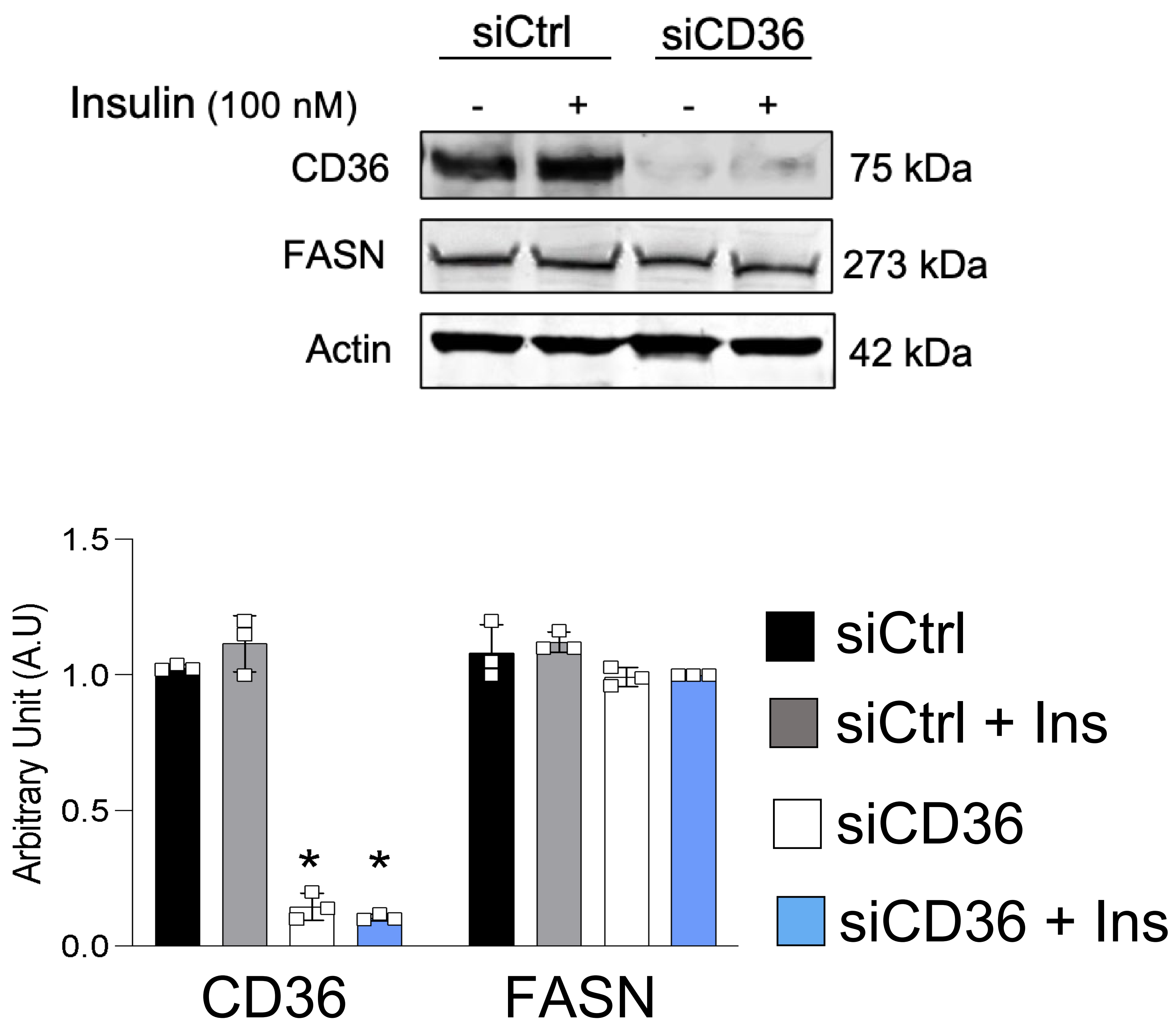

Supplemental Figure 4

a

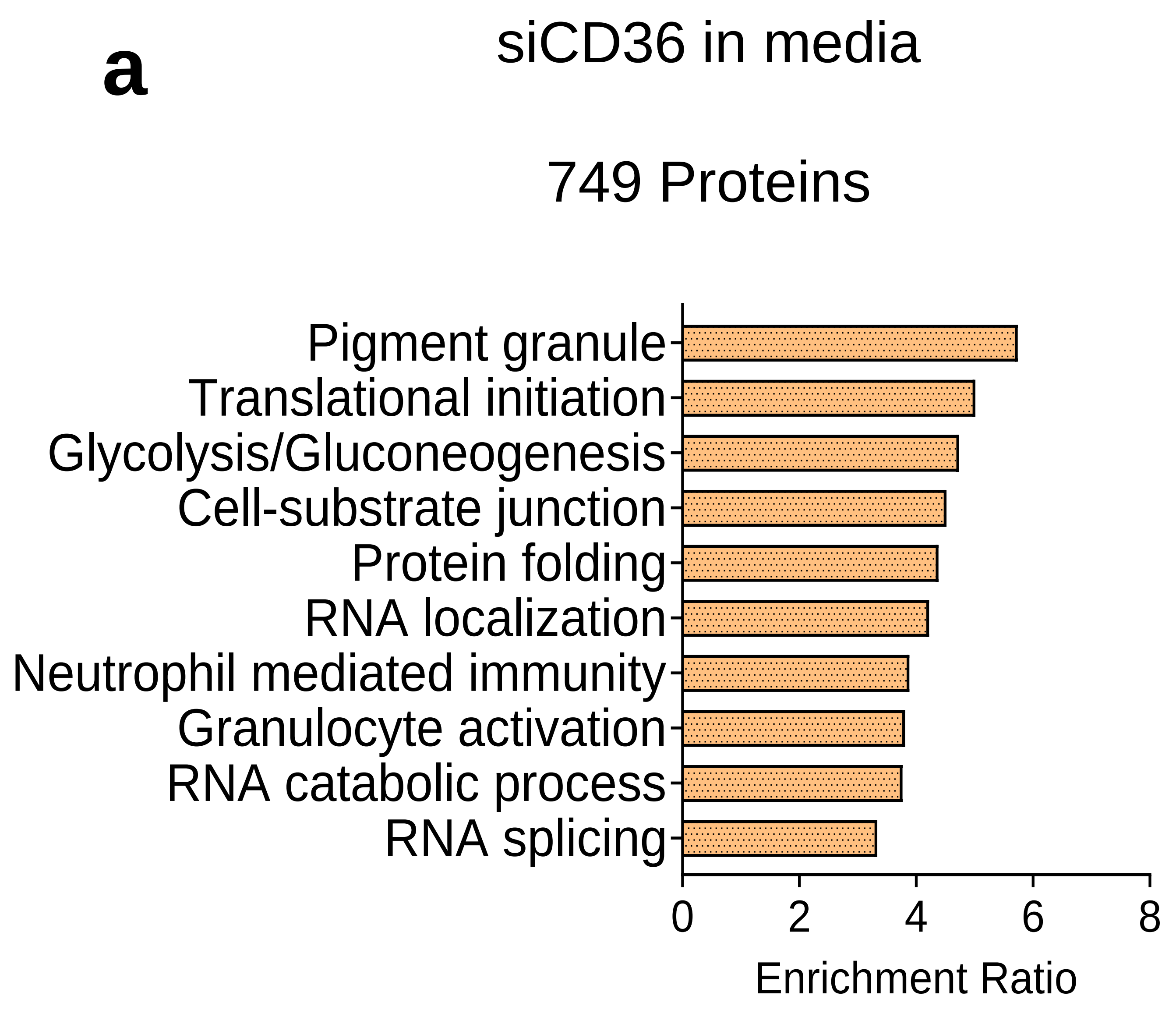

b

UNIPROT ID	Gene Symbol	Description	*p*-value	-Log Ratio (+/-HA)
A0A087WWM0	TRAPPC3	Trafficking protein particle complex subunit 3	2.70E-07	7.2
O14828	SCAMP3	Secretory carrier membrane protein 3	3.40E-07	6.63
Q9Y287	ITM2B	Integral membrane protein 2B	3.10E-07	6.58
O75955	FLOT1	Flotillin 1	6.90E-07	6.57
Q9NRQ2	PLSCR4	Phospholipid scramblase 4	5.10E-07	6.53
E9PJK1	CD81	CD81 molecule	3.40E-07	6.44
P62070	RRAS2	RAS related 2	6.10E-07	6.44
P14209	CD99	CD99 molecule (Xg blood group)	3.20E-07	6.35
Q13530	SERINC3	Serine incorporator 3	5.10E-07	6.3
Q8N357	SLC35F6	Solute carrier family 35 member F6	1.00E-06	6.2

c

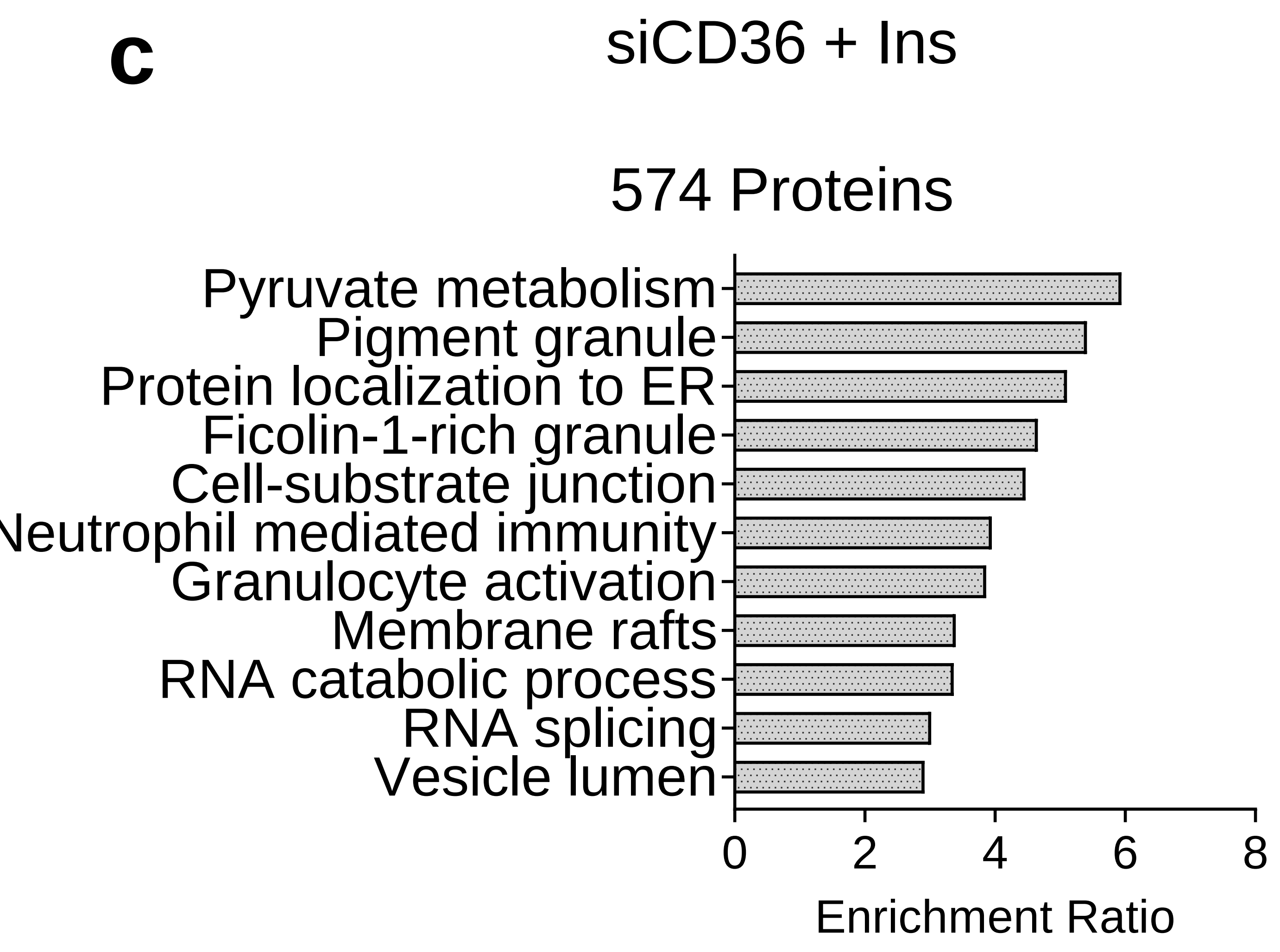

d

UNIPROT ID	Gene Symbol	Description	*p*-value	-Log Ratio (+/-HA)
Q8N357	SLC35F6	Solute carrier family 35 member F6	1.7E-07	7.76
P14209	CD99	CD99 molecule (Xg blood group)	1.4E-07	7.02
A0A087WWM0	TRAPPC3	Trafficking protein particle complex subunit 3	4.1E-07	6.82
Q9NRX5	SERINC1	Serine incorporator 1	3.6E-07	6.71
A0A024QYW3	PLP2	Proteolipid protein 2	2.1E-06	6.67
Q9Y287	ITM2B	Integral membrane protein 2B	2.9E-07	6.63
E9PJK1	CD81	CD81 molecule	3.2E-07	6.49
A0A024RCB3	CD151	CD151 molecule (Raph blood group)	1.2E-06	6.41
A0A024R3I5	MCAM	Melanoma cell adhesion molecule	5.6E-07	6.27
P62745	RHOB	Ras homolog family member B	1.6E-06	6.24
